# Supplementary figures and images for: Assessment of control strategies against Clonorchis sinensis infection based on a multi-group dynamic transmission model
Source: PLoS Negl Trop Dis. 2020 Mar 27;14(3):e0008152. doi: 10.1371/journal.pntd.0008152 (PMC7156112; doi:10.1371/journal.pntd.0008152)

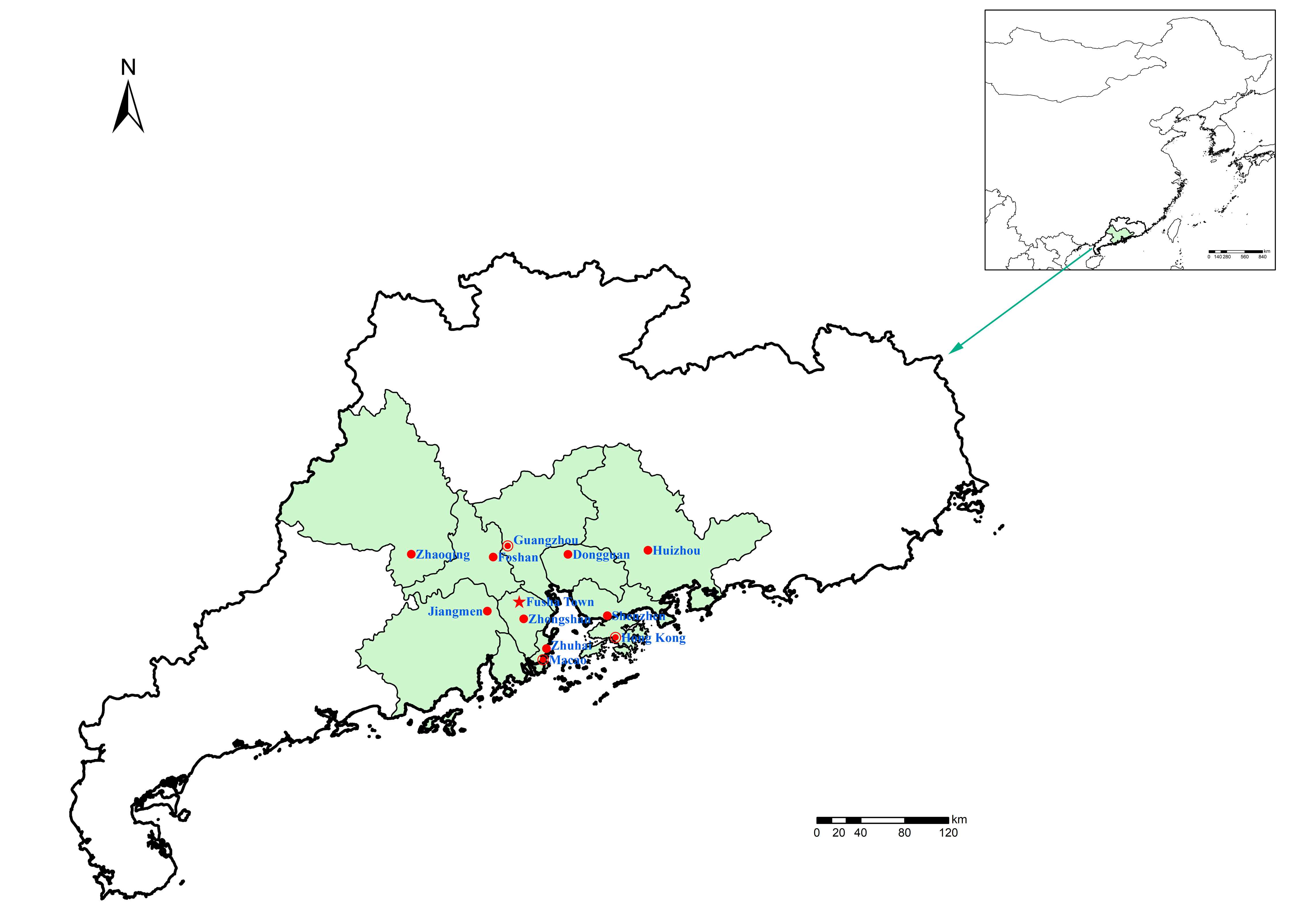

Supplement: S1 Fig — The red star indicates the location of the town, and the green areas indicates the Pearl River Delta. Data for boundaries of the administrative divisions were downloaded from the GADM (https://gadm.org/). The coordinates of Fusha Town and other cities was obtained through the USGS LandsatLook (https://landsatlook.usgs.gov/). Based on the above data, the maps were produced using ArcGIS 10.2. (TIF) [file pntd.0008152.s001.tif]

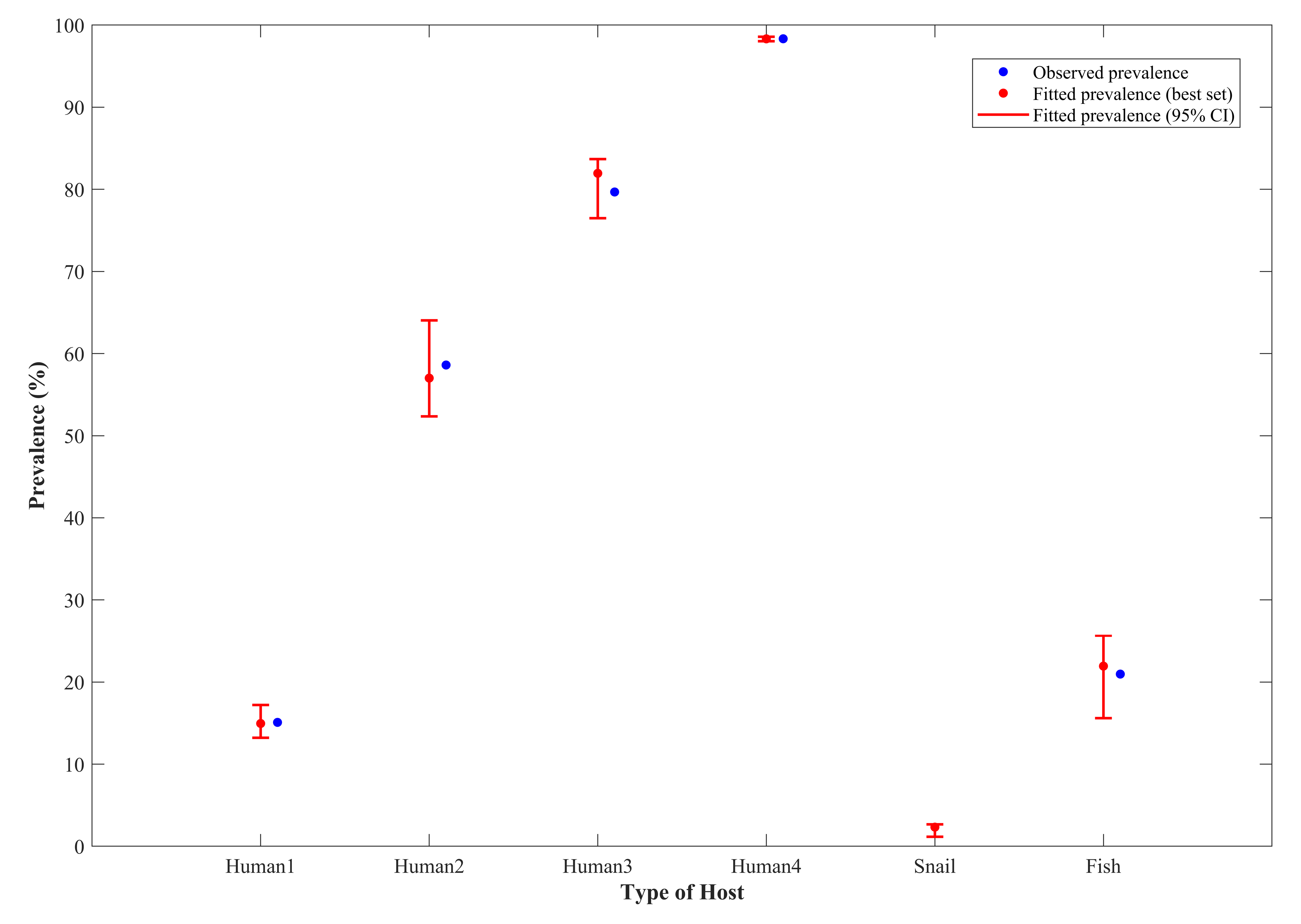

Supplement: S2 Fig — Human1, Human2, Human3 and Human4 in X-axis indicate human groups who seldom, moderately, often and very often eat raw or uncooked fish, respectively. (TIF) [file pntd.0008152.s002.tif]

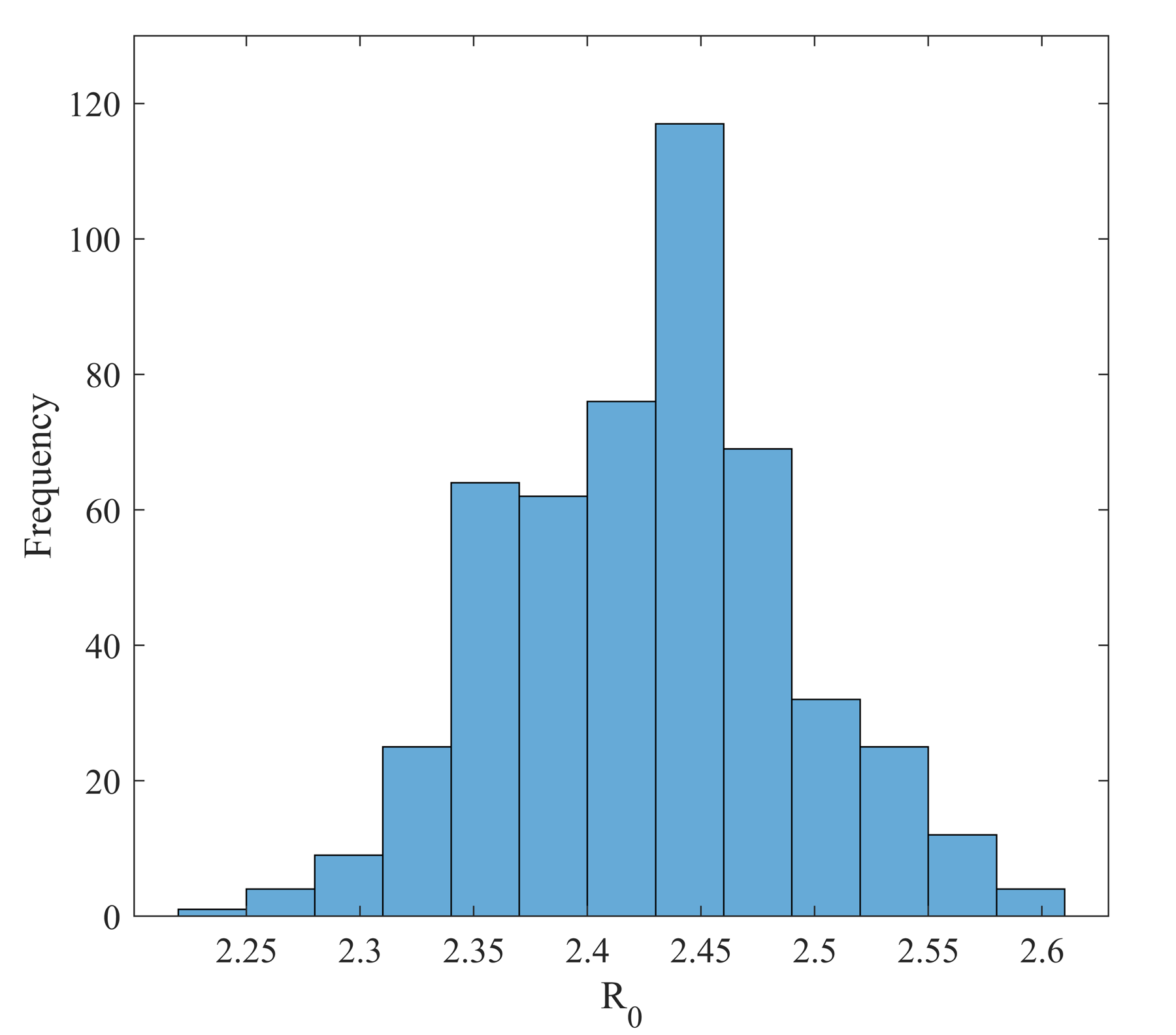

Supplement: S3 Fig — (TIF) [file pntd.0008152.s003.tif]

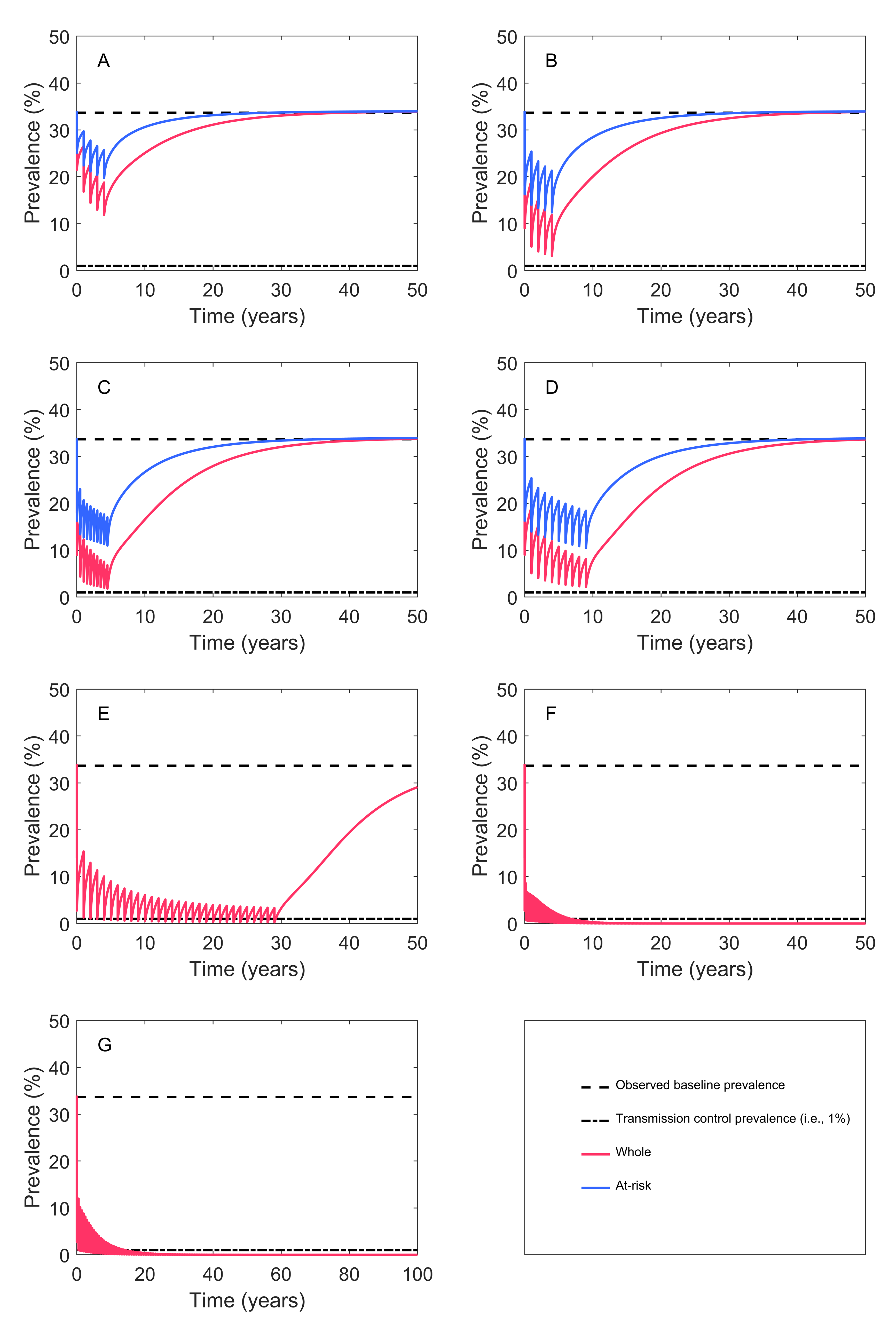

Supplement: S4 Fig — The parameters were set to the best set of parameter estimates. Red lines indicate chemotherapy focus on whole population while blue lines on at-risk groups with raw-fish-eating behaviors. (A) F = 1, D = 5, red line: Cm,1 = Cm,2 = Cm,3 = Cm,4 = 0.4, and blue line: Cm,1 = 0, Cm,2 = Cm,3 = Cm,4 = 0.4; (B) F = 1, D = 5, red line: Cm,1 = Cm,2 = Cm,3 = Cm,4 = 0.8, and blue line: Cm,1 = 0, Cm,2 = Cm,3 = Cm,4 = 0.8; (C) F = 0.5, D = 5, red line: Cm,1 = Cm,2 = Cm,3 = Cm,4 = 0.8, and blue line: Cm,1 = 0, Cm,2 = Cm,3 = Cm,4 = 0.8; (D) F = 1, D = 10, red line: Cm,1 = Cm,2 = Cm,3 = Cm,4 = 0.8, and blue line: Cm,1 = 0, Cm,2 = Cm,3 = Cm,4 = 0.8; (E) F = 1, D = 30, Cm,1 = Cm,2 = Cm,3 = Cm,4 = 1.0. (F) F = 0.2, D = 33, Cm,1 = Cm,2 = Cm,3 = Cm,4 = 1.0. (G) F = 0.5, D = 70, Cm,1 = Cm,2 = Cm,3 = Cm,4 = 1.0. Cm,i, F and D indicate the coverage in the ith group of population, treatment times per year and duration years of intervention, respectively. (TIF) [file pntd.0008152.s004.tif]

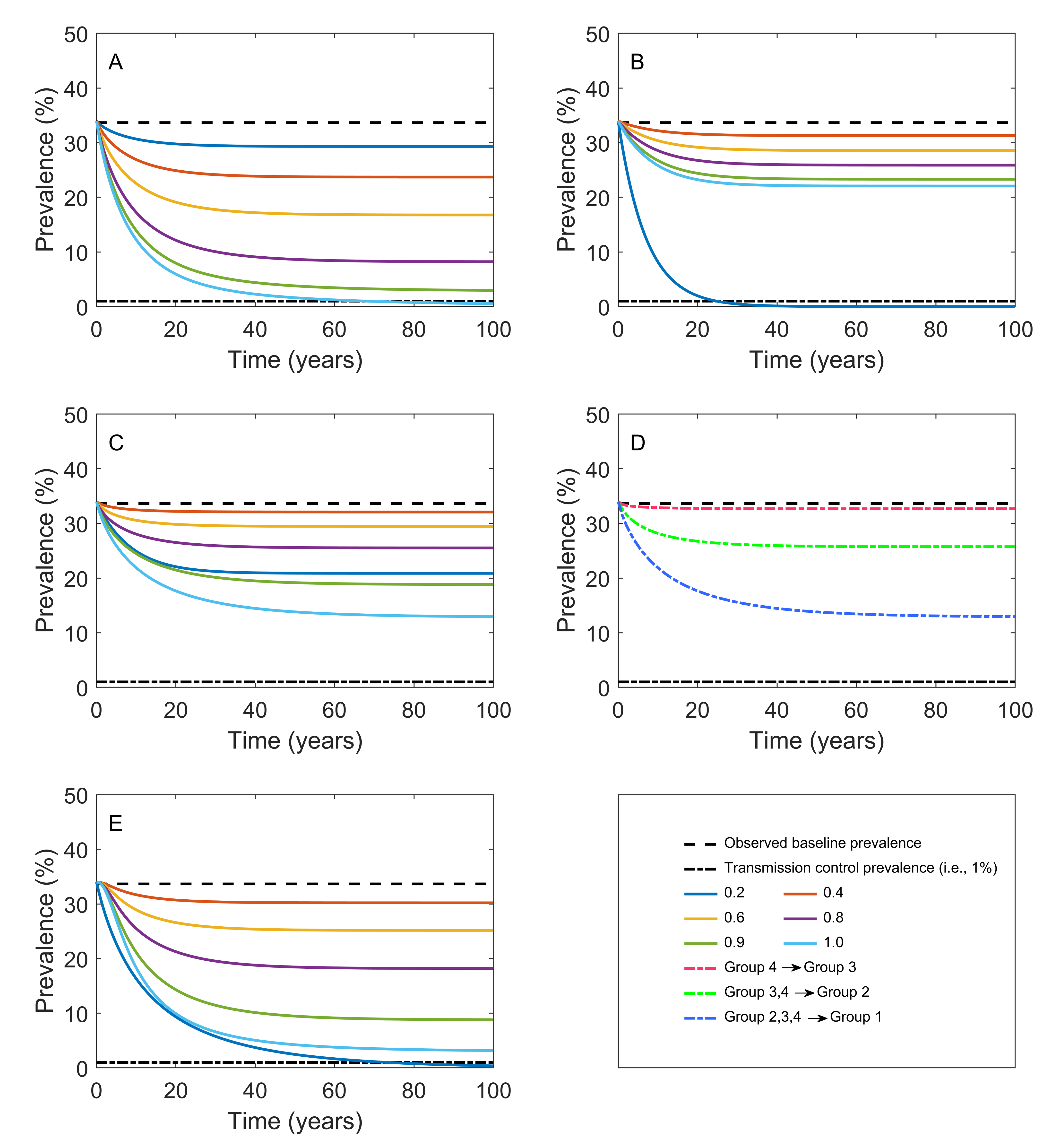

Supplement: S5 Fig — The parameters were set to the best set of parameter estimates. The improvement rates were set to 0.2, 0.4, 0.6, 0.8, 0.9 and 1.0. (A)-(D) represent the simulations under different improvement rates of IEC: (A) IEC focus both on improvement of hygiene habits and stopping people’s behavior of raw-fish-consumption; (B) IEC only focus on improvement of hygiene habits; (C) IEC only focus on stopping people’s behavior of raw-fish-consumption; (D) IEC only focus on changing the behavior of raw-fish-consumption on special groups, with red line indicates people who previous ate raw fish very often changed their behavior to eat often, with green line indicates people who previous ate raw fish very often or often changed their behavior to eat moderately, and with blue line indicates people who previous have raw-fish-eating behavior stopped eat raw fish. (E) represent the simulations under different improvement rates of environmental modification. (TIF) [file pntd.0008152.s005.tif]
