# Supplementary material for: Assessment of control strategies against Clonorchis sinensis infection based on a multi-group dynamic transmission model
Source: PLoS Negl Trop Dis. 2020 Mar 27;14(3):e0008152. doi: 10.1371/journal.pntd.0008152 (PMC7156112; doi:10.1371/journal.pntd.0008152)
Supplement: S1 Table — (DOCX) [file pntd.0008152.s006.docx]

**S1 Table. Resident population of Fusha Town from 2010 to 2017*.**

| Fusha Town | 2010 | 2011 | 2012 | 2013 | 2014 | 2015 | 2016 | 2017 |
| --- | --- | --- | --- | --- | --- | --- | --- | --- |
| Resident population | 57604 | 57889 | 58059 | 58246 | 58502 | 58960 | 59180 | 59593 |

*Data was obtained from Zhongshan Statistical Yearbook 2018, available at: http://stats.zs.gov.cn/zwgk/tjxx/tjnj/content/post_340034.html (cited 2020 Jan 13).
