# Supplementary material for: Assessment of control strategies against Clonorchis sinensis infection based on a multi-group dynamic transmission model
Source: PLoS Negl Trop Dis. 2020 Mar 27;14(3):e0008152. doi: 10.1371/journal.pntd.0008152 (PMC7156112; doi:10.1371/journal.pntd.0008152)
Supplement: S2 Table — (DOCX) [file pntd.0008152.s007.docx]

**S2 Table. Prior triangular distributions for unknown parameters and their sources (unit: day^-1^).**

| Parameter | Mode [Range] | Reference |
| --- | --- | --- |
| $p_{1}$ | 0.67 [0.57-0.77] | [1] |
| $p_{2}$ | 0.18 [0.08-0.28] | [1] |
| $p_{3}$ | 0.10 [0.05-0.15] | [1] |
| $p_{4}$ | 0.05 [0.04-0.06] | [1] |
| $\lambda_{h,i} (i=1,2,3,4)$ | $p_{i}^{'}\times N_{h}\times\mu_{h}$* | See S4 text |
| $\lambda_{f}$ | 2191.78 [219.18-4383.56] | [2-4] |
| $\beta_{h,1}$ | 2.81×10^-10^ [2.81×10^-11^-5.63×10^-10^] | See S4 text |
| $\beta_{s}$ | 2.71×10^-9^ [2.71×10^-10^-5.43×10^-9^] | See S4 text |
| $\beta_{f}$ | 1.87×10^-8^ [1.87×10^-9^-3.75×10^-8^] | See S4 text |
| $c_{2}$ | 8 [3-13] | See S4 text |
| $c_{3}$ | 22 [17-32] | See S4 text |
| $c_{4}$ | 332 [312-352] | See S4 text |
| $\mu_{f}$ | 1/(1.5×365) [1/(3×365)- 1/(1×365)] | [2] |
| $\gamma_{1}$ | 0.140/365 [0.014/365-0.200/365] | [5] |

*$p_{i}^{'} (i=1,2,3,4)$ is adjusted proportion of $p_{i}$, so that the sum of $p_{i}^{'}$ for all groups equals to one.

**References**

1. Du SR, Huang JX, Li HW. [Epidemiology of clonorchiasis in the towns near the Pearl River Delta] (author’s tranl). South China J Prev Med. 2015;41(3):273-5. Chinese.
2. Liu JJ, Zhang JY, Yuan H. [Investigation and analysis of cost-benefit of freshwater fish culture]. Chinese Fisheries Economics. 2017;35(1):18-27. Chinese.
3. Xie XQ [Internet]. [The rent of fish pond in Zhongshan, Guangdong Province is up to 4750 yuan per mu, which breaking the record ] (author’s tranl). Nan Fang Nong Cun Bao. c2019 [cited 2019 Oct 20]. Available from: http://www.bbwfish.com/article.asp?artid=86551. Chinese.
4. [How many fish can be raised in one mu of fishpond?] (author’s tranl) [Internet]. c2019 [cited 2019 Oct 20]. Available from: <https://zhidao.baidu.com/question/1430988985257871219.html>. Chinese.
5. Ma JQ, Chen GQ, Tang X, Zuo ZH, Cao FP, Gong F, et al. [Survey of Life Habits and Health Knowledge of the People in Epidemic Region of Clonorchiasis]. Re Dai Yi Xue Za Zhi. 2008;8(8):858-60,865. Chinese.
