# Supplementary material for: Assessment of control strategies against Clonorchis sinensis infection based on a multi-group dynamic transmission model
Source: PLoS Negl Trop Dis. 2020 Mar 27;14(3):e0008152. doi: 10.1371/journal.pntd.0008152 (PMC7156112; doi:10.1371/journal.pntd.0008152)
Supplement: S3 Table — (DOCX) [file pntd.0008152.s008.docx]

**S3 Table. Results of simulations applied single chemotherapy strategies with different coverages, frequencies and durations targeted on different populations*.**

| Population | Strategy | | | | | |  | Effectiveness | | | | | | | | |
| --- | --- | --- | --- | --- | --- | --- | --- | --- | --- | --- | --- | --- | --- | --- | --- | --- |
|  | $C_{m,1}$ | $C_{m,2}$ | $C_{m,3}$ | $C_{m,4}$ | $F$ | $D$ |  | $R_{c}$ | $P_{s5}$ | $P_{s10}$ | $P_{s15}$ | $r_{s5}$ | $r_{s10}$ | $r_{s15}$ | $Y_{5\%}$ | $Y_{1\%}$ |
| Whole^#^ | 0.4 | 0.4 | 0.4 | 0.4 | 0.5 | 1 | 1.26 | | 27.70 | 30.50 | 32.03 | 18.48 | 10.23 | 5.75 | - | - |
|  | 0.4 | 0.4 | 0.4 | 0.4 | 0.5 | 2 | 1.26 | | 23.29 | 27.93 | 30.56 | 31.47 | 17.81 | 10.08 | - | - |
|  | 0.4 | 0.4 | 0.4 | 0.4 | 0.5 | 3 | 1.26 | | 19.41 | 25.61 | 29.21 | 42.87 | 24.64 | 14.04 | - | - |
|  | 0.4 | 0.4 | 0.4 | 0.4 | 0.5 | 5 | 1.26 | | 11.16 | 20.90 | 26.37 | 67.16 | 38.48 | 22.40 | - | - |
|  | 0.4 | 0.4 | 0.4 | 0.4 | 0.5 | 10 | 1.26 | | 11.16 | 8.14 | 17.58 | 67.16 | 76.05 | 48.27 | - | - |
|  | 0.4 | 0.4 | 0.4 | 0.4 | 0.5 | 20 | 1.26 | | 11.16 | 8.14 | 7.06 | 67.16 | 76.05 | 79.23 | 10.48 | - |
|  | 0.4 | 0.4 | 0.4 | 0.4 | 1.0 | 1 | 1.52 | | 30.48 | 32.07 | 32.91 | 10.31 | 5.63 | 3.15 | - | - |
|  | 0.4 | 0.4 | 0.4 | 0.4 | 1.0 | 2 | 1.52 | | 27.35 | 30.33 | 31.93 | 19.51 | 10.76 | 6.04 | - | - |
|  | 0.4 | 0.4 | 0.4 | 0.4 | 1.0 | 3 | 1.52 | | 24.32 | 28.62 | 30.96 | 28.44 | 15.76 | 8.88 | - | - |
|  | 0.4 | 0.4 | 0.4 | 0.4 | 1.0 | 5 | 1.52 | | 17.49 | 25.08 | 28.91 | 48.52 | 26.19 | 14.91 | - | - |
|  | 0.4 | 0.4 | 0.4 | 0.4 | 1.0 | 10 | 1.52 | | 17.49 | 14.07 | 22.34 | 48.52 | 58.61 | 34.25 | - | - |
|  | 0.4 | 0.4 | 0.4 | 0.4 | 1.0 | 20 | 1.52 | | 17.49 | 14.07 | 12.81 | 48.52 | 58.61 | 62.32 | - | - |
|  | 0.4 | 0.4 | 0.4 | 0.4 | 2.0 | 1 | 1.79 | | 30.48 | 32.07 | 32.91 | 10.31 | 5.63 | 3.15 | - | - |
|  | 0.4 | 0.4 | 0.4 | 0.4 | 2.0 | 2 | 1.79 | | 30.48 | 32.07 | 32.91 | 10.31 | 5.63 | 3.15 | - | - |
|  | 0.4 | 0.4 | 0.4 | 0.4 | 2.0 | 3 | 1.79 | | 26.61 | 29.99 | 31.75 | 21.68 | 11.74 | 6.58 | - | - |
|  | 0.4 | 0.4 | 0.4 | 0.4 | 2.0 | 5 | 1.79 | | 21.33 | 27.61 | 30.40 | 37.23 | 18.75 | 10.54 | - | - |
|  | 0.4 | 0.4 | 0.4 | 0.4 | 2.0 | 10 | 1.79 | | 21.33 | 21.22 | 26.89 | 37.23 | 37.55 | 20.88 | - | - |
|  | 0.4 | 0.4 | 0.4 | 0.4 | 2.0 | 20 | 1.79 | | 21.33 | 21.22 | 17.67 | 37.23 | 37.55 | 48.00 | - | - |
|  | 0.6 | 0.6 | 0.6 | 0.6 | 0.5 | 1 | 1.07 | | 25.17 | 29.00 | 31.17 | 25.94 | 14.65 | 8.28 | - | - |
|  | 0.6 | 0.6 | 0.6 | 0.6 | 0.5 | 2 | 1.07 | | 20.51 | 26.17 | 29.53 | 39.64 | 22.98 | 13.09 | - | - |
|  | 0.6 | 0.6 | 0.6 | 0.6 | 0.5 | 3 | 1.07 | | 16.52 | 23.62 | 28.02 | 51.40 | 30.50 | 17.54 | - | - |
|  | 0.6 | 0.6 | 0.6 | 0.6 | 0.5 | 5 | 1.07 | | 8.20 | 18.30 | 24.68 | 75.86 | 46.16 | 27.36 | 3.01 | - |
|  | 0.6 | 0.6 | 0.6 | 0.6 | 0.5 | 10 | 1.07 | | 8.20 | 5.31 | 14.60 | 75.86 | 84.37 | 57.04 | 3.01 | - |
|  | 0.6 | 0.6 | 0.6 | 0.6 | 0.5 | 20 | 1.07 | | 8.20 | 5.31 | 4.14 | 75.86 | 84.37 | 87.83 | 3.01 | - |
|  | 0.6 | 0.6 | 0.6 | 0.6 | 1.0 | 1 | 1.31 | | 28.57 | 30.99 | 32.30 | 15.91 | 8.80 | 4.94 | - | - |
|  | 0.6 | 0.6 | 0.6 | 0.6 | 1.0 | 2 | 1.31 | | 24.55 | 28.66 | 30.98 | 27.77 | 15.65 | 8.84 | - | - |
|  | 0.6 | 0.6 | 0.6 | 0.6 | 1.0 | 3 | 1.31 | | 20.94 | 26.53 | 29.75 | 38.37 | 21.91 | 12.45 | - | - |
|  | 0.6 | 0.6 | 0.6 | 0.6 | 1.0 | 5 | 1.31 | | 13.40 | 22.20 | 27.17 | 60.58 | 34.67 | 20.04 | - | - |
|  | 0.6 | 0.6 | 0.6 | 0.6 | 1.0 | 10 | 1.31 | | 13.40 | 10.20 | 19.09 | 60.58 | 70.00 | 43.81 | 8.01 | - |
|  | 0.6 | 0.6 | 0.6 | 0.6 | 1.0 | 20 | 1.31 | | 13.40 | 10.20 | 9.07 | 60.58 | 70.00 | 73.32 | 8.01 | - |
|  | 0.6 | 0.6 | 0.6 | 0.6 | 2.0 | 1 | 1.57 | | 28.57 | 30.99 | 32.30 | 15.91 | 8.80 | 4.94 | - | - |
|  | 0.6 | 0.6 | 0.6 | 0.6 | 2.0 | 2 | 1.57 | | 28.57 | 30.99 | 32.30 | 15.91 | 8.80 | 4.94 | - | - |
|  | 0.6 | 0.6 | 0.6 | 0.6 | 2.0 | 3 | 1.57 | | 23.35 | 28.07 | 30.64 | 31.29 | 17.41 | 9.83 | - | - |
|  | 0.6 | 0.6 | 0.6 | 0.6 | 2.0 | 5 | 1.57 | | 16.77 | 24.86 | 28.78 | 50.66 | 26.84 | 15.29 | - | - |
|  | 0.6 | 0.6 | 0.6 | 0.6 | 2.0 | 10 | 1.57 | | 16.77 | 16.93 | 24.09 | 50.66 | 50.17 | 29.12 | - | - |
|  | 0.6 | 0.6 | 0.6 | 0.6 | 2.0 | 20 | 1.57 | | 16.77 | 16.93 | 13.08 | 50.66 | 50.17 | 61.50 | - | - |
|  | 0.8 | 0.8 | 0.8 | 0.8 | 0.5 | 1 | 0.91 | | 23.18 | 27.78 | 30.46 | 31.80 | 18.25 | 10.36 | 0.50 | - |
|  | 0.8 | 0.8 | 0.8 | 0.8 | 0.5 | 2 | 0.91 | | 18.81 | 25.05 | 28.87 | 44.64 | 26.27 | 15.04 | 0.50 | - |
|  | 0.8 | 0.8 | 0.8 | 0.8 | 0.5 | 3 | 0.91 | | 14.77 | 22.33 | 27.24 | 56.53 | 34.29 | 19.83 | 0.50 | - |
|  | 0.8 | 0.8 | 0.8 | 0.8 | 0.5 | 5 | 0.91 | | 6.48 | 16.57 | 23.51 | 80.93 | 51.24 | 30.82 | 0.50 | - |
| Whole^#^ | 0.8 | 0.8 | 0.8 | 0.8 | 0.5 | 10 | 0.91 | | 6.48 | 3.48 | 12.42 | 80.93 | 89.76 | 63.44 | 0.50 | 9.48 |
|  | 0.8 | 0.8 | 0.8 | 0.8 | 0.5 | 20 | 0.91 | | 6.48 | 3.48 | 2.16 | 80.93 | 89.76 | 93.65 | 0.50 | 9.48 |
|  | 0.8 | 0.8 | 0.8 | 0.8 | 1.0 | 1 | 1.13 | | 26.55 | 29.81 | 31.63 | 21.87 | 12.28 | 6.93 | - | - |
|  | 0.8 | 0.8 | 0.8 | 0.8 | 1.0 | 2 | 1.13 | | 22.19 | 27.20 | 30.13 | 34.71 | 19.95 | 11.33 | - | - |
|  | 0.8 | 0.8 | 0.8 | 0.8 | 1.0 | 3 | 1.13 | | 18.42 | 24.87 | 28.77 | 45.80 | 26.81 | 15.34 | 2.01 | - |
|  | 0.8 | 0.8 | 0.8 | 0.8 | 1.0 | 5 | 1.13 | | 10.76 | 20.03 | 25.81 | 68.33 | 41.06 | 24.05 | 2.01 | - |
|  | 0.8 | 0.8 | 0.8 | 0.8 | 1.0 | 10 | 1.13 | | 10.76 | 7.78 | 16.68 | 68.33 | 77.11 | 50.92 | 2.01 | - |
|  | 0.8 | 0.8 | 0.8 | 0.8 | 1.0 | 20 | 1.13 | | 10.76 | 7.78 | 6.65 | 68.33 | 77.11 | 80.42 | 2.01 | - |
|  | 0.8 | 0.8 | 0.8 | 0.8 | 2.0 | 1 | 1.38 | | 26.55 | 29.81 | 31.63 | 21.87 | 12.28 | 6.93 | - | - |
|  | 0.8 | 0.8 | 0.8 | 0.8 | 2.0 | 2 | 1.38 | | 26.55 | 29.81 | 31.63 | 21.87 | 12.28 | 6.93 | - | - |
|  | 0.8 | 0.8 | 0.8 | 0.8 | 2.0 | 3 | 1.38 | | 20.42 | 26.23 | 29.57 | 39.91 | 22.81 | 12.98 | - | - |
|  | 0.8 | 0.8 | 0.8 | 0.8 | 2.0 | 5 | 1.38 | | 13.20 | 22.41 | 27.30 | 61.14 | 34.06 | 19.67 | 4.01 | - |
|  | 0.8 | 0.8 | 0.8 | 0.8 | 2.0 | 10 | 1.38 | | 13.20 | 13.64 | 21.65 | 61.14 | 59.85 | 36.29 | 4.01 | - |
|  | 0.8 | 0.8 | 0.8 | 0.8 | 2.0 | 20 | 1.38 | | 13.20 | 13.64 | 9.88 | 61.14 | 59.85 | 70.94 | 4.01 | - |
|  | 1.0 | 1.0 | 1.0 | 1.0 | 0.5 | 1 | 0.74 | | 21.85 | 26.94 | 29.97 | 35.70 | 20.71 | 11.80 | 0.01 | 0.50 |
|  | 1.0 | 1.0 | 1.0 | 1.0 | 0.5 | 2 | 0.74 | | 17.72 | 24.32 | 28.44 | 47.86 | 28.44 | 16.32 | 0.01 | 0.50 |
|  | 1.0 | 1.0 | 1.0 | 1.0 | 0.5 | 3 | 0.74 | | 13.60 | 21.41 | 26.68 | 59.98 | 37.00 | 21.50 | 0.01 | 0.50 |
|  | 1.0 | 1.0 | 1.0 | 1.0 | 0.5 | 5 | 0.74 | | 5.30 | 15.30 | 22.60 | 84.42 | 54.96 | 33.49 | 0.01 | 0.50 |
|  | 1.0 | 1.0 | 1.0 | 1.0 | 0.5 | 10 | 0.74 | | 5.30 | 2.19 | 10.60 | 84.42 | 93.56 | 68.80 | 0.01 | 0.50 |
|  | 1.0 | 1.0 | 1.0 | 1.0 | 0.5 | 20 | 0.74 | | 5.30 | 2.19 | 0.96 | 84.42 | 93.56 | 97.19 | 0.01 | 0.50 |
|  | 1.0 | 1.0 | 1.0 | 1.0 | 1.0 | 1 | 0.93 | | 24.38 | 28.49 | 30.87 | 28.25 | 16.16 | 9.17 | 0.01 | - |
|  | 1.0 | 1.0 | 1.0 | 1.0 | 1.0 | 2 | 0.93 | | 20.38 | 26.04 | 29.45 | 40.04 | 23.36 | 13.33 | 0.01 | - |
|  | 1.0 | 1.0 | 1.0 | 1.0 | 1.0 | 3 | 0.93 | | 16.58 | 23.60 | 28.01 | 51.21 | 30.56 | 17.58 | 0.01 | - |
|  | 1.0 | 1.0 | 1.0 | 1.0 | 1.0 | 5 | 0.93 | | 8.99 | 18.35 | 24.71 | 73.56 | 46.01 | 27.27 | 0.01 | 3.01 |
|  | 1.0 | 1.0 | 1.0 | 1.0 | 1.0 | 10 | 0.93 | | 8.99 | 6.02 | 14.75 | 73.56 | 82.29 | 56.60 | 0.01 | 3.01 |
|  | 1.0 | 1.0 | 1.0 | 1.0 | 1.0 | 20 | 0.93 | | 8.99 | 6.02 | 4.69 | 73.56 | 82.29 | 86.19 | 0.01 | 3.01 |
|  | 1.0 | 1.0 | 1.0 | 1.0 | 2.0 | 1 | 1.15 | | 24.38 | 28.49 | 30.87 | 28.25 | 16.16 | 9.17 | 0.01 | - |
|  | 1.0 | 1.0 | 1.0 | 1.0 | 2.0 | 2 | 1.15 | | 24.38 | 28.49 | 30.87 | 28.25 | 16.16 | 9.17 | 0.01 | - |
|  | 1.0 | 1.0 | 1.0 | 1.0 | 2.0 | 3 | 1.15 | | 17.88 | 24.54 | 28.57 | 47.37 | 27.77 | 15.92 | 0.01 | - |
|  | 1.0 | 1.0 | 1.0 | 1.0 | 2.0 | 5 | 1.15 | | 10.49 | 20.24 | 25.94 | 69.13 | 40.43 | 23.65 | 0.01 | - |
|  | 1.0 | 1.0 | 1.0 | 1.0 | 2.0 | 10 | 1.15 | | 10.49 | 11.15 | 19.53 | 69.13 | 67.19 | 42.52 | 0.01 | 8.01 |
|  | 1.0 | 1.0 | 1.0 | 1.0 | 2.0 | 20 | 1.15 | | 10.49 | 11.15 | 7.58 | 69.13 | 67.19 | 77.69 | 0.01 | 8.01 |
| At-risk^##^ | 0.0 | 0.4 | 0.4 | 0.4 | 0.5 | 1 | 1.34 | | 31.41 | 32.86 | 33.43 | 7.56 | 3.29 | 1.63 | - | - |
|  | 0.0 | 0.4 | 0.4 | 0.4 | 0.5 | 2 | 1.34 | | 29.29 | 31.94 | 32.98 | 13.80 | 6.00 | 2.96 | - | - |
|  | 0.0 | 0.4 | 0.4 | 0.4 | 0.5 | 3 | 1.34 | | 27.04 | 31.00 | 32.51 | 20.42 | 8.78 | 4.32 | - | - |
|  | 0.0 | 0.4 | 0.4 | 0.4 | 0.5 | 5 | 1.34 | | 20.56 | 28.75 | 31.42 | 39.49 | 15.40 | 7.53 | - | - |
|  | 0.0 | 0.4 | 0.4 | 0.4 | 0.5 | 10 | 1.34 | | 20.56 | 18.16 | 26.89 | 39.49 | 46.56 | 20.86 | - | - |
|  | 0.0 | 0.4 | 0.4 | 0.4 | 0.5 | 20 | 1.34 | | 20.56 | 18.16 | 16.71 | 39.49 | 46.56 | 50.81 | - | - |
|  | 0.0 | 0.4 | 0.4 | 0.4 | 1.0 | 1 | 1.57 | | 32.59 | 33.38 | 33.68 | 4.11 | 1.78 | 0.88 | - | - |
|  | 0.0 | 0.4 | 0.4 | 0.4 | 1.0 | 2 | 1.57 | | 31.19 | 32.78 | 33.39 | 8.21 | 3.53 | 1.74 | - | - |
|  | 0.0 | 0.4 | 0.4 | 0.4 | 1.0 | 3 | 1.57 | | 29.63 | 32.15 | 33.08 | 12.80 | 5.40 | 2.64 | - | - |
|  | 0.0 | 0.4 | 0.4 | 0.4 | 1.0 | 5 | 1.57 | | 25.08 | 30.61 | 32.36 | 26.18 | 9.92 | 4.79 | - | - |
| At-risk^##^ | 0.0 | 0.4 | 0.4 | 0.4 | 1.0 | 10 | 1.57 | | 25.08 | 23.05 | 29.32 | 26.18 | 32.16 | 13.72 | - | - |
|  | 0.0 | 0.4 | 0.4 | 0.4 | 1.0 | 20 | 1.57 | | 25.08 | 23.05 | 21.93 | 26.18 | 32.16 | 35.46 | - | - |
|  | 0.0 | 0.4 | 0.4 | 0.4 | 2.0 | 1 | 1.82 | | 32.59 | 33.38 | 33.68 | 4.11 | 1.78 | 0.88 | - | - |
|  | 0.0 | 0.4 | 0.4 | 0.4 | 2.0 | 2 | 1.82 | | 32.59 | 33.38 | 33.68 | 4.11 | 1.78 | 0.88 | - | - |
|  | 0.0 | 0.4 | 0.4 | 0.4 | 2.0 | 3 | 1.82 | | 30.68 | 32.62 | 33.32 | 9.71 | 4.00 | 1.94 | - | - |
|  | 0.0 | 0.4 | 0.4 | 0.4 | 2.0 | 5 | 1.82 | | 27.21 | 31.61 | 32.86 | 19.92 | 6.97 | 3.31 | - | - |
|  | 0.0 | 0.4 | 0.4 | 0.4 | 2.0 | 10 | 1.82 | | 27.21 | 27.88 | 31.39 | 19.92 | 17.96 | 7.63 | - | - |
|  | 0.0 | 0.4 | 0.4 | 0.4 | 2.0 | 20 | 1.82 | | 27.21 | 27.88 | 25.23 | 19.92 | 17.96 | 25.74 | - | - |
|  | 0.0 | 0.6 | 0.6 | 0.6 | 0.5 | 1 | 1.18 | | 30.36 | 32.38 | 33.19 | 10.67 | 4.70 | 2.33 | - | - |
|  | 0.0 | 0.6 | 0.6 | 0.6 | 0.5 | 2 | 1.18 | | 27.96 | 31.31 | 32.66 | 17.71 | 7.85 | 3.90 | - | - |
|  | 0.0 | 0.6 | 0.6 | 0.6 | 0.5 | 3 | 1.18 | | 25.43 | 30.21 | 32.11 | 25.17 | 11.10 | 5.51 | - | - |
|  | 0.0 | 0.6 | 0.6 | 0.6 | 0.5 | 5 | 1.18 | | 18.25 | 27.53 | 30.78 | 46.28 | 19.00 | 9.41 | - | - |
|  | 0.0 | 0.6 | 0.6 | 0.6 | 0.5 | 10 | 1.18 | | 18.25 | 15.66 | 25.31 | 46.28 | 53.92 | 25.53 | - | - |
|  | 0.0 | 0.6 | 0.6 | 0.6 | 0.5 | 20 | 1.18 | | 18.25 | 15.66 | 14.01 | 46.28 | 53.92 | 58.76 | - | - |
|  | 0.0 | 0.6 | 0.6 | 0.6 | 1.0 | 1 | 1.38 | | 31.83 | 33.04 | 33.52 | 6.33 | 2.77 | 1.37 | - | - |
|  | 0.0 | 0.6 | 0.6 | 0.6 | 1.0 | 2 | 1.38 | | 29.97 | 32.23 | 33.12 | 11.79 | 5.15 | 2.54 | - | - |
|  | 0.0 | 0.6 | 0.6 | 0.6 | 1.0 | 3 | 1.38 | | 28.00 | 31.40 | 32.71 | 17.61 | 7.61 | 3.75 | - | - |
|  | 0.0 | 0.6 | 0.6 | 0.6 | 1.0 | 5 | 1.38 | | 22.46 | 29.40 | 31.74 | 33.92 | 13.48 | 6.59 | - | - |
|  | 0.0 | 0.6 | 0.6 | 0.6 | 1.0 | 10 | 1.38 | | 22.46 | 20.11 | 27.74 | 33.92 | 40.83 | 18.37 | - | - |
|  | 0.0 | 0.6 | 0.6 | 0.6 | 1.0 | 20 | 1.38 | | 22.46 | 20.11 | 18.73 | 33.92 | 40.83 | 44.89 | - | - |
|  | 0.0 | 0.6 | 0.6 | 0.6 | 2.0 | 1 | 1.62 | | 31.83 | 33.04 | 33.52 | 6.33 | 2.77 | 1.37 | - | - |
|  | 0.0 | 0.6 | 0.6 | 0.6 | 2.0 | 2 | 1.62 | | 31.83 | 33.04 | 33.52 | 6.33 | 2.77 | 1.37 | - | - |
|  | 0.0 | 0.6 | 0.6 | 0.6 | 2.0 | 3 | 1.62 | | 29.15 | 31.95 | 32.99 | 14.22 | 5.98 | 2.92 | - | - |
|  | 0.0 | 0.6 | 0.6 | 0.6 | 2.0 | 5 | 1.62 | | 24.49 | 30.51 | 32.31 | 27.93 | 10.20 | 4.91 | - | - |
|  | 0.0 | 0.6 | 0.6 | 0.6 | 2.0 | 10 | 1.62 | | 24.49 | 25.41 | 30.21 | 27.93 | 25.23 | 11.10 | - | - |
|  | 0.0 | 0.6 | 0.6 | 0.6 | 2.0 | 20 | 1.62 | | 24.49 | 25.41 | 21.94 | 27.93 | 25.23 | 35.45 | - | - |
|  | 0.0 | 0.8 | 0.8 | 0.8 | 0.5 | 1 | 1.06 | | 29.51 | 31.99 | 32.99 | 13.15 | 5.86 | 2.92 | - | - |
|  | 0.0 | 0.8 | 0.8 | 0.8 | 0.5 | 2 | 1.06 | | 27.09 | 30.88 | 32.44 | 20.29 | 9.11 | 4.54 | - | - |
|  | 0.0 | 0.8 | 0.8 | 0.8 | 0.5 | 3 | 1.06 | | 24.37 | 29.67 | 31.83 | 28.28 | 12.69 | 6.34 | - | - |
|  | 0.0 | 0.8 | 0.8 | 0.8 | 0.5 | 5 | 1.06 | | 16.82 | 26.69 | 30.33 | 50.50 | 21.47 | 10.73 | - | - |
|  | 0.0 | 0.8 | 0.8 | 0.8 | 0.5 | 10 | 1.06 | | 16.82 | 14.09 | 24.20 | 50.50 | 58.55 | 28.80 | - | - |
|  | 0.0 | 0.8 | 0.8 | 0.8 | 0.5 | 20 | 1.06 | | 16.82 | 14.09 | 12.29 | 50.50 | 58.55 | 63.84 | - | - |
|  | 0.0 | 0.8 | 0.8 | 0.8 | 1.0 | 1 | 1.23 | | 31.03 | 32.68 | 33.34 | 8.68 | 3.83 | 1.90 | - | - |
|  | 0.0 | 0.8 | 0.8 | 0.8 | 1.0 | 2 | 1.23 | | 28.92 | 31.74 | 32.87 | 14.89 | 6.60 | 3.27 | - | - |
|  | 0.0 | 0.8 | 0.8 | 0.8 | 1.0 | 3 | 1.23 | | 26.70 | 30.77 | 32.39 | 21.43 | 9.44 | 4.68 | - | - |
|  | 0.0 | 0.8 | 0.8 | 0.8 | 1.0 | 5 | 1.23 | | 20.56 | 28.44 | 31.24 | 39.49 | 16.32 | 8.05 | - | - |
|  | 0.0 | 0.8 | 0.8 | 0.8 | 1.0 | 10 | 1.23 | | 20.56 | 18.04 | 26.50 | 39.49 | 46.92 | 22.03 | - | - |
|  | 0.0 | 0.8 | 0.8 | 0.8 | 1.0 | 20 | 1.23 | | 20.56 | 18.04 | 16.49 | 39.49 | 46.92 | 51.48 | - | - |
|  | 0.0 | 0.8 | 0.8 | 0.8 | 2.0 | 1 | 1.44 | | 31.03 | 32.68 | 33.34 | 8.68 | 3.83 | 1.90 | - | - |
|  | 0.0 | 0.8 | 0.8 | 0.8 | 2.0 | 2 | 1.44 | | 31.03 | 32.68 | 33.34 | 8.68 | 3.83 | 1.90 | - | - |
|  | 0.0 | 0.8 | 0.8 | 0.8 | 2.0 | 3 | 1.44 | | 27.72 | 31.30 | 32.66 | 18.42 | 7.90 | 3.89 | - | - |
|  | 0.0 | 0.8 | 0.8 | 0.8 | 2.0 | 5 | 1.44 | | 22.18 | 29.49 | 31.80 | 34.73 | 13.22 | 6.42 | - | - |
| At-risk^##^ | 0.0 | 0.8 | 0.8 | 0.8 | 2.0 | 10 | 1.44 | | 22.18 | 23.28 | 29.12 | 34.73 | 31.48 | 14.31 | - | - |
|  | 0.0 | 0.8 | 0.8 | 0.8 | 2.0 | 20 | 1.44 | | 22.18 | 23.28 | 19.22 | 34.73 | 31.48 | 43.45 | - | - |
|  | 0.0 | 1.0 | 1.0 | 1.0 | 0.5 | 1 | 0.94 | | 28.92 | 31.71 | 32.85 | 14.90 | 6.70 | 3.34 | - | - |
|  | 0.0 | 1.0 | 1.0 | 1.0 | 0.5 | 2 | 0.94 | | 26.48 | 30.58 | 32.28 | 22.09 | 10.01 | 5.01 | - | - |
|  | 0.0 | 1.0 | 1.0 | 1.0 | 0.5 | 3 | 0.94 | | 23.62 | 29.27 | 31.62 | 30.50 | 13.87 | 6.95 | - | - |
|  | 0.0 | 1.0 | 1.0 | 1.0 | 0.5 | 5 | 0.94 | | 15.83 | 26.06 | 30.00 | 53.42 | 23.31 | 11.73 | - | - |
|  | 0.0 | 1.0 | 1.0 | 1.0 | 0.5 | 10 | 0.94 | | 15.83 | 12.96 | 23.35 | 53.42 | 61.85 | 31.29 | - | - |
|  | 0.0 | 1.0 | 1.0 | 1.0 | 0.5 | 20 | 0.94 | | 15.83 | 12.96 | 11.02 | 53.42 | 61.85 | 67.56 | 18.45 | - |
|  | 0.0 | 1.0 | 1.0 | 1.0 | 1.0 | 1 | 1.07 | | 30.19 | 32.29 | 33.14 | 11.17 | 4.97 | 2.47 | - | - |
|  | 0.0 | 1.0 | 1.0 | 1.0 | 1.0 | 2 | 1.07 | | 28.07 | 31.33 | 32.66 | 17.40 | 7.80 | 3.89 | - | - |
|  | 0.0 | 1.0 | 1.0 | 1.0 | 1.0 | 3 | 1.07 | | 25.68 | 30.27 | 32.13 | 24.42 | 10.92 | 5.44 | - | - |
|  | 0.0 | 1.0 | 1.0 | 1.0 | 1.0 | 5 | 1.07 | | 19.16 | 27.66 | 30.84 | 43.62 | 18.60 | 9.26 | - | - |
|  | 0.0 | 1.0 | 1.0 | 1.0 | 1.0 | 10 | 1.07 | | 19.16 | 16.52 | 25.49 | 43.62 | 51.37 | 24.98 | - | - |
|  | 0.0 | 1.0 | 1.0 | 1.0 | 1.0 | 20 | 1.07 | | 19.16 | 16.52 | 14.85 | 43.62 | 51.37 | 56.30 | - | - |
|  | 0.0 | 1.0 | 1.0 | 1.0 | 2.0 | 1 | 1.24 | | 30.19 | 32.29 | 33.14 | 11.17 | 4.97 | 2.47 | - | - |
|  | 0.0 | 1.0 | 1.0 | 1.0 | 2.0 | 2 | 1.24 | | 30.19 | 32.29 | 33.14 | 11.17 | 4.97 | 2.47 | - | - |
|  | 0.0 | 1.0 | 1.0 | 1.0 | 2.0 | 3 | 1.24 | | 26.43 | 30.68 | 32.35 | 22.23 | 9.72 | 4.81 | - | - |
|  | 0.0 | 1.0 | 1.0 | 1.0 | 2.0 | 5 | 1.24 | | 20.23 | 28.55 | 31.32 | 40.46 | 15.98 | 7.84 | - | - |
|  | 0.0 | 1.0 | 1.0 | 1.0 | 2.0 | 10 | 1.24 | | 20.23 | 21.46 | 28.12 | 40.46 | 36.85 | 17.24 | - | - |
|  | 0.0 | 1.0 | 1.0 | 1.0 | 2.0 | 20 | 1.24 | | 20.23 | 21.46 | 16.99 | 40.46 | 36.85 | 50.00 | - | - |
|  | 0.0 | 0.0 | 0.0 | 1.0 | 0.5 | 1 | 1.52 | | 33.94 | 33.96 | 33.97 | 0.13 | 0.07 | 0.04 | - | - |
|  | 0.0 | 0.0 | 0.0 | 1.0 | 0.5 | 2 | 1.52 | | 33.89 | 33.93 | 33.95 | 0.28 | 0.15 | 0.08 | - | - |
|  | 0.0 | 0.0 | 0.0 | 1.0 | 0.5 | 3 | 1.52 | | 33.84 | 33.90 | 33.94 | 0.42 | 0.25 | 0.13 | - | - |
|  | 0.0 | 0.0 | 0.0 | 1.0 | 0.5 | 5 | 1.52 | | 33.72 | 33.82 | 33.90 | 0.76 | 0.47 | 0.25 | - | - |
|  | 0.0 | 0.0 | 0.0 | 1.0 | 0.5 | 10 | 1.52 | | 33.72 | 33.56 | 33.73 | 0.76 | 1.23 | 0.74 | - | - |
|  | 0.0 | 0.0 | 0.0 | 1.0 | 0.5 | 20 | 1.52 | | 33.72 | 33.56 | 33.48 | 0.76 | 1.23 | 1.48 | - | - |
|  | 0.0 | 0.0 | 0.0 | 1.0 | 1.0 | 1 | 1.56 | | 33.96 | 33.97 | 33.98 | 0.06 | 0.03 | 0.02 | - | - |
|  | 0.0 | 0.0 | 0.0 | 1.0 | 1.0 | 2 | 1.56 | | 33.94 | 33.96 | 33.97 | 0.14 | 0.07 | 0.04 | - | - |
|  | 0.0 | 0.0 | 0.0 | 1.0 | 1.0 | 3 | 1.56 | | 33.91 | 33.94 | 33.96 | 0.21 | 0.12 | 0.06 | - | - |
|  | 0.0 | 0.0 | 0.0 | 1.0 | 1.0 | 5 | 1.56 | | 33.88 | 33.90 | 33.94 | 0.30 | 0.23 | 0.12 | - | - |
|  | 0.0 | 0.0 | 0.0 | 1.0 | 1.0 | 10 | 1.56 | | 33.88 | 33.80 | 33.86 | 0.30 | 0.53 | 0.35 | - | - |
|  | 0.0 | 0.0 | 0.0 | 1.0 | 1.0 | 20 | 1.56 | | 33.88 | 33.80 | 33.76 | 0.30 | 0.53 | 0.66 | - | - |
|  | 0.0 | 0.0 | 0.0 | 1.0 | 2.0 | 1 | 1.64 | | 33.96 | 33.97 | 33.98 | 0.06 | 0.03 | 0.02 | - | - |
|  | 0.0 | 0.0 | 0.0 | 1.0 | 2.0 | 2 | 1.64 | | 33.96 | 33.97 | 33.98 | 0.06 | 0.03 | 0.02 | - | - |
|  | 0.0 | 0.0 | 0.0 | 1.0 | 2.0 | 3 | 1.64 | | 33.94 | 33.95 | 33.97 | 0.14 | 0.08 | 0.04 | - | - |
|  | 0.0 | 0.0 | 0.0 | 1.0 | 2.0 | 5 | 1.64 | | 33.93 | 33.94 | 33.96 | 0.17 | 0.14 | 0.07 | - | - |
|  | 0.0 | 0.0 | 0.0 | 1.0 | 2.0 | 10 | 1.64 | | 33.93 | 33.89 | 33.93 | 0.17 | 0.27 | 0.16 | - | - |
|  | 0.0 | 0.0 | 0.0 | 1.0 | 2.0 | 20 | 1.64 | | 33.93 | 33.89 | 33.87 | 0.17 | 0.27 | 0.33 | - | - |

*The parameters were set to the best set of parameter estimates; each control strategy was simulated for 50 years. $C_{m,i} (i=1,2,3,4)$, $F$ and $D$ indicate the coverage in the $i$^th^ group of population, treatment times per year and duration years of intervention, respectively. $R_{c}$ is the control reproduction number, $P_{s5}$,$P_{s10}$ and $P_{s15}$ indicate the prevalence in 5, 10 and 15 years from the beginning of intervention, respectively. $r_{s5}$, $r_{s10}$ and $r_{s15}$ indicate the reduced rates in 5, 10 and 15 years, compared with the baseline prevalence, respectively. $Y_{5\%}$ and $Y_{1\%}$ indicate the years from the beginning of intervention to infection control and transmission control, respectively.

^#^Strategy targeted for whole population (green shade).

^##^Strategy targeted for at-risk population (blue shade).
