# Supplementary material for: Assessment of control strategies against Clonorchis sinensis infection based on a multi-group dynamic transmission model
Source: PLoS Negl Trop Dis. 2020 Mar 27;14(3):e0008152. doi: 10.1371/journal.pntd.0008152 (PMC7156112; doi:10.1371/journal.pntd.0008152)
Supplement: S4 Table — (DOCX) [file pntd.0008152.s009.docx]

**S4 Table. Results of simulations applied single IEC with different coverages*****.**

| Strategy | | | | | | |  | Effectiveness | | | | | | | | |
| --- | --- | --- | --- | --- | --- | --- | --- | --- | --- | --- | --- | --- | --- | --- | --- | --- |
| $C_{e1,1}$ | $C_{e1,2}$ | $C_{e1,3}$ | $C_{e1,4}$ | $C_{e2,2}$ | $C_{e2,3}$ | $C_{e2,4}$ |  | $R_{c}$ | $P_{s5}$ | $P_{s10}$ | $P_{s15}$ | $r_{s5}$ | $r_{s10}$ | $r_{s15}$ | $Y_{5\%}$ | $Y_{1\%}$ |
| 0.20 | 0.20 | 0.20 | 0.20 | 0.20 | 0.20 | 0.20 | 2.28 | | 31.69 | 30.67 | 30.09 | 6.74 | 9.76 | 11.44 | - | - |
| 0.40 | 0.40 | 0.40 | 0.40 | 0.40 | 0.40 | 0.40 | 2.08 | | 29.13 | 26.90 | 25.64 | 14.28 | 20.83 | 24.56 | - | - |
| 0.60 | 0.60 | 0.60 | 0.60 | 0.60 | 0.60 | 0.60 | 1.81 | | 26.20 | 22.53 | 20.40 | 22.89 | 33.69 | 39.95 | - | - |
| 0.80 | 0.80 | 0.80 | 0.80 | 0.80 | 0.80 | 0.80 | 1.44 | | 22.70 | 17.24 | 14.08 | 33.20 | 49.27 | 58.55 | - | - |
| 0.90 | 0.90 | 0.90 | 0.90 | 0.90 | 0.90 | 0.90 | 1.14 | | 20.48 | 13.92 | 10.20 | 39.73 | 59.04 | 69.97 | 33.87 | - |
| 0.94 | 0.94 | 0.94 | 0.94 | 0.94 | 0.94 | 0.94 | 0.96 | | 19.33 | 12.24 | 8.29 | 43.10 | 63.97 | 75.60 | 22.88 | - |
| 1.00 | 1.00 | 1.00 | 1.00 | 1.00 | 1.00 | 1.00 | 0.00 | | 16.68 | 8.19 | 4.02 | 50.92 | 75.91 | 88.18 | 13.47 | 24.78 |
| 0.20 | 0.20 | 0.20 | 0.20 | 0.00 | 0.00 | 0.00 | 2.45 | | 32.83 | 32.18 | 31.80 | 3.38 | 5.30 | 6.43 | - | - |
| 0.40 | 0.40 | 0.40 | 0.40 | 0.00 | 0.00 | 0.00 | 2.45 | | 31.67 | 30.37 | 29.60 | 6.79 | 10.64 | 12.89 | - | - |
| 0.60 | 0.60 | 0.60 | 0.60 | 0.00 | 0.00 | 0.00 | 2.44 | | 30.50 | 28.55 | 27.41 | 10.23 | 15.99 | 19.34 | - | - |
| 0.80 | 0.80 | 0.80 | 0.80 | 0.00 | 0.00 | 0.00 | 2.43 | | 29.33 | 26.73 | 25.24 | 13.70 | 21.35 | 25.73 | - | - |
| 0.90 | 0.90 | 0.90 | 0.90 | 0.00 | 0.00 | 0.00 | 2.43 | | 28.73 | 25.82 | 24.16 | 15.44 | 24.02 | 28.90 | - | - |
| 1.00 | 1.00 | 1.00 | 1.00 | 0.00 | 0.00 | 0.00 | 2.42 | | 28.14 | 24.92 | 23.10 | 17.19 | 26.68 | 32.03 | - | - |
| 0.00 | 0.00 | 0.00 | 0.00 | 0.00 | 0.00 | 1.00^#^ | 1.60 | | 33.05 | 32.88 | 32.80 | 2.74 | 3.24 | 3.49 | - | - |
| 0.00 | 0.00 | 0.00 | 0.00 | 0.00 | 1.00^##^ | 1.00^##^ | 1.30 | | 29.82 | 28.18 | 27.28 | 12.25 | 17.08 | 19.71 | - | - |
| 0.00 | 0.00 | 0.00 | 0.00 | 0.20 | 0.20 | 0.20 | 2.29 | | 32.85 | 32.48 | 32.30 | 3.34 | 4.41 | 4.94 | - | - |
| 0.00 | 0.00 | 0.00 | 0.00 | 0.40 | 0.40 | 0.40 | 2.10 | | 31.47 | 30.57 | 30.10 | 7.38 | 10.04 | 11.42 | - | - |
| 0.00 | 0.00 | 0.00 | 0.00 | 0.60 | 0.60 | 0.60 | 1.85 | | 29.76 | 28.04 | 27.08 | 12.41 | 17.48 | 20.30 | - | - |
| 0.00 | 0.00 | 0.00 | 0.00 | 0.80 | 0.80 | 0.80 | 1.52 | | 27.50 | 24.47 | 22.65 | 19.07 | 27.98 | 33.35 | - | - |
| 0.00 | 0.00 | 0.00 | 0.00 | 0.90 | 0.90 | 0.90 | 1.28 | | 25.91 | 21.89 | 19.35 | 23.76 | 35.59 | 43.05 | - | - |
| 0.00 | 0.00 | 0.00 | 0.00 | 1.00 | 1.00 | 1.00 | 0.88 | | 22.72 | 16.32 | 12.18 | 33.13 | 51.96 | 64.15 | 33.19 | - |

*The parameters were set to the best set of parameter estimates; each control strategy was simulated for 50 years. $C_{e1,i} (i=1,2,3,4)$ and $C_{e2,i} (i=2,3,4)$ indicate the improvement rate of hygiene habits and the proportion of population who stop eating raw fish (if no special comment is specified) in the $i$^th^ group by IEC, respectively. $R_{c}$ is the control reproduction number, $P_{s5}$,$P_{s10}$ and $P_{s15}$ indicate the prevalence in 5, 10 and 15 years from the beginning of intervention, respectively. $r_{s5}$, $r_{s10}$ and $r_{s15}$ indicate the reduced rates in 5, 10 and 15 years, compared with the baseline prevalence, respectively. $Y_{5\%}$ and $Y_{1\%}$ indicate the years from the beginning of intervention to infection control and transmission control, respectively.

^#^People who previous eat raw fish very often changed their behavior to eat often.

^##^People who previous eat raw fish very often or often changed their behavior to eat moderately.
