# Supplementary material for: Assessment of control strategies against Clonorchis sinensis infection based on a multi-group dynamic transmission model
Source: PLoS Negl Trop Dis. 2020 Mar 27;14(3):e0008152. doi: 10.1371/journal.pntd.0008152 (PMC7156112; doi:10.1371/journal.pntd.0008152)
Supplement: S5 Table — (DOCX) [file pntd.0008152.s010.docx]

**S5 Table. Results of simulations applied single environmental modification with different coverages*****.**

| Strategy |  | Effectiveness | | | | | | | | |
| --- | --- | --- | --- | --- | --- | --- | --- | --- | --- | --- |
| $C_{d}$ |  | $R_{c}$ | $P_{s5}$ | $P_{s10}$ | $P_{s15}$ | $r_{s5}$ | $r_{s10}$ | $r_{s15}$ | $Y_{5\%}$ | $Y_{1\%}$ |
| 0.2 | 2.28 | | 32.80 | 31.69 | 31.07 | 3.47 | 6.75 | 8.56 | - | - |
| 0.4 | 2.08 | | 31.47 | 28.93 | 27.46 | 7.39 | 14.88 | 19.18 | - | - |
| 0.6 | 1.81 | | 29.94 | 25.52 | 22.89 | 11.91 | 24.92 | 32.64 | - | - |
| 0.8 | 1.44 | | 28.13 | 21.14 | 16.92 | 17.21 | 37.78 | 50.20 | - | - |
| 0.9 | 1.14 | | 27.09 | 18.34 | 13.09 | 20.28 | 46.03 | 61.49 | 40.48 | - |
| 1.0 | 0.00 | | 25.89 | 14.27 | 7.15 | 23.80 | 58.00 | 78.95 | 17.53 | 28.85 |

*The parameters were set to the best set of estimates; each control strategy was simulated for 50 years. $C_{d}$ indicates the coverage of sanitation toilets. $R_{c}$ is the control reproduction number, $P_{s5}$,$P_{s10}$ and $P_{s15}$ indicate the prevalence in 5, 10 and 15 years from the beginning of intervention, respectively. $r_{s5}$, $r_{s10}$ and $r_{s15}$ indicate the reduced rates in 5, 10 and 15 years, compared with the baseline prevalence, respectively. $Y_{5\%}$ and $Y_{1\%}$ indicate the years from the beginning of intervention to infection control and transmission control, respectively.
