# Supplementary material for: Assessment of control strategies against Clonorchis sinensis infection based on a multi-group dynamic transmission model
Source: PLoS Negl Trop Dis. 2020 Mar 27;14(3):e0008152. doi: 10.1371/journal.pntd.0008152 (PMC7156112; doi:10.1371/journal.pntd.0008152)
Supplement: S6 Table — (DOCX) [file pntd.0008152.s011.docx]

**S6 Table. Results of simulations applied combined strategies with different coverages and durations targeted on whole population*****.**

| Strategy | | | | | |  | Effectiveness | | | | | | | | |
| --- | --- | --- | --- | --- | --- | --- | --- | --- | --- | --- | --- | --- | --- | --- | --- |
| Category | $C_{d}$ | $C_{e}$ | $C_{m}$ | $F$ | $D$ |  | $R_{c}$ | $P_{s5}$ | $P_{s10}$ | $P_{s15}$ | $r_{s5}$ | $r_{s10}$ | $r_{s15}$ | $Y_{5\%}$ | $Y_{1\%}$ |
| Chemotherapy + IEC | 0 | 0.2 | 0.4 | 1 | 2 | 1.41 | | 24.72 | 26.73 | 27.82 | 27.24 | 21.33 | 18.14 | - | - |
|  | 0 | 0.2 | 0.4 | 1 | 5 | 1.41 | | 15.41 | 21.51 | 24.68 | 54.64 | 36.71 | 27.38 | - | - |
|  | 0 | 0.2 | 0.4 | 1 | 10 | 1.41 | | 15.41 | 11.76 | 18.42 | 54.64 | 65.38 | 45.80 | - | - |
|  | 0 | 0.4 | 0.4 | 1 | 2 | 1.28 | | 21.77 | 22.65 | 23.08 | 35.95 | 33.35 | 32.08 | - | - |
|  | 0 | 0.4 | 0.4 | 1 | 5 | 1.28 | | 13.15 | 17.54 | 19.85 | 61.30 | 48.37 | 41.59 | - | - |
|  | 0 | 0.4 | 0.4 | 1 | 10 | 1.28 | | 13.15 | 9.31 | 14.20 | 61.30 | 72.60 | 58.21 | - | - |
|  | 0 | 0.6 | 0.4 | 1 | 2 | 1.12 | | 18.37 | 17.93 | 17.55 | 45.93 | 47.23 | 48.36 | - | - |
|  | 0 | 0.6 | 0.4 | 1 | 5 | 1.12 | | 10.58 | 13.16 | 14.36 | 68.86 | 61.28 | 57.74 | - | - |
|  | 0 | 0.6 | 0.4 | 1 | 10 | 1.12 | | 10.58 | 6.59 | 9.80 | 68.86 | 80.61 | 71.17 | 8.01 | - |
|  | 0 | 0.8 | 0.4 | 1 | 2 | 0.89 | | 14.32 | 12.34 | 11.02 | 57.86 | 63.70 | 67.58 | - | - |
|  | 0 | 0.8 | 0.4 | 1 | 5 | 0.89 | | 7.35 | 8.23 | 8.22 | 78.36 | 75.79 | 75.80 | - | - |
|  | 0 | 0.8 | 0.4 | 1 | 10 | 0.89 | | 7.35 | 3.37 | 4.92 | 78.36 | 90.09 | 85.54 | 5.01 | - |
|  | 0 | 1 | 0.4 | 1 | 2 | 0.00 | | 6.66 | 3.27 | 1.60 | 80.40 | 90.38 | 95.28 | 7.02 | 18.33 |
|  | 0 | 1 | 0.4 | 1 | 5 | 0.00 | | 1.68 | 0.83 | 0.41 | 95.05 | 97.57 | 98.81 | 3.01 | 8.65 |
|  | 0 | 1 | 0.4 | 1 | 10 | 0.00 | | 1.68 | 0.08 | 0.04 | 95.05 | 99.76 | 99.88 | 3.01 | 5.43 |
|  | 0 | 0.2 | 0.6 | 1 | 2 | 1.22 | | 21.82 | 24.95 | 26.76 | 35.78 | 26.57 | 21.27 | - | - |
|  | 0 | 0.2 | 0.6 | 1 | 5 | 1.22 | | 11.63 | 18.69 | 22.87 | 65.78 | 45.00 | 32.70 | - | - |
|  | 0 | 0.2 | 0.6 | 1 | 10 | 1.22 | | 11.63 | 8.37 | 15.42 | 65.78 | 75.36 | 54.61 | 6.01 | - |
|  | 0 | 0.4 | 0.6 | 1 | 2 | 1.10 | | 18.77 | 20.74 | 21.89 | 44.77 | 38.96 | 35.58 | - | - |
|  | 0 | 0.4 | 0.6 | 1 | 5 | 1.10 | | 9.71 | 14.89 | 18.02 | 71.44 | 56.19 | 46.96 | 4.01 | - |
|  | 0 | 0.4 | 0.6 | 1 | 10 | 1.10 | | 9.71 | 6.38 | 11.63 | 71.44 | 81.24 | 65.78 | 4.01 | - |
|  | 0 | 0.6 | 0.6 | 1 | 2 | 0.96 | | 15.30 | 15.92 | 16.23 | 54.98 | 53.15 | 52.24 | - | - |
|  | 0 | 0.6 | 0.6 | 1 | 5 | 0.96 | | 7.48 | 10.82 | 12.66 | 77.98 | 68.15 | 62.75 | 3.01 | - |
|  | 0 | 0.6 | 0.6 | 1 | 10 | 0.96 | | 7.48 | 4.10 | 7.73 | 77.98 | 87.94 | 77.25 | 3.01 | - |
|  | 0 | 0.8 | 0.6 | 1 | 2 | 0.77 | | 11.19 | 10.30 | 9.66 | 67.07 | 69.68 | 71.56 | - | - |
|  | 0 | 0.8 | 0.6 | 1 | 5 | 0.77 | | 4.62 | 6.30 | 6.82 | 86.39 | 81.48 | 79.94 | 2.01 | - |
|  | 0 | 0.8 | 0.6 | 1 | 10 | 0.77 | | 4.62 | 1.63 | 3.09 | 86.39 | 95.21 | 90.90 | 2.01 | 9.01 |
|  | 0 | 1 | 0.6 | 1 | 2 | 0.00 | | 3.35 | 1.64 | 0.81 | 90.15 | 95.16 | 97.63 | 2.19 | 13.49 |
|  | 0 | 1 | 0.6 | 1 | 5 | 0.00 | | 0.30 | 0.15 | 0.07 | 99.11 | 99.57 | 99.79 | 2.01 | 3.01 |
|  | 0 | 1 | 0.6 | 1 | 10 | 0.00 | | 0.30 | 0.00 | 0.00 | 99.11 | 99.99 | 100.00 | 2.01 | 3.01 |
|  | 0 | 0.2 | 0.8 | 1 | 2 | 1.05 | | 19.43 | 23.40 | 25.81 | 42.83 | 31.13 | 24.03 | 1.01 | - |
|  | 0 | 0.2 | 0.8 | 1 | 5 | 1.05 | | 9.28 | 16.66 | 21.50 | 72.70 | 50.98 | 36.72 | 1.01 | - |
|  | 0 | 0.2 | 0.8 | 1 | 10 | 1.05 | | 9.28 | 6.21 | 13.28 | 72.70 | 81.73 | 60.91 | 1.01 | - |
|  | 0 | 0.4 | 0.8 | 1 | 2 | 0.95 | | 16.36 | 19.11 | 20.84 | 51.86 | 43.76 | 38.66 | 1.01 | - |
|  | 0 | 0.4 | 0.8 | 1 | 5 | 0.95 | | 7.62 | 13.09 | 16.72 | 77.57 | 61.47 | 50.81 | 1.01 | - |
|  | 0 | 0.4 | 0.8 | 1 | 10 | 0.95 | | 7.62 | 4.44 | 9.83 | 77.57 | 86.94 | 71.08 | 1.01 | - |
|  | 0 | 0.6 | 0.8 | 1 | 2 | 0.83 | | 12.91 | 14.25 | 15.10 | 62.01 | 58.07 | 55.57 | 1.01 | - |
|  | 0 | 0.6 | 0.8 | 1 | 5 | 0.83 | | 5.65 | 9.36 | 11.53 | 83.36 | 72.46 | 66.06 | 1.01 | - |
|  | 0 | 0.6 | 0.8 | 1 | 10 | 0.83 | | 5.65 | 2.50 | 6.16 | 83.36 | 92.63 | 81.88 | 1.01 | 8.01 |
|  | 0 | 0.8 | 0.8 | 1 | 2 | 0.66 | | 8.86 | 8.69 | 8.54 | 73.92 | 74.42 | 74.85 | 1.01 | - |
| Chemotherapy + IEC | 0 | 0.8 | 0.8 | 1 | 5 | 0.66 | | 3.14 | 5.12 | 5.93 | 90.76 | 84.94 | 82.55 | 1.01 | - |
|  | 0 | 0.8 | 0.8 | 1 | 10 | 0.66 | | 3.14 | 0.78 | 1.84 | 90.76 | 97.69 | 94.60 | 1.01 | 5.01 |
|  | 0 | 1 | 0.8 | 1 | 2 | 0.00 | | 1.16 | 0.57 | 0.28 | 96.58 | 98.32 | 99.18 | 1.01 | 6.06 |
|  | 0 | 1 | 0.8 | 1 | 5 | 0.00 | | 0.02 | 0.01 | 0.01 | 99.94 | 99.97 | 99.98 | 1.01 | 2.01 |
|  | 0 | 1 | 0.8 | 1 | 10 | 0.00 | | 0.02 | 0.00 | 0.00 | 99.94 | 100.00 | 100.00 | 1.01 | 2.01 |
| Chemotherapy + environmental modification | 0.2 | 0 | 0.4 | 1 | 2 | 1.41 | | 26.13 | 27.89 | 28.85 | 23.09 | 17.92 | 15.10 | - | - |
|  | 0.2 | 0 | 0.4 | 1 | 5 | 1.41 | | 16.37 | 22.49 | 25.58 | 51.83 | 33.81 | 24.72 | - | - |
|  | 0.2 | 0 | 0.4 | 1 | 10 | 1.41 | | 16.37 | 12.25 | 19.02 | 51.83 | 63.94 | 44.02 | - | - |
|  | 0.4 | 0 | 0.4 | 1 | 2 | 1.28 | | 24.79 | 25.02 | 25.08 | 27.05 | 26.37 | 26.20 | - | - |
|  | 0.4 | 0 | 0.4 | 1 | 5 | 1.28 | | 15.15 | 19.53 | 21.59 | 55.43 | 42.54 | 36.48 | - | - |
|  | 0.4 | 0 | 0.4 | 1 | 10 | 1.28 | | 15.15 | 10.24 | 15.27 | 55.43 | 69.85 | 55.05 | - | - |
|  | 0.6 | 0 | 0.4 | 1 | 2 | 1.12 | | 23.29 | 21.59 | 20.41 | 31.46 | 36.46 | 39.94 | - | - |
|  | 0.6 | 0 | 0.4 | 1 | 5 | 1.12 | | 13.79 | 16.13 | 16.81 | 59.41 | 52.53 | 50.54 | - | - |
|  | 0.6 | 0 | 0.4 | 1 | 10 | 1.12 | | 13.79 | 7.96 | 11.16 | 59.41 | 76.58 | 67.15 | - | - |
|  | 0.8 | 0 | 0.4 | 1 | 2 | 0.89 | | 21.60 | 17.39 | 14.56 | 36.44 | 48.83 | 57.15 | - | - |
|  | 0.8 | 0 | 0.4 | 1 | 5 | 0.89 | | 12.25 | 12.20 | 11.18 | 63.96 | 64.10 | 67.11 | - | - |
|  | 0.8 | 0 | 0.4 | 1 | 10 | 0.89 | | 12.25 | 5.21 | 6.64 | 63.96 | 84.66 | 80.47 | 8.01 | - |
|  | 1 | 0 | 0.4 | 1 | 2 | 0.00 | | 19.62 | 11.28 | 5.69 | 42.25 | 66.80 | 83.26 | 15.92 | 27.24 |
|  | 1 | 0 | 0.4 | 1 | 5 | 0.00 | | 10.39 | 7.14 | 3.66 | 69.44 | 79.00 | 89.23 | 12.74 | 24.16 |
|  | 1 | 0 | 0.4 | 1 | 10 | 0.00 | | 10.39 | 1.78 | 1.09 | 69.44 | 94.77 | 96.78 | 6.01 | 15.68 |
|  | 0.2 | 0 | 0.6 | 1 | 2 | 1.22 | | 23.37 | 26.20 | 27.83 | 31.23 | 22.91 | 18.10 | - | - |
|  | 0.2 | 0 | 0.6 | 1 | 5 | 1.22 | | 12.46 | 19.65 | 23.76 | 63.33 | 42.18 | 30.09 | - | - |
|  | 0.2 | 0 | 0.6 | 1 | 10 | 1.22 | | 12.46 | 8.74 | 15.92 | 63.33 | 74.28 | 53.15 | 7.01 | - |
|  | 0.4 | 0 | 0.6 | 1 | 2 | 1.10 | | 22.09 | 23.32 | 24.01 | 35.00 | 31.36 | 29.34 | - | - |
|  | 0.4 | 0 | 0.6 | 1 | 5 | 1.10 | | 11.45 | 16.81 | 19.73 | 66.30 | 50.53 | 41.93 | - | - |
|  | 0.4 | 0 | 0.6 | 1 | 10 | 1.10 | | 11.45 | 7.11 | 12.50 | 66.30 | 79.06 | 63.20 | 6.01 | - |
|  | 0.6 | 0 | 0.6 | 1 | 2 | 0.96 | | 20.68 | 19.95 | 19.34 | 39.13 | 41.29 | 43.09 | - | - |
|  | 0.6 | 0 | 0.6 | 1 | 5 | 0.96 | | 10.35 | 13.67 | 15.06 | 69.55 | 59.76 | 55.67 | - | - |
|  | 0.6 | 0 | 0.6 | 1 | 10 | 0.96 | | 10.35 | 5.25 | 8.92 | 69.55 | 84.55 | 73.74 | 5.01 | - |
|  | 0.8 | 0 | 0.6 | 1 | 2 | 0.77 | | 19.13 | 15.91 | 13.60 | 43.70 | 53.19 | 59.97 | - | - |
|  | 0.8 | 0 | 0.6 | 1 | 5 | 0.77 | | 9.10 | 10.18 | 9.77 | 73.21 | 70.04 | 71.25 | - | - |
|  | 0.8 | 0 | 0.6 | 1 | 10 | 0.77 | | 9.10 | 3.11 | 4.92 | 73.21 | 90.84 | 85.52 | 5.01 | - |
|  | 1 | 0 | 0.6 | 1 | 2 | 0.00 | | 17.37 | 10.21 | 5.16 | 48.89 | 69.95 | 84.81 | 15.23 | 26.56 |
|  | 1 | 0 | 0.6 | 1 | 5 | 0.00 | | 7.63 | 5.93 | 3.07 | 77.54 | 82.54 | 90.97 | 4.01 | 22.93 |
|  | 1 | 0 | 0.6 | 1 | 10 | 0.00 | | 7.63 | 0.93 | 0.69 | 77.54 | 97.27 | 97.96 | 4.01 | 9.01 |
|  | 0.2 | 0 | 0.8 | 1 | 2 | 1.05 | | 21.08 | 24.73 | 26.94 | 37.95 | 27.22 | 20.73 | - | - |
|  | 0.2 | 0 | 0.8 | 1 | 5 | 1.05 | | 10.01 | 17.59 | 22.37 | 70.55 | 48.25 | 34.17 | 2.01 | - |
|  | 0.2 | 0 | 0.8 | 1 | 10 | 1.05 | | 10.01 | 6.53 | 13.72 | 70.55 | 80.78 | 59.63 | 2.01 | - |
|  | 0.4 | 0 | 0.8 | 1 | 2 | 0.95 | | 19.90 | 21.89 | 23.09 | 41.44 | 35.57 | 32.05 | - | - |
|  | 0.4 | 0 | 0.8 | 1 | 5 | 0.95 | | 9.20 | 14.94 | 18.39 | 72.94 | 56.04 | 45.87 | 2.01 | - |
|  | 0.4 | 0 | 0.8 | 1 | 10 | 0.95 | | 9.20 | 5.12 | 10.63 | 72.94 | 84.93 | 68.71 | 2.01 | - |
|  | 0.6 | 0 | 0.8 | 1 | 2 | 0.83 | | 18.63 | 18.61 | 18.45 | 45.19 | 45.24 | 45.71 | - | - |
| Chemotherapy + environmental modification | 0.6 | 0 | 0.8 | 1 | 5 | 0.83 | | 8.31 | 12.09 | 13.89 | 75.54 | 64.42 | 59.12 | 2.01 | - |
|  | 0.6 | 0 | 0.8 | 1 | 10 | 0.83 | | 8.31 | 3.56 | 7.42 | 75.54 | 89.54 | 78.16 | 2.01 | - |
|  | 0.8 | 0 | 0.8 | 1 | 2 | 0.66 | | 17.24 | 14.74 | 12.84 | 49.27 | 56.63 | 62.22 | - | - |
|  | 0.8 | 0 | 0.8 | 1 | 5 | 0.66 | | 7.32 | 8.99 | 8.91 | 78.46 | 73.54 | 73.77 | 2.01 | - |
|  | 0.8 | 0 | 0.8 | 1 | 10 | 0.66 | | 7.32 | 1.94 | 3.74 | 78.46 | 94.28 | 88.99 | 2.01 | 8.01 |
|  | 1 | 0 | 0.8 | 1 | 2 | 0.00 | | 15.70 | 9.42 | 4.77 | 53.79 | 72.28 | 85.95 | 14.67 | 26.02 |
|  | 1 | 0 | 0.8 | 1 | 5 | 0.00 | | 6.18 | 5.33 | 2.78 | 81.82 | 84.30 | 91.83 | 2.01 | 22.23 |
|  | 1 | 0 | 0.8 | 1 | 10 | 0.00 | | 6.18 | 0.58 | 0.53 | 81.82 | 98.29 | 98.44 | 2.01 | 7.01 |
| Chemotherapy + IEC + environmental modification | 0.4 | 0.2 | 0.4 | 1 | 2 | 1.19 | | 22.48 | 22.00 | 21.61 | 33.83 | 35.24 | 36.42 | - | - |
|  | 0.4 | 0.2 | 0.4 | 1 | 5 | 1.19 | | 13.42 | 16.68 | 18.12 | 60.52 | 50.91 | 46.67 | - | - |
|  | 0.4 | 0.2 | 0.4 | 1 | 10 | 1.19 | | 13.42 | 8.52 | 12.44 | 60.52 | 74.92 | 63.39 | - | - |
|  | 0.6 | 0.2 | 0.4 | 1 | 2 | 1.04 | | 21.19 | 19.03 | 17.53 | 37.64 | 44.00 | 48.40 | - | - |
|  | 0.6 | 0.2 | 0.4 | 1 | 5 | 1.04 | | 12.26 | 13.82 | 14.06 | 63.93 | 59.33 | 58.63 | - | - |
|  | 0.6 | 0.2 | 0.4 | 1 | 10 | 1.04 | | 12.26 | 6.57 | 9.10 | 63.93 | 80.67 | 73.22 | 9.01 | - |
|  | 0.8 | 0.2 | 0.4 | 1 | 2 | 0.83 | | 19.74 | 15.44 | 12.58 | 41.90 | 54.55 | 62.98 | - | - |
|  | 0.8 | 0.2 | 0.4 | 1 | 5 | 0.83 | | 10.93 | 10.55 | 9.39 | 67.83 | 68.94 | 72.37 | - | - |
|  | 0.8 | 0.2 | 0.4 | 1 | 10 | 0.83 | | 10.93 | 4.25 | 5.32 | 67.83 | 87.49 | 84.36 | 7.01 | - |
|  | 0.4 | 0.4 | 0.4 | 1 | 2 | 1.08 | | 19.91 | 18.66 | 17.75 | 41.41 | 45.09 | 47.76 | - | - |
|  | 0.4 | 0.4 | 0.4 | 1 | 5 | 1.08 | | 11.51 | 13.62 | 14.38 | 66.13 | 59.91 | 57.70 | - | - |
|  | 0.4 | 0.4 | 0.4 | 1 | 10 | 1.08 | | 11.51 | 6.66 | 9.55 | 66.13 | 80.41 | 71.89 | 9.01 | - |
|  | 0.6 | 0.4 | 0.4 | 1 | 2 | 0.95 | | 18.85 | 16.22 | 14.42 | 44.53 | 52.28 | 57.58 | - | - |
|  | 0.6 | 0.4 | 0.4 | 1 | 5 | 0.95 | | 10.55 | 11.36 | 11.16 | 68.96 | 66.56 | 67.16 | - | - |
|  | 0.6 | 0.4 | 0.4 | 1 | 10 | 0.95 | | 10.55 | 5.06 | 6.95 | 68.96 | 85.10 | 79.55 | 7.01 | - |
|  | 0.8 | 0.4 | 0.4 | 1 | 2 | 0.75 | | 17.67 | 13.31 | 10.44 | 48.00 | 60.85 | 69.28 | - | - |
|  | 0.8 | 0.4 | 0.4 | 1 | 5 | 0.75 | | 9.45 | 8.79 | 7.49 | 72.20 | 74.14 | 77.95 | 41.73 | - |
|  | 0.8 | 0.4 | 0.4 | 1 | 10 | 0.75 | | 9.45 | 3.23 | 3.88 | 72.20 | 90.49 | 88.58 | 6.01 | - |
|  | 0.4 | 0.6 | 0.4 | 1 | 2 | 0.95 | | 16.97 | 14.89 | 13.46 | 50.05 | 56.19 | 60.39 | - | - |
|  | 0.4 | 0.6 | 0.4 | 1 | 5 | 0.95 | | 9.30 | 10.32 | 10.36 | 72.62 | 69.62 | 69.51 | - | - |
|  | 0.4 | 0.6 | 0.4 | 1 | 10 | 0.95 | | 9.30 | 4.57 | 6.50 | 72.62 | 86.54 | 80.87 | 6.01 | - |
|  | 0.6 | 0.6 | 0.4 | 1 | 2 | 0.83 | | 16.18 | 13.07 | 11.00 | 52.38 | 61.55 | 67.62 | - | - |
|  | 0.6 | 0.6 | 0.4 | 1 | 5 | 0.83 | | 8.56 | 8.70 | 8.07 | 74.82 | 74.41 | 76.25 | - | - |
|  | 0.6 | 0.6 | 0.4 | 1 | 10 | 0.83 | | 8.56 | 3.41 | 4.54 | 74.82 | 89.95 | 86.64 | 6.01 | - |
|  | 0.8 | 0.6 | 0.4 | 1 | 2 | 0.66 | | 15.30 | 10.89 | 8.06 | 54.97 | 67.96 | 76.27 | 25.58 | - |
|  | 0.8 | 0.6 | 0.4 | 1 | 5 | 0.66 | | 7.70 | 6.81 | 5.43 | 77.33 | 79.95 | 84.03 | 17.08 | - |
|  | 0.8 | 0.6 | 0.4 | 1 | 10 | 0.66 | | 7.70 | 2.17 | 2.37 | 77.33 | 93.61 | 93.02 | 5.01 | - |
|  | 0.4 | 0.2 | 0.6 | 1 | 2 | 1.02 | | 19.70 | 20.23 | 20.46 | 42.02 | 40.46 | 39.79 | - | - |
|  | 0.4 | 0.2 | 0.6 | 1 | 5 | 1.02 | | 9.99 | 14.13 | 16.31 | 70.61 | 58.43 | 51.99 | - | - |
|  | 0.4 | 0.2 | 0.6 | 1 | 10 | 1.02 | | 9.99 | 5.71 | 10.04 | 70.61 | 83.20 | 70.46 | 5.01 | - |
|  | 0.6 | 0.2 | 0.6 | 1 | 2 | 0.90 | | 18.51 | 17.34 | 16.42 | 45.53 | 48.98 | 51.69 | - | - |
|  | 0.6 | 0.2 | 0.6 | 1 | 5 | 0.90 | | 9.04 | 11.55 | 12.42 | 73.39 | 66.01 | 63.44 | 4.01 | - |
|  | 0.6 | 0.2 | 0.6 | 1 | 10 | 0.90 | | 9.04 | 4.12 | 7.12 | 73.39 | 87.86 | 79.05 | 4.01 | - |
|  | 0.8 | 0.2 | 0.6 | 1 | 2 | 0.71 | | 17.20 | 13.92 | 11.60 | 49.39 | 59.04 | 65.87 | - | - |
| Chemotherapy + IEC + environmental modification | 0.8 | 0.2 | 0.6 | 1 | 5 | 0.71 | | 7.98 | 8.70 | 8.09 | 76.53 | 74.41 | 76.18 | 4.01 | - |
|  | 0.8 | 0.2 | 0.6 | 1 | 10 | 0.71 | | 7.98 | 2.40 | 3.72 | 76.53 | 92.93 | 89.06 | 4.01 | - |
|  | 0.4 | 0.4 | 0.6 | 1 | 2 | 0.93 | | 17.05 | 16.81 | 16.53 | 49.84 | 50.52 | 51.35 | - | - |
|  | 0.4 | 0.4 | 0.6 | 1 | 5 | 0.93 | | 8.35 | 11.31 | 12.69 | 75.42 | 66.72 | 62.65 | 4.01 | - |
|  | 0.4 | 0.4 | 0.6 | 1 | 10 | 0.93 | | 8.35 | 4.17 | 7.51 | 75.42 | 87.72 | 77.90 | 4.01 | - |
|  | 0.6 | 0.4 | 0.6 | 1 | 2 | 0.81 | | 16.09 | 14.47 | 13.26 | 52.66 | 57.41 | 60.97 | - | - |
|  | 0.6 | 0.4 | 0.6 | 1 | 5 | 0.81 | | 7.57 | 9.32 | 9.68 | 77.72 | 72.57 | 71.50 | 4.01 | - |
|  | 0.6 | 0.4 | 0.6 | 1 | 10 | 0.81 | | 7.57 | 2.94 | 5.14 | 77.72 | 91.35 | 84.88 | 4.01 | - |
|  | 0.8 | 0.4 | 0.6 | 1 | 2 | 0.65 | | 15.04 | 11.73 | 9.43 | 55.73 | 65.47 | 72.24 | 48.86 | - |
|  | 0.8 | 0.4 | 0.6 | 1 | 5 | 0.65 | | 6.69 | 7.10 | 6.32 | 80.30 | 79.10 | 81.40 | 3.01 | - |
|  | 0.8 | 0.4 | 0.6 | 1 | 10 | 0.65 | | 6.69 | 1.70 | 2.48 | 80.30 | 95.00 | 92.70 | 3.01 | - |
|  | 0.4 | 0.6 | 0.6 | 1 | 2 | 0.81 | | 14.03 | 12.99 | 12.20 | 58.70 | 61.76 | 64.11 | - | - |
|  | 0.4 | 0.6 | 0.6 | 1 | 5 | 0.81 | | 6.43 | 8.31 | 8.89 | 81.08 | 75.53 | 73.83 | 3.01 | - |
|  | 0.4 | 0.6 | 0.6 | 1 | 10 | 0.81 | | 6.43 | 2.53 | 4.66 | 81.08 | 92.54 | 86.28 | 3.01 | - |
|  | 0.6 | 0.6 | 0.6 | 1 | 2 | 0.71 | | 13.34 | 11.28 | 9.83 | 60.75 | 66.79 | 71.07 | - | - |
|  | 0.6 | 0.6 | 0.6 | 1 | 5 | 0.71 | | 5.83 | 6.90 | 6.78 | 82.85 | 79.69 | 80.04 | 3.01 | - |
|  | 0.6 | 0.6 | 0.6 | 1 | 10 | 0.71 | | 5.83 | 1.75 | 2.92 | 82.85 | 94.84 | 91.41 | 3.01 | 9.01 |
|  | 0.8 | 0.6 | 0.6 | 1 | 2 | 0.56 | | 12.58 | 9.28 | 7.04 | 62.97 | 72.70 | 79.29 | 22.92 | - |
|  | 0.8 | 0.6 | 0.6 | 1 | 5 | 0.56 | | 5.17 | 5.31 | 4.39 | 84.78 | 84.37 | 87.08 | 3.01 | - |
|  | 0.8 | 0.6 | 0.6 | 1 | 10 | 0.56 | | 5.17 | 1.03 | 1.33 | 84.78 | 96.98 | 96.08 | 3.01 | 8.01 |
|  | 0.4 | 0.2 | 0.8 | 1 | 2 | 0.88 | | 17.49 | 18.76 | 19.48 | 48.52 | 44.80 | 42.66 | 1.01 | - |
|  | 0.4 | 0.2 | 0.8 | 1 | 5 | 0.88 | | 7.94 | 12.45 | 15.07 | 76.62 | 63.36 | 55.65 | 1.01 | - |
|  | 0.4 | 0.2 | 0.8 | 1 | 10 | 0.88 | | 7.94 | 3.90 | 8.41 | 76.62 | 88.52 | 75.26 | 1.01 | - |
|  | 0.6 | 0.2 | 0.8 | 1 | 2 | 0.77 | | 16.43 | 15.97 | 15.50 | 51.65 | 53.00 | 54.39 | 1.01 | - |
|  | 0.6 | 0.2 | 0.8 | 1 | 5 | 0.77 | | 7.18 | 10.15 | 11.38 | 78.86 | 70.12 | 66.51 | 1.01 | - |
|  | 0.6 | 0.2 | 0.8 | 1 | 10 | 0.77 | | 7.18 | 2.64 | 5.71 | 78.86 | 92.23 | 83.19 | 1.01 | 9.01 |
|  | 0.8 | 0.2 | 0.8 | 1 | 2 | 0.61 | | 15.28 | 12.74 | 10.82 | 55.03 | 62.52 | 68.15 | 1.01 | - |
|  | 0.8 | 0.2 | 0.8 | 1 | 5 | 0.61 | | 6.34 | 7.65 | 7.34 | 81.35 | 77.50 | 78.41 | 1.01 | - |
|  | 0.8 | 0.2 | 0.8 | 1 | 10 | 0.61 | | 6.34 | 1.43 | 2.67 | 81.35 | 95.78 | 92.15 | 1.01 | 7.01 |
|  | 0.4 | 0.4 | 0.8 | 1 | 2 | 0.80 | | 14.83 | 15.31 | 15.51 | 56.37 | 54.95 | 54.35 | 1.01 | - |
|  | 0.4 | 0.4 | 0.8 | 1 | 5 | 0.80 | | 6.52 | 9.88 | 11.61 | 80.82 | 70.93 | 65.85 | 1.01 | - |
|  | 0.4 | 0.4 | 0.8 | 1 | 10 | 0.80 | | 6.52 | 2.63 | 6.02 | 80.82 | 92.26 | 82.28 | 1.01 | 8.01 |
|  | 0.6 | 0.4 | 0.8 | 1 | 2 | 0.70 | | 13.99 | 13.10 | 12.33 | 58.82 | 61.46 | 63.71 | 1.01 | - |
|  | 0.6 | 0.4 | 0.8 | 1 | 5 | 0.70 | | 5.89 | 8.13 | 8.79 | 82.65 | 76.09 | 74.13 | 1.01 | - |
|  | 0.6 | 0.4 | 0.8 | 1 | 10 | 0.70 | | 5.89 | 1.74 | 3.80 | 82.65 | 94.87 | 88.80 | 1.01 | 7.01 |
|  | 0.8 | 0.4 | 0.8 | 1 | 2 | 0.56 | | 13.10 | 10.54 | 8.65 | 61.44 | 68.98 | 74.54 | 1.01 | - |
|  | 0.8 | 0.4 | 0.8 | 1 | 5 | 0.56 | | 5.21 | 6.19 | 5.67 | 84.67 | 81.78 | 83.32 | 1.01 | - |
|  | 0.8 | 0.4 | 0.8 | 1 | 10 | 0.56 | | 5.21 | 0.96 | 1.66 | 84.67 | 97.17 | 95.12 | 1.01 | 7.01 |
|  | 0.4 | 0.6 | 0.8 | 1 | 2 | 0.70 | | 11.83 | 11.50 | 11.17 | 65.19 | 66.17 | 67.13 | 1.01 | - |
|  | 0.4 | 0.6 | 0.8 | 1 | 5 | 0.70 | | 4.81 | 7.14 | 8.01 | 85.83 | 78.99 | 76.44 | 1.01 | - |
|  | 0.4 | 0.6 | 0.8 | 1 | 10 | 0.70 | | 4.81 | 1.41 | 3.27 | 85.83 | 95.84 | 90.37 | 1.01 | 7.01 |
|  | 0.6 | 0.6 | 0.8 | 1 | 2 | 0.61 | | 11.25 | 9.91 | 8.90 | 66.91 | 70.83 | 73.80 | 1.01 | - |
| Chemotherapy + IEC + environmental modification | 0.6 | 0.6 | 0.8 | 1 | 5 | 0.61 | | 4.36 | 5.90 | 6.04 | 87.18 | 82.65 | 82.22 | 1.01 | - |
|  | 0.6 | 0.6 | 0.8 | 1 | 10 | 0.61 | | 4.36 | 0.94 | 1.88 | 87.18 | 97.23 | 94.46 | 1.01 | 6.01 |
|  | 0.8 | 0.6 | 0.8 | 1 | 2 | 0.49 | | 10.63 | 8.09 | 6.26 | 68.72 | 76.21 | 81.58 | 1.01 | - |
|  | 0.8 | 0.6 | 0.8 | 1 | 5 | 0.49 | | 3.87 | 4.53 | 3.84 | 88.61 | 86.67 | 88.70 | 1.01 | - |
|  | 0.8 | 0.6 | 0.8 | 1 | 10 | 0.49 | | 3.87 | 0.55 | 0.82 | 88.61 | 98.39 | 97.57 | 1.01 | 6.01 |
| IEC + environmental modification | 0.2 | 0.2 | 0 | 1 | 2 | 2.12 | | 30.65 | 28.60 | 27.43 | 9.80 | 15.85 | 19.29 | - | - |
|  | 0.4 | 0.2 | 0 | 1 | 2 | 1.93 | | 29.48 | 26.13 | 24.17 | 13.25 | 23.11 | 28.87 | - | - |
|  | 0.6 | 0.2 | 0 | 1 | 2 | 1.68 | | 28.14 | 23.13 | 20.14 | 17.19 | 31.92 | 40.73 | - | - |
|  | 0.8 | 0.2 | 0 | 1 | 2 | 1.34 | | 26.58 | 19.35 | 15.01 | 21.78 | 43.05 | 55.81 | - | - |
|  | 0.9 | 0.2 | 0 | 1 | 2 | 1.06 | | 25.68 | 16.93 | 11.74 | 24.44 | 50.17 | 65.45 | 31.96 | - |
|  | 1 | 0.2 | 0 | 1 | 2 | 0.00 | | 24.65 | 13.45 | 6.72 | 27.48 | 60.43 | 80.22 | 17.09 | 28.41 |
|  | 0.2 | 0.4 | 0 | 1 | 2 | 1.93 | | 28.25 | 25.12 | 23.30 | 16.86 | 26.09 | 31.45 | - | - |
|  | 0.4 | 0.4 | 0 | 1 | 2 | 1.75 | | 27.27 | 23.02 | 20.51 | 19.74 | 32.26 | 39.65 | - | - |
|  | 0.6 | 0.4 | 0 | 1 | 2 | 1.53 | | 26.16 | 20.51 | 17.14 | 23.02 | 39.63 | 49.55 | - | - |
|  | 0.8 | 0.4 | 0 | 1 | 2 | 1.21 | | 24.87 | 17.39 | 12.96 | 26.83 | 48.82 | 61.86 | - | - |
|  | 0.9 | 0.4 | 0 | 1 | 2 | 0.96 | | 24.12 | 15.38 | 10.27 | 29.03 | 54.73 | 69.78 | 26.24 | - |
|  | 1 | 0.4 | 0 | 1 | 2 | 0.00 | | 23.26 | 12.53 | 6.24 | 31.55 | 63.14 | 81.63 | 16.57 | 27.88 |
|  | 0.2 | 0.6 | 0 | 1 | 2 | 1.68 | | 25.53 | 21.12 | 18.54 | 24.88 | 37.84 | 45.44 | - | - |
|  | 0.4 | 0.6 | 0 | 1 | 2 | 1.53 | | 24.77 | 19.49 | 16.38 | 27.10 | 42.63 | 51.81 | - | - |
|  | 0.6 | 0.6 | 0 | 1 | 2 | 1.34 | | 23.92 | 17.57 | 13.83 | 29.61 | 48.28 | 59.29 | - | - |
|  | 0.8 | 0.6 | 0 | 1 | 2 | 1.06 | | 22.92 | 15.18 | 10.68 | 32.54 | 55.32 | 68.56 | 30.39 | - |
|  | 0.9 | 0.6 | 0 | 1 | 2 | 0.84 | | 22.35 | 13.62 | 8.62 | 34.24 | 59.91 | 74.63 | 21.74 | 47.24 |
|  | 1 | 0.6 | 0 | 1 | 2 | 0.00 | | 21.69 | 11.48 | 5.70 | 36.18 | 66.22 | 83.22 | 15.93 | 27.24 |
|  | 0.2 | 0.8 | 0 | 1 | 2 | 1.34 | | 22.27 | 16.34 | 12.91 | 34.46 | 51.92 | 62.02 | - | - |
|  | 0.4 | 0.8 | 0 | 1 | 2 | 1.21 | | 21.79 | 15.31 | 11.56 | 35.89 | 54.96 | 65.97 | - | - |
|  | 0.6 | 0.8 | 0 | 1 | 2 | 1.06 | | 21.23 | 14.08 | 9.98 | 37.51 | 58.57 | 70.63 | 29.08 | - |
|  | 0.8 | 0.8 | 0 | 1 | 2 | 0.84 | | 20.58 | 12.51 | 7.98 | 39.43 | 63.18 | 76.52 | 20.82 | 46.27 |
|  | 0.9 | 0.8 | 0 | 1 | 2 | 0.67 | | 20.20 | 11.50 | 6.68 | 40.54 | 66.16 | 80.33 | 17.79 | 34.47 |
|  | 1 | 0.8 | 0 | 1 | 2 | 0.00 | | 19.78 | 10.20 | 5.05 | 41.80 | 69.97 | 85.15 | 15.07 | 26.38 |
|  | 0.2 | 0.9 | 0 | 1 | 2 | 1.06 | | 20.19 | 13.33 | 9.45 | 40.58 | 60.77 | 72.18 | 27.79 | - |
|  | 0.4 | 0.9 | 0 | 1 | 2 | 0.96 | | 19.87 | 12.65 | 8.59 | 41.54 | 62.78 | 74.72 | 23.54 | - |
|  | 0.6 | 0.9 | 0 | 1 | 2 | 0.84 | | 19.49 | 11.82 | 7.56 | 42.64 | 65.21 | 77.74 | 20.16 | 45.49 |
|  | 0.8 | 0.9 | 0 | 1 | 2 | 0.67 | | 19.05 | 10.78 | 6.29 | 43.93 | 68.27 | 81.50 | 17.22 | 33.88 |
|  | 0.9 | 0.9 | 0 | 1 | 2 | 0.53 | | 18.81 | 10.14 | 5.52 | 44.66 | 70.16 | 83.77 | 15.82 | 29.58 |
|  | 1 | 0.9 | 0 | 1 | 2 | 0.00 | | 18.53 | 9.38 | 4.63 | 45.46 | 72.38 | 86.39 | 14.45 | 25.77 |
|  | 0.2 | 1 | 0 | 1 | 2 | 0.00 | | 16.68 | 8.19 | 4.02 | 50.92 | 75.91 | 88.18 | 13.47 | 24.78 |
|  | 0.4 | 1 | 0 | 1 | 2 | 0.00 | | 16.68 | 8.19 | 4.02 | 50.92 | 75.91 | 88.18 | 13.47 | 24.78 |
|  | 0.6 | 1 | 0 | 1 | 2 | 0.00 | | 16.68 | 8.19 | 4.02 | 50.92 | 75.91 | 88.18 | 13.47 | 24.78 |
|  | 0.8 | 1 | 0 | 1 | 2 | 0.00 | | 16.68 | 8.19 | 4.02 | 50.92 | 75.91 | 88.18 | 13.47 | 24.78 |
|  | 0.9 | 1 | 0 | 1 | 2 | 0.00 | | 16.68 | 8.19 | 4.02 | 50.92 | 75.91 | 88.18 | 13.47 | 24.78 |
|  | 1 | 1 | 0 | 1 | 2 | 0.00 | | 16.68 | 8.19 | 4.02 | 50.92 | 75.91 | 88.18 | 13.47 | 24.78 |

*The parameters were set to the best set of parameter estimates; each control strategy was simulated for 50 years. $C_{d}$, $C_{e}$, $C_{m}$ indicate the coverage of environmental modification, IEC (focus both on improvement of hygiene habits and changing people’s behavior of raw-fish-consumption) and chemotherapy, respectively. $R_{c}$ is the control reproduction number, $P_{s5}$,$P_{s10}$ and $P_{s15}$ indicate the prevalence in 5, 10 and 15 years from the beginning of intervention, respectively. $r_{s5}$, $r_{s10}$ and $r_{s15}$ indicate the reduced rates in 5, 10 and 15 years, compared with the baseline prevalence, respectively. $Y_{5\%}$ and $Y_{1\%}$ indicate the years from the beginning of intervention to infection control and transmission control, respectively. Strategies combined chemotherapy with IEC, combined chemotherapy with environmental modification, combined chemotherapy, IEC with environmental modification, and combined IEC with environmental modification are in green, blue, yellow and grey shades, respectively.
