# Supplementary material for: Assessment of control strategies against Clonorchis sinensis infection based on a multi-group dynamic transmission model
Source: PLoS Negl Trop Dis. 2020 Mar 27;14(3):e0008152. doi: 10.1371/journal.pntd.0008152 (PMC7156112; doi:10.1371/journal.pntd.0008152)
Supplement: S7 Table — (DOCX) [file pntd.0008152.s012.docx]

**S7 Table. Results of simulations applied combined strategies with different coverages and durations targeted on at-risk population with raw-fish-consumption behaviors*****.**

| Strategy | | | | | |  | Effectiveness | | | | | | | | |
| --- | --- | --- | --- | --- | --- | --- | --- | --- | --- | --- | --- | --- | --- | --- | --- |
| Category | $C_{d}$ | $C_{e}$ | $C_{m}$ | $F$ | $D$ |  | $R_{c}$ | $P_{s5}$ | $P_{s10}$ | $P_{s15}$ | $r_{s5}$ | $r_{s10}$ | $r_{s15}$ | $Y_{5\%}$ | $Y_{1\%}$ |
| Chemotherapy + IEC | 0 | 0.2 | 0.4 | 1 | 2 | 1.46 | | 28.51 | 29.22 | 29.36 | 16.10 | 14.00 | 13.61 | - | - |
|  | 0 | 0.2 | 0.4 | 1 | 5 | 1.46 | | 22.49 | 26.83 | 28.15 | 33.81 | 21.04 | 17.17 | - | - |
|  | 0 | 0.2 | 0.4 | 1 | 10 | 1.46 | | 22.49 | 19.61 | 24.85 | 33.81 | 42.28 | 26.87 | - | - |
|  | 0 | 0.4 | 0.4 | 1 | 2 | 1.32 | | 25.47 | 25.14 | 24.69 | 25.05 | 26.01 | 27.36 | - | - |
|  | 0 | 0.4 | 0.4 | 1 | 5 | 1.32 | | 19.69 | 22.55 | 23.28 | 42.05 | 33.63 | 31.49 | - | - |
|  | 0 | 0.4 | 0.4 | 1 | 10 | 1.32 | | 19.69 | 16.04 | 19.88 | 42.05 | 52.81 | 41.49 | - | - |
|  | 0 | 0.6 | 0.4 | 1 | 2 | 1.16 | | 21.95 | 20.35 | 19.16 | 35.42 | 40.10 | 43.62 | - | - |
|  | 0 | 0.6 | 0.4 | 1 | 5 | 1.16 | | 16.57 | 17.67 | 17.59 | 51.23 | 47.99 | 48.24 | - | - |
|  | 0 | 0.6 | 0.4 | 1 | 10 | 1.16 | | 16.57 | 12.24 | 14.47 | 51.23 | 63.98 | 57.42 | - | - |
|  | 0 | 0.8 | 0.4 | 1 | 2 | 0.92 | | 17.69 | 14.54 | 12.48 | 47.93 | 57.22 | 63.28 | - | - |
|  | 0 | 0.8 | 0.4 | 1 | 5 | 0.92 | | 12.77 | 12.04 | 10.93 | 62.42 | 64.57 | 67.85 | - | - |
|  | 0 | 0.8 | 0.4 | 1 | 10 | 0.92 | | 12.77 | 7.91 | 8.68 | 62.42 | 76.73 | 74.44 | - | - |
|  | 0 | 1 | 0.4 | 1 | 2 | 0.00 | | 9.57 | 4.70 | 2.30 | 71.84 | 86.18 | 93.22 | 9.56 | 20.87 |
|  | 0 | 1 | 0.4 | 1 | 5 | 0.00 | | 6.03 | 2.96 | 1.45 | 82.25 | 91.29 | 95.72 | 6.32 | 17.63 |
|  | 0 | 1 | 0.4 | 1 | 10 | 0.00 | | 6.03 | 2.43 | 1.19 | 82.25 | 92.84 | 96.48 | 5.79 | 16.25 |
|  | 0 | 0.2 | 0.6 | 1 | 2 | 1.28 | | 27.16 | 28.57 | 29.02 | 20.08 | 15.91 | 14.61 | - | - |
|  | 0 | 0.2 | 0.6 | 1 | 5 | 1.28 | | 19.99 | 25.56 | 27.46 | 41.17 | 24.80 | 19.18 | - | - |
|  | 0 | 0.2 | 0.6 | 1 | 10 | 1.28 | | 19.99 | 16.98 | 23.26 | 41.17 | 50.02 | 31.55 | - | - |
|  | 0 | 0.4 | 0.6 | 1 | 2 | 1.16 | | 23.96 | 24.37 | 24.26 | 29.49 | 28.29 | 28.62 | - | - |
|  | 0 | 0.4 | 0.6 | 1 | 5 | 1.16 | | 17.38 | 21.25 | 22.53 | 48.86 | 37.48 | 33.70 | - | - |
|  | 0 | 0.4 | 0.6 | 1 | 10 | 1.16 | | 17.38 | 13.79 | 18.39 | 48.86 | 59.41 | 45.88 | - | - |
|  | 0 | 0.6 | 0.6 | 1 | 2 | 1.02 | | 20.26 | 19.43 | 18.61 | 40.37 | 42.84 | 45.22 | - | - |
|  | 0 | 0.6 | 0.6 | 1 | 5 | 1.02 | | 14.48 | 16.42 | 16.81 | 57.40 | 51.67 | 50.53 | - | - |
|  | 0 | 0.6 | 0.6 | 1 | 10 | 1.02 | | 14.48 | 10.40 | 13.25 | 57.40 | 69.40 | 61.00 | - | - |
|  | 0 | 0.8 | 0.6 | 1 | 2 | 0.81 | | 15.83 | 13.46 | 11.81 | 53.42 | 60.40 | 65.24 | - | - |
|  | 0 | 0.8 | 0.6 | 1 | 5 | 0.81 | | 10.88 | 10.99 | 10.25 | 67.98 | 67.66 | 69.85 | - | - |
|  | 0 | 0.8 | 0.6 | 1 | 10 | 0.81 | | 10.88 | 6.52 | 7.86 | 67.98 | 80.81 | 76.88 | - | - |
|  | 0 | 1 | 0.6 | 1 | 2 | 0.00 | | 7.22 | 3.54 | 1.74 | 78.77 | 89.58 | 94.89 | 7.58 | 18.89 |
|  | 0 | 1 | 0.6 | 1 | 5 | 0.00 | | 5.05 | 2.48 | 1.22 | 85.13 | 92.70 | 96.42 | 5.08 | 16.38 |
|  | 0 | 1 | 0.6 | 1 | 10 | 0.00 | | 5.05 | 2.38 | 1.17 | 85.13 | 93.01 | 96.57 | 5.01 | 16.09 |
|  | 0 | 0.2 | 0.8 | 1 | 2 | 1.14 | | 26.01 | 28.01 | 28.72 | 23.44 | 17.58 | 15.49 | - | - |
|  | 0 | 0.2 | 0.8 | 1 | 5 | 1.14 | | 18.28 | 24.59 | 26.93 | 46.21 | 27.64 | 20.74 | - | - |
|  | 0 | 0.2 | 0.8 | 1 | 10 | 1.14 | | 18.28 | 15.21 | 22.07 | 46.21 | 55.24 | 35.05 | - | - |
|  | 0 | 0.4 | 0.8 | 1 | 2 | 1.03 | | 22.73 | 23.71 | 23.89 | 33.12 | 30.23 | 29.70 | - | - |
|  | 0 | 0.4 | 0.8 | 1 | 5 | 1.03 | | 15.87 | 20.32 | 21.99 | 53.29 | 40.20 | 35.30 | - | - |
|  | 0 | 0.4 | 0.8 | 1 | 10 | 1.03 | | 15.87 | 12.32 | 17.34 | 53.29 | 63.76 | 48.97 | - | - |
|  | 0 | 0.6 | 0.8 | 1 | 2 | 0.90 | | 18.95 | 18.67 | 18.16 | 44.25 | 45.07 | 46.56 | - | - |
|  | 0 | 0.6 | 0.8 | 1 | 5 | 0.90 | | 13.18 | 15.62 | 16.30 | 61.21 | 54.04 | 52.04 | - | - |
|  | 0 | 0.6 | 0.8 | 1 | 10 | 0.90 | | 13.18 | 9.18 | 12.45 | 61.21 | 72.99 | 63.37 | - | - |
|  | 0 | 0.8 | 0.8 | 1 | 2 | 0.72 | | 14.45 | 12.63 | 11.29 | 57.48 | 62.85 | 66.78 | - | - |
| Chemotherapy + IEC | 0 | 0.8 | 0.8 | 1 | 5 | 0.72 | | 9.79 | 10.41 | 9.87 | 71.19 | 69.37 | 70.97 | - | - |
|  | 0 | 0.8 | 0.8 | 1 | 10 | 0.72 | | 9.79 | 5.66 | 7.33 | 71.19 | 83.34 | 78.43 | 8.01 | - |
|  | 0 | 1 | 0.8 | 1 | 2 | 0.00 | | 5.66 | 2.78 | 1.36 | 83.33 | 91.82 | 95.98 | 5.88 | 17.19 |
|  | 0 | 1 | 0.8 | 1 | 5 | 0.00 | | 4.85 | 2.38 | 1.17 | 85.72 | 92.99 | 96.56 | 4.79 | 16.10 |
|  | 0 | 1 | 0.8 | 1 | 10 | 0.00 | | 4.85 | 2.37 | 1.17 | 85.72 | 93.01 | 96.57 | 4.79 | 16.08 |
| Chemotherapy + environmental modification | 0.2 | 0 | 0.4 | 1 | 2 | 1.46 | | 29.90 | 30.37 | 30.39 | 12.00 | 10.63 | 10.57 | - | - |
|  | 0.2 | 0 | 0.4 | 1 | 5 | 1.46 | | 23.71 | 27.97 | 29.17 | 30.23 | 17.70 | 14.17 | - | - |
|  | 0.2 | 0 | 0.4 | 1 | 10 | 1.46 | | 23.71 | 20.55 | 25.82 | 30.23 | 39.52 | 24.02 | - | - |
|  | 0.4 | 0 | 0.4 | 1 | 2 | 1.32 | | 28.46 | 27.47 | 26.67 | 16.26 | 19.15 | 21.51 | - | - |
|  | 0.4 | 0 | 0.4 | 1 | 5 | 1.32 | | 22.19 | 24.82 | 25.22 | 34.70 | 26.95 | 25.77 | - | - |
|  | 0.4 | 0 | 0.4 | 1 | 10 | 1.32 | | 22.19 | 17.76 | 21.63 | 34.70 | 47.74 | 36.36 | - | - |
|  | 0.6 | 0 | 0.4 | 1 | 2 | 1.16 | | 26.81 | 23.94 | 21.98 | 21.09 | 29.56 | 35.32 | - | - |
|  | 0.6 | 0 | 0.4 | 1 | 5 | 1.16 | | 20.49 | 21.04 | 20.29 | 39.72 | 38.10 | 40.29 | - | - |
|  | 0.6 | 0 | 0.4 | 1 | 10 | 1.16 | | 20.49 | 14.59 | 16.67 | 39.72 | 57.07 | 50.95 | - | - |
|  | 0.8 | 0 | 0.4 | 1 | 2 | 0.92 | | 24.92 | 19.48 | 15.93 | 26.67 | 42.68 | 53.11 | - | - |
|  | 0.8 | 0 | 0.4 | 1 | 5 | 0.92 | | 18.51 | 16.39 | 14.08 | 45.52 | 51.75 | 58.56 | - | - |
|  | 0.8 | 0 | 0.4 | 1 | 10 | 0.92 | | 18.51 | 10.76 | 10.93 | 45.52 | 68.32 | 67.84 | - | - |
|  | 1 | 0 | 0.4 | 1 | 2 | 0.00 | | 22.64 | 12.76 | 6.41 | 33.38 | 62.46 | 81.13 | 16.76 | 28.08 |
|  | 1 | 0 | 0.4 | 1 | 5 | 0.00 | | 16.04 | 9.90 | 5.01 | 52.80 | 70.87 | 85.24 | 15.02 | 26.36 |
|  | 1 | 0 | 0.4 | 1 | 10 | 0.00 | | 16.04 | 5.27 | 2.81 | 52.80 | 84.50 | 91.74 | 10.51 | 22.31 |
|  | 0.2 | 0 | 0.6 | 1 | 2 | 1.28 | | 28.66 | 29.77 | 30.08 | 15.66 | 12.38 | 11.49 | - | - |
|  | 0.2 | 0 | 0.6 | 1 | 5 | 1.28 | | 21.15 | 26.70 | 28.48 | 37.76 | 21.44 | 16.18 | - | - |
|  | 0.2 | 0 | 0.6 | 1 | 10 | 1.28 | | 21.15 | 17.83 | 24.19 | 37.76 | 47.54 | 28.82 | - | - |
|  | 0.4 | 0 | 0.6 | 1 | 2 | 1.16 | | 27.20 | 26.84 | 26.32 | 19.97 | 21.02 | 22.56 | - | - |
|  | 0.4 | 0 | 0.6 | 1 | 5 | 1.16 | | 19.73 | 23.51 | 24.47 | 41.94 | 30.81 | 27.99 | - | - |
|  | 0.4 | 0 | 0.6 | 1 | 10 | 1.16 | | 19.73 | 15.33 | 20.03 | 41.94 | 54.88 | 41.04 | - | - |
|  | 0.6 | 0 | 0.6 | 1 | 2 | 1.02 | | 25.55 | 23.27 | 21.58 | 24.82 | 31.53 | 36.48 | - | - |
|  | 0.6 | 0 | 0.6 | 1 | 5 | 1.02 | | 18.15 | 19.73 | 19.50 | 46.60 | 41.93 | 42.62 | - | - |
|  | 0.6 | 0 | 0.6 | 1 | 10 | 1.02 | | 18.15 | 12.52 | 15.27 | 46.60 | 63.16 | 55.05 | - | - |
|  | 0.8 | 0 | 0.6 | 1 | 2 | 0.81 | | 23.66 | 18.81 | 15.53 | 30.37 | 44.66 | 54.30 | - | - |
|  | 0.8 | 0 | 0.6 | 1 | 5 | 0.81 | | 16.32 | 15.20 | 13.33 | 51.98 | 55.27 | 60.76 | - | - |
|  | 0.8 | 0 | 0.6 | 1 | 10 | 0.81 | | 16.32 | 9.10 | 9.94 | 51.98 | 73.23 | 70.75 | - | - |
|  | 1 | 0 | 0.6 | 1 | 2 | 0.00 | | 21.43 | 12.20 | 6.14 | 36.93 | 64.11 | 81.94 | 16.45 | 27.77 |
|  | 1 | 0 | 0.6 | 1 | 5 | 0.00 | | 14.02 | 9.05 | 4.60 | 58.73 | 73.35 | 86.46 | 14.41 | 25.76 |
|  | 1 | 0 | 0.6 | 1 | 10 | 0.00 | | 14.02 | 4.51 | 2.45 | 58.73 | 86.74 | 92.80 | 9.01 | 21.36 |
|  | 0.2 | 0 | 0.8 | 1 | 2 | 1.14 | | 27.60 | 29.25 | 29.80 | 18.77 | 13.92 | 12.30 | - | - |
|  | 0.2 | 0 | 0.8 | 1 | 5 | 1.14 | | 19.36 | 25.72 | 27.95 | 43.02 | 24.32 | 17.76 | - | - |
|  | 0.2 | 0 | 0.8 | 1 | 10 | 1.14 | | 19.36 | 15.98 | 22.96 | 43.02 | 52.97 | 32.43 | - | - |
|  | 0.4 | 0 | 0.8 | 1 | 2 | 1.03 | | 26.14 | 26.29 | 26.01 | 23.07 | 22.64 | 23.47 | - | - |
|  | 0.4 | 0 | 0.8 | 1 | 5 | 1.03 | | 18.06 | 22.55 | 23.91 | 46.84 | 33.65 | 29.65 | - | - |
|  | 0.4 | 0 | 0.8 | 1 | 10 | 1.03 | | 18.06 | 13.74 | 18.90 | 46.84 | 59.56 | 44.38 | - | - |
|  | 0.6 | 0 | 0.8 | 1 | 2 | 0.90 | | 24.51 | 22.71 | 21.25 | 27.88 | 33.18 | 37.47 | - | - |
| Chemotherapy + environmental modification | 0.6 | 0 | 0.8 | 1 | 5 | 0.90 | | 16.63 | 18.83 | 18.93 | 51.07 | 44.59 | 44.28 | - | - |
|  | 0.6 | 0 | 0.8 | 1 | 10 | 0.90 | | 16.63 | 11.18 | 14.35 | 51.07 | 67.09 | 57.76 | - | - |
|  | 0.8 | 0 | 0.8 | 1 | 2 | 0.72 | | 22.66 | 18.26 | 15.20 | 33.32 | 46.27 | 55.28 | - | - |
|  | 0.8 | 0 | 0.8 | 1 | 5 | 0.72 | | 14.96 | 14.44 | 12.85 | 55.99 | 57.51 | 62.19 | - | - |
|  | 0.8 | 0 | 0.8 | 1 | 10 | 0.72 | | 14.96 | 8.05 | 9.34 | 55.99 | 76.31 | 72.53 | - | - |
|  | 1 | 0 | 0.8 | 1 | 2 | 0.00 | | 20.50 | 11.77 | 5.93 | 39.66 | 65.38 | 82.56 | 16.21 | 27.53 |
|  | 1 | 0 | 0.8 | 1 | 5 | 0.00 | | 12.84 | 8.59 | 4.38 | 62.20 | 74.71 | 87.12 | 14.05 | 25.41 |
|  | 1 | 0 | 0.8 | 1 | 10 | 0.00 | | 12.84 | 4.18 | 2.29 | 62.20 | 87.71 | 93.25 | 9.01 | 20.90 |
| Chemotherapy + IEC + environmental modification | 0.4 | 0.2 | 0.4 | 1 | 2 | 1.23 | | 26.09 | 24.44 | 23.22 | 23.23 | 28.09 | 31.68 | - | - |
|  | 0.4 | 0.2 | 0.4 | 1 | 5 | 1.23 | | 20.02 | 21.66 | 21.63 | 41.08 | 36.27 | 36.34 | - | - |
|  | 0.4 | 0.2 | 0.4 | 1 | 10 | 1.23 | | 20.02 | 15.19 | 18.07 | 41.08 | 55.30 | 46.81 | - | - |
|  | 0.6 | 0.2 | 0.4 | 1 | 2 | 1.07 | | 24.65 | 21.33 | 19.08 | 27.45 | 37.23 | 43.86 | - | - |
|  | 0.6 | 0.2 | 0.4 | 1 | 5 | 1.07 | | 18.56 | 18.38 | 17.32 | 45.37 | 45.90 | 49.03 | - | - |
|  | 0.6 | 0.2 | 0.4 | 1 | 10 | 1.07 | | 18.56 | 12.53 | 13.94 | 45.37 | 63.13 | 58.98 | - | - |
|  | 0.8 | 0.2 | 0.4 | 1 | 2 | 0.85 | | 23.01 | 17.48 | 13.90 | 32.28 | 48.55 | 59.08 | - | - |
|  | 0.8 | 0.2 | 0.4 | 1 | 5 | 0.85 | | 16.87 | 14.46 | 12.07 | 50.37 | 57.45 | 64.47 | - | - |
|  | 0.8 | 0.2 | 0.4 | 1 | 10 | 0.85 | | 16.87 | 9.29 | 9.24 | 50.37 | 72.66 | 72.81 | - | - |
|  | 0.4 | 0.4 | 0.4 | 1 | 2 | 1.12 | | 23.43 | 21.03 | 19.35 | 31.05 | 38.11 | 43.07 | - | - |
|  | 0.4 | 0.4 | 0.4 | 1 | 5 | 1.12 | | 17.67 | 18.19 | 17.66 | 48.01 | 46.46 | 48.03 | - | - |
|  | 0.4 | 0.4 | 0.4 | 1 | 10 | 1.12 | | 17.67 | 12.50 | 14.37 | 48.01 | 63.20 | 57.72 | - | - |
|  | 0.6 | 0.4 | 0.4 | 1 | 2 | 0.98 | | 22.24 | 18.44 | 15.91 | 34.56 | 45.74 | 53.19 | - | - |
|  | 0.6 | 0.4 | 0.4 | 1 | 5 | 0.98 | | 16.46 | 15.53 | 14.13 | 51.56 | 54.29 | 58.42 | - | - |
|  | 0.6 | 0.4 | 0.4 | 1 | 10 | 0.98 | | 16.46 | 10.35 | 11.17 | 51.56 | 69.53 | 67.14 | - | - |
|  | 0.8 | 0.4 | 0.4 | 1 | 2 | 0.77 | | 20.89 | 15.28 | 11.71 | 38.54 | 55.04 | 65.55 | - | - |
|  | 0.8 | 0.4 | 0.4 | 1 | 5 | 0.77 | | 15.05 | 12.39 | 9.94 | 55.72 | 63.54 | 70.74 | - | - |
|  | 0.8 | 0.4 | 0.4 | 1 | 10 | 0.77 | | 15.05 | 7.73 | 7.45 | 55.72 | 77.26 | 78.08 | 42.65 | - |
|  | 0.4 | 0.6 | 0.4 | 1 | 2 | 0.98 | | 20.38 | 17.14 | 14.97 | 40.03 | 49.55 | 55.94 | - | - |
|  | 0.4 | 0.6 | 0.4 | 1 | 5 | 0.98 | | 15.01 | 14.39 | 13.27 | 55.84 | 57.65 | 60.94 | - | - |
|  | 0.4 | 0.6 | 0.4 | 1 | 10 | 0.98 | | 15.01 | 9.60 | 10.55 | 55.84 | 71.75 | 68.95 | - | - |
|  | 0.6 | 0.6 | 0.4 | 1 | 2 | 0.85 | | 19.47 | 15.17 | 12.39 | 42.69 | 55.35 | 63.53 | - | - |
|  | 0.6 | 0.6 | 0.4 | 1 | 5 | 0.85 | | 14.06 | 12.44 | 10.69 | 58.62 | 63.40 | 68.53 | - | - |
|  | 0.6 | 0.6 | 0.4 | 1 | 10 | 0.85 | | 14.06 | 7.98 | 8.27 | 58.62 | 76.50 | 75.67 | - | - |
|  | 0.8 | 0.6 | 0.4 | 1 | 2 | 0.68 | | 18.45 | 12.78 | 9.26 | 45.70 | 62.39 | 72.75 | 28.09 | - |
|  | 0.8 | 0.6 | 0.4 | 1 | 5 | 0.68 | | 12.95 | 10.11 | 7.63 | 61.88 | 70.26 | 77.55 | 24.64 | - |
|  | 0.8 | 0.6 | 0.4 | 1 | 10 | 0.68 | | 12.95 | 6.06 | 5.46 | 61.88 | 82.17 | 83.93 | 17.57 | - |
|  | 0.4 | 0.2 | 0.6 | 1 | 2 | 1.08 | | 24.71 | 23.71 | 22.80 | 27.30 | 30.23 | 32.92 | - | - |
|  | 0.4 | 0.2 | 0.6 | 1 | 5 | 1.08 | | 17.70 | 20.34 | 20.84 | 47.92 | 40.14 | 38.66 | - | - |
|  | 0.4 | 0.2 | 0.6 | 1 | 10 | 1.08 | | 17.70 | 13.04 | 16.61 | 47.92 | 61.63 | 51.12 | - | - |
|  | 0.6 | 0.2 | 0.6 | 1 | 2 | 0.94 | | 23.27 | 20.58 | 18.63 | 31.52 | 39.45 | 45.19 | - | - |
|  | 0.6 | 0.2 | 0.6 | 1 | 5 | 0.94 | | 16.35 | 17.12 | 16.52 | 51.88 | 49.63 | 51.38 | - | - |
|  | 0.6 | 0.2 | 0.6 | 1 | 10 | 0.94 | | 16.35 | 10.66 | 12.72 | 51.88 | 68.62 | 62.57 | - | - |
|  | 0.8 | 0.2 | 0.6 | 1 | 2 | 0.75 | | 21.65 | 16.74 | 13.46 | 36.29 | 50.74 | 60.40 | - | - |
| Chemotherapy + IEC + environmental modification | 0.8 | 0.2 | 0.6 | 1 | 5 | 0.75 | | 14.78 | 13.33 | 11.36 | 56.51 | 60.78 | 66.58 | - | - |
|  | 0.8 | 0.2 | 0.6 | 1 | 10 | 0.75 | | 14.78 | 7.77 | 8.36 | 56.51 | 77.13 | 75.41 | - | - |
|  | 0.4 | 0.4 | 0.6 | 1 | 2 | 0.98 | | 21.91 | 20.20 | 18.85 | 35.53 | 40.57 | 44.54 | - | - |
|  | 0.4 | 0.4 | 0.6 | 1 | 5 | 0.98 | | 15.50 | 16.92 | 16.86 | 54.38 | 50.21 | 50.38 | - | - |
|  | 0.4 | 0.4 | 0.6 | 1 | 10 | 0.98 | | 15.50 | 10.63 | 13.12 | 54.38 | 68.72 | 61.38 | - | - |
|  | 0.6 | 0.4 | 0.6 | 1 | 2 | 0.86 | | 20.73 | 17.60 | 15.39 | 39.01 | 48.22 | 54.71 | - | - |
|  | 0.6 | 0.4 | 0.6 | 1 | 5 | 0.86 | | 14.38 | 14.34 | 13.36 | 57.67 | 57.79 | 60.67 | - | - |
|  | 0.6 | 0.4 | 0.6 | 1 | 10 | 0.86 | | 14.38 | 8.70 | 10.15 | 57.67 | 74.41 | 70.13 | - | - |
|  | 0.8 | 0.4 | 0.6 | 1 | 2 | 0.68 | | 19.40 | 14.46 | 11.21 | 42.91 | 57.45 | 67.01 | - | - |
|  | 0.8 | 0.4 | 0.6 | 1 | 5 | 0.68 | | 13.07 | 11.34 | 9.28 | 61.54 | 66.62 | 72.69 | 48.84 | - |
|  | 0.8 | 0.4 | 0.6 | 1 | 10 | 0.68 | | 13.07 | 6.40 | 6.66 | 61.54 | 81.17 | 80.41 | 38.73 | - |
|  | 0.4 | 0.6 | 0.6 | 1 | 2 | 0.86 | | 18.71 | 16.19 | 14.39 | 44.95 | 52.35 | 57.66 | - | - |
|  | 0.4 | 0.6 | 0.6 | 1 | 5 | 0.86 | | 13.01 | 13.24 | 12.53 | 61.72 | 61.05 | 63.14 | - | - |
|  | 0.4 | 0.6 | 0.6 | 1 | 10 | 0.86 | | 13.01 | 8.01 | 9.59 | 61.72 | 76.41 | 71.79 | - | - |
|  | 0.6 | 0.6 | 0.6 | 1 | 2 | 0.75 | | 17.82 | 14.23 | 11.81 | 47.56 | 58.12 | 65.24 | - | - |
|  | 0.6 | 0.6 | 0.6 | 1 | 5 | 0.75 | | 12.13 | 11.37 | 10.00 | 64.32 | 66.54 | 70.56 | - | - |
|  | 0.6 | 0.6 | 0.6 | 1 | 10 | 0.75 | | 12.13 | 6.59 | 7.44 | 64.32 | 80.60 | 78.12 | - | - |
|  | 0.8 | 0.6 | 0.6 | 1 | 2 | 0.59 | | 16.83 | 11.88 | 8.71 | 50.46 | 65.05 | 74.36 | 27.05 | - |
|  | 0.8 | 0.6 | 0.6 | 1 | 5 | 0.59 | | 11.10 | 9.16 | 7.03 | 67.35 | 73.06 | 79.30 | 23.05 | - |
|  | 0.8 | 0.6 | 0.6 | 1 | 10 | 0.59 | | 11.10 | 5.00 | 4.79 | 67.35 | 85.29 | 85.92 | 9.01 | - |
|  | 0.4 | 0.2 | 0.8 | 1 | 2 | 0.96 | | 23.58 | 23.09 | 22.44 | 30.62 | 32.04 | 33.97 | - | - |
|  | 0.4 | 0.2 | 0.8 | 1 | 5 | 0.96 | | 16.19 | 19.42 | 20.28 | 52.36 | 42.86 | 40.33 | - | - |
|  | 0.4 | 0.2 | 0.8 | 1 | 10 | 0.96 | | 16.19 | 11.64 | 15.62 | 52.36 | 65.75 | 54.04 | - | - |
|  | 0.6 | 0.2 | 0.8 | 1 | 2 | 0.84 | | 22.17 | 19.96 | 18.25 | 34.77 | 41.26 | 46.29 | - | - |
|  | 0.6 | 0.2 | 0.8 | 1 | 5 | 0.84 | | 14.96 | 16.28 | 15.99 | 55.98 | 52.10 | 52.96 | - | - |
|  | 0.6 | 0.2 | 0.8 | 1 | 10 | 0.84 | | 14.96 | 9.46 | 11.94 | 55.98 | 72.18 | 64.86 | - | - |
|  | 0.8 | 0.2 | 0.8 | 1 | 2 | 0.67 | | 20.59 | 16.15 | 13.10 | 39.42 | 52.47 | 61.46 | - | - |
|  | 0.8 | 0.2 | 0.8 | 1 | 5 | 0.67 | | 13.51 | 12.64 | 10.92 | 60.24 | 62.81 | 67.87 | - | - |
|  | 0.8 | 0.2 | 0.8 | 1 | 10 | 0.67 | | 13.51 | 6.84 | 7.82 | 60.24 | 79.87 | 77.00 | - | - |
|  | 0.4 | 0.4 | 0.8 | 1 | 2 | 0.87 | | 20.70 | 19.51 | 18.43 | 39.08 | 42.58 | 45.76 | - | - |
|  | 0.4 | 0.4 | 0.8 | 1 | 5 | 0.87 | | 14.15 | 16.09 | 16.33 | 58.35 | 52.65 | 51.96 | - | - |
|  | 0.4 | 0.4 | 0.8 | 1 | 10 | 0.87 | | 14.15 | 9.40 | 12.32 | 58.35 | 72.33 | 63.76 | - | - |
|  | 0.6 | 0.4 | 0.8 | 1 | 2 | 0.76 | | 19.55 | 16.92 | 14.97 | 42.46 | 50.20 | 55.94 | - | - |
|  | 0.6 | 0.4 | 0.8 | 1 | 5 | 0.76 | | 13.12 | 13.61 | 12.88 | 61.39 | 59.96 | 62.09 | - | - |
|  | 0.6 | 0.4 | 0.8 | 1 | 10 | 0.76 | | 13.12 | 7.63 | 9.52 | 61.39 | 77.53 | 72.00 | - | - |
|  | 0.8 | 0.4 | 0.8 | 1 | 2 | 0.60 | | 18.28 | 13.83 | 10.82 | 46.22 | 59.31 | 68.15 | - | - |
|  | 0.8 | 0.4 | 0.8 | 1 | 5 | 0.60 | | 11.90 | 10.74 | 8.90 | 64.97 | 68.39 | 73.82 | 47.74 | - |
|  | 0.8 | 0.4 | 0.8 | 1 | 10 | 0.60 | | 11.90 | 5.62 | 6.18 | 64.97 | 83.46 | 81.82 | 9.01 | - |
|  | 0.4 | 0.6 | 0.8 | 1 | 2 | 0.76 | | 17.43 | 15.44 | 13.92 | 48.70 | 54.56 | 59.04 | - | - |
|  | 0.4 | 0.6 | 0.8 | 1 | 5 | 0.76 | | 11.81 | 12.55 | 12.07 | 65.24 | 63.07 | 64.47 | - | - |
|  | 0.4 | 0.6 | 0.8 | 1 | 10 | 0.76 | | 11.81 | 7.00 | 8.98 | 65.24 | 79.41 | 73.59 | 9.01 | - |
|  | 0.6 | 0.6 | 0.8 | 1 | 2 | 0.67 | | 16.58 | 13.51 | 11.36 | 51.20 | 60.25 | 66.58 | - | - |
| Chemotherapy + IEC + environmental modification | 0.6 | 0.6 | 0.8 | 1 | 5 | 0.67 | | 10.99 | 10.77 | 9.61 | 67.65 | 68.32 | 71.73 | - | - |
|  | 0.6 | 0.6 | 0.8 | 1 | 10 | 0.67 | | 10.99 | 5.75 | 6.92 | 67.65 | 83.08 | 79.65 | 9.01 | - |
|  | 0.8 | 0.6 | 0.8 | 1 | 2 | 0.53 | | 15.65 | 11.20 | 8.30 | 53.95 | 67.03 | 75.58 | 26.18 | - |
|  | 0.8 | 0.6 | 0.8 | 1 | 5 | 0.53 | | 10.05 | 8.64 | 6.71 | 70.44 | 74.56 | 80.24 | 22.10 | - |
|  | 0.8 | 0.6 | 0.8 | 1 | 10 | 0.53 | | 10.05 | 4.43 | 4.40 | 70.44 | 86.96 | 87.05 | 8.01 | - |
| IEC + environmental modification | 0.2 | 0.2 | 0 | 1 | 2 | 2.12 | | 30.65 | 28.60 | 27.43 | 9.80 | 15.85 | 19.29 | - | - |
|  | 0.4 | 0.2 | 0 | 1 | 2 | 1.93 | | 29.48 | 26.13 | 24.17 | 13.25 | 23.11 | 28.87 | - | - |
|  | 0.6 | 0.2 | 0 | 1 | 2 | 1.68 | | 28.14 | 23.13 | 20.14 | 17.19 | 31.92 | 40.73 | - | - |
|  | 0.8 | 0.2 | 0 | 1 | 2 | 1.34 | | 26.58 | 19.35 | 15.01 | 21.78 | 43.05 | 55.81 | - | - |
|  | 0.9 | 0.2 | 0 | 1 | 2 | 1.06 | | 25.68 | 16.93 | 11.74 | 24.44 | 50.17 | 65.45 | 31.96 | - |
|  | 1 | 0.2 | 0 | 1 | 2 | 0.00 | | 24.65 | 13.45 | 6.72 | 27.48 | 60.43 | 80.22 | 17.09 | 28.41 |
|  | 0.2 | 0.4 | 0 | 1 | 2 | 1.93 | | 28.25 | 25.12 | 23.30 | 16.86 | 26.09 | 31.45 | - | - |
|  | 0.4 | 0.4 | 0 | 1 | 2 | 1.75 | | 27.27 | 23.02 | 20.51 | 19.74 | 32.26 | 39.65 | - | - |
|  | 0.6 | 0.4 | 0 | 1 | 2 | 1.53 | | 26.16 | 20.51 | 17.14 | 23.02 | 39.63 | 49.55 | - | - |
|  | 0.8 | 0.4 | 0 | 1 | 2 | 1.21 | | 24.87 | 17.39 | 12.96 | 26.83 | 48.82 | 61.86 | - | - |
|  | 0.9 | 0.4 | 0 | 1 | 2 | 0.96 | | 24.12 | 15.38 | 10.27 | 29.03 | 54.73 | 69.78 | 26.24 | - |
|  | 1 | 0.4 | 0 | 1 | 2 | 0.00 | | 23.26 | 12.53 | 6.24 | 31.55 | 63.14 | 81.63 | 16.57 | 27.88 |
|  | 0.2 | 0.6 | 0 | 1 | 2 | 1.68 | | 25.53 | 21.12 | 18.54 | 24.88 | 37.84 | 45.44 | - | - |
|  | 0.4 | 0.6 | 0 | 1 | 2 | 1.53 | | 24.77 | 19.49 | 16.38 | 27.10 | 42.63 | 51.81 | - | - |
|  | 0.6 | 0.6 | 0 | 1 | 2 | 1.34 | | 23.92 | 17.57 | 13.83 | 29.61 | 48.28 | 59.29 | - | - |
|  | 0.8 | 0.6 | 0 | 1 | 2 | 1.06 | | 22.92 | 15.18 | 10.68 | 32.54 | 55.32 | 68.56 | 30.39 | - |
|  | 0.9 | 0.6 | 0 | 1 | 2 | 0.84 | | 22.35 | 13.62 | 8.62 | 34.24 | 59.91 | 74.63 | 21.74 | 47.24 |
|  | 1 | 0.6 | 0 | 1 | 2 | 0.00 | | 21.69 | 11.48 | 5.70 | 36.18 | 66.22 | 83.22 | 15.93 | 27.24 |
|  | 0.2 | 0.8 | 0 | 1 | 2 | 1.34 | | 22.27 | 16.34 | 12.91 | 34.46 | 51.92 | 62.02 | - | - |
|  | 0.4 | 0.8 | 0 | 1 | 2 | 1.21 | | 21.79 | 15.31 | 11.56 | 35.89 | 54.96 | 65.97 | - | - |
|  | 0.6 | 0.8 | 0 | 1 | 2 | 1.06 | | 21.23 | 14.08 | 9.98 | 37.51 | 58.57 | 70.63 | 29.08 | - |
|  | 0.8 | 0.8 | 0 | 1 | 2 | 0.84 | | 20.58 | 12.51 | 7.98 | 39.43 | 63.18 | 76.52 | 20.82 | 46.27 |
|  | 0.9 | 0.8 | 0 | 1 | 2 | 0.67 | | 20.20 | 11.50 | 6.68 | 40.54 | 66.16 | 80.33 | 17.79 | 34.47 |
|  | 1 | 0.8 | 0 | 1 | 2 | 0.00 | | 19.78 | 10.20 | 5.05 | 41.80 | 69.97 | 85.15 | 15.07 | 26.38 |
|  | 0.2 | 0.9 | 0 | 1 | 2 | 1.06 | | 20.19 | 13.33 | 9.45 | 40.58 | 60.77 | 72.18 | 27.79 | - |
|  | 0.4 | 0.9 | 0 | 1 | 2 | 0.96 | | 19.87 | 12.65 | 8.59 | 41.54 | 62.78 | 74.72 | 23.54 | - |
|  | 0.6 | 0.9 | 0 | 1 | 2 | 0.84 | | 19.49 | 11.82 | 7.56 | 42.64 | 65.21 | 77.74 | 20.16 | 45.49 |
|  | 0.8 | 0.9 | 0 | 1 | 2 | 0.67 | | 19.05 | 10.78 | 6.29 | 43.93 | 68.27 | 81.50 | 17.22 | 33.88 |
|  | 0.9 | 0.9 | 0 | 1 | 2 | 0.53 | | 18.81 | 10.14 | 5.52 | 44.66 | 70.16 | 83.77 | 15.82 | 29.58 |
|  | 1 | 0.9 | 0 | 1 | 2 | 0.00 | | 18.53 | 9.38 | 4.63 | 45.46 | 72.38 | 86.39 | 14.45 | 25.77 |
|  | 0.2 | 1 | 0 | 1 | 2 | 0.00 | | 16.68 | 8.19 | 4.02 | 50.92 | 75.91 | 88.18 | 13.47 | 24.78 |
|  | 0.4 | 1 | 0 | 1 | 2 | 0.00 | | 16.68 | 8.19 | 4.02 | 50.92 | 75.91 | 88.18 | 13.47 | 24.78 |
|  | 0.6 | 1 | 0 | 1 | 2 | 0.00 | | 16.68 | 8.19 | 4.02 | 50.92 | 75.91 | 88.18 | 13.47 | 24.78 |
|  | 0.8 | 1 | 0 | 1 | 2 | 0.00 | | 16.68 | 8.19 | 4.02 | 50.92 | 75.91 | 88.18 | 13.47 | 24.78 |
|  | 0.9 | 1 | 0 | 1 | 2 | 0.00 | | 16.68 | 8.19 | 4.02 | 50.92 | 75.91 | 88.18 | 13.47 | 24.78 |
|  | 1 | 1 | 0 | 1 | 2 | 0.00 | | 16.68 | 8.19 | 4.02 | 50.92 | 75.91 | 88.18 | 13.47 | 24.78 |

*The parameters were set to the best set of parameter estimates; each control strategy was simulated for 50 years. $C_{d}$, $C_{e}$, $C_{m}$ indicate the coverage of environmental modification, IEC (focus both on improvement of hygiene habits and changing people’s behavior of raw-fish-consumption) and chemotherapy, respectively. $R_{c}$ is the control reproduction number, $P_{s5}$,$P_{s10}$ and $P_{s15}$ indicate the prevalence in 5, 10 and 15 years from the beginning of intervention, respectively. $r_{s5}$, $r_{s10}$ and $r_{s15}$ indicate the reduced rates in 5, 10 and 15 years, compared with the baseline prevalence, respectively. $Y_{5\%}$ and $Y_{1\%}$ indicate the years from the beginning of intervention to infection control and transmission control, respectively. Strategies combined chemotherapy with IEC, combined chemotherapy with environmental modification, combined chemotherapy, IEC with environmental modification, and combined IEC with environmental modification are in green, blue, yellow and grey shades, respectively.
