# Supplementary material for: Assessment of control strategies against Clonorchis sinensis infection based on a multi-group dynamic transmission model
Source: PLoS Negl Trop Dis. 2020 Mar 27;14(3):e0008152. doi: 10.1371/journal.pntd.0008152 (PMC7156112; doi:10.1371/journal.pntd.0008152)
Supplement: S1 Text — (DOCX) [file pntd.0008152.s013.docx]

**S1 Text. The basic transmission model**

$$\left\{ \begin{aligned} &\frac{dS_{h,i}}{dt}=\lambda_{h,i}-c_{i}\beta_{h,1}S_{h,i}I_{f}-\mu_{h}S_{h,i}+\gamma_{1}I_{h,i}, \\ &\frac{dI_{h,i}}{dt}={c_{i}\beta}_{h,1}S_{h,i}I_{f}-\mu_{h}I_{h,i}-\gamma_{1}I_{h,i}, \\ &\frac{dS_{s}}{dt}=\lambda_{s}-\beta_{s}S_{s}\left( I_{h,1}+I_{h,2}+I_{h,3}+I_{h,4} \right)-\mu_{s}S_{s}, \\ &\frac{dI_{s}}{dt}=\beta_{s}S_{s}\left( I_{h,1}+I_{h,2}+I_{h,3}+I_{h,4} \right)-\mu_{s}I_{s}, \\ &\frac{dS_{f}}{dt}=\lambda_{f}-\beta_{f}S_{f}I_{s}-\mu_{f}S_{f}, \\ &\frac{dI_{f}}{dt}=\beta_{f}S_{f}I_{s}-\mu_{f}I_{f}, \end{aligned} \right.$$

Let $S_{h,i}$, $I_{h,i}$, $S_{s}$, $I_{s}$, $S_{f}$ and $I_{f}$ denote the numbers of susceptible human, infected human, susceptible snail, infected snail, susceptible fish and infected fish, respectively. Here $i=1, 2, 3, 4$ indicating the human groups who seldom, moderately, often and very often eating of raw fish, respectively. Humans transfer from susceptible to infected state with the number $c_{i}\beta_{h,1}S_{h,i}I_{f}$ $(i=1, 2, 3, 4$ and $c_{1}=1$), while transfer back after recovery from individual treatment with the number $\gamma_{1}I_{h,i}$. Regards to snail and fish, they transfer from susceptible to infected state with the numbers $\beta_{s}S_{s}\left( I_{h,1}+I_{h,2}+I_{h,3}+I_{h,4} \right)$ and $\beta_{f}S_{f}I_{s}$, respectively, but do not transfer back as no treatment measures taken for intermediate hosts. Hosts enter into the system as susceptible individuals with recruitment number $\lambda$, and leave it with death rate $\mu$. We assumed same numbers of entering and leaving of the system, thus $\lambda_{j}=N_{j}\mu_{j}$ ($j=h_{i}, s,f$ and $i=1, 2, 3, 4$). Here $h_{i}, s,f$ and $N_{j}$ indicate human, snail, fish and the total number of host population, respectively, where $N_{j}=S_{j}+I_{j}$. Under conditions $S_{h,i}\geq0$, $I_{h,i}\geq0$, $S_{s}\geq0$, $I_{s}\geq0$, $S_{f}\geq0$ and $I_{f}\geq0$ and parameters nonnegative, the system has two equilibrium points: (1) the disease free equilibrium point that no disease in the population exists and (2) the endemic equilibrium point that the disease persists in the population with no change of numbers in each state.
