# Supplementary material for: Assessment of control strategies against Clonorchis sinensis infection based on a multi-group dynamic transmission model
Source: PLoS Negl Trop Dis. 2020 Mar 27;14(3):e0008152. doi: 10.1371/journal.pntd.0008152 (PMC7156112; doi:10.1371/journal.pntd.0008152)
Supplement: S2 Text — (DOCX) [file pntd.0008152.s014.docx]

**S2 Text. The full model with interventions**

$$\left\{ \begin{aligned} & \frac{dS_{h,i}}{dt}=\lambda_{h,i}-\left[ \left( 1-C_{e1,i} \right)\beta_{h,1}+(1-C_{e2,i})\beta_{h,i} \right]S_{h,i}I_{f}-\mu_{h}S_{h,i}+\gamma_{1}I_{h,i}+\gamma_{2,i}I_{h,i}, \\ & \frac{dI_{h,i}}{dt}=\left[ \left( 1-C_{e1,i} \right)\beta_{h,1}+(1-C_{e2,i})\beta_{h,i} \right]S_{h,i}I_{f}-\mu_{h}I_{h,i}-\gamma_{1}I_{h,i}-\gamma_{2,i}I_{h,i}, \\ & \frac{dS_{s}}{dt}=\lambda_{s}-{(1-C_{d})\beta}_{s}S_{s}\left( I_{h,1}+I_{h,2}+I_{h,3}+I_{h,4} \right)-\mu_{s}S_{s}, \\ & \frac{dI_{s}}{dt}={(1-C_{d})\beta}_{s}S_{s}\left( I_{h,1}+I_{h,2}+I_{h,3}+I_{h,4} \right)-\mu_{s}I_{s}, \\ & \frac{dS_{f}}{dt}=\lambda_{f}-\beta_{f}S_{f}I_{s}-\mu_{f}S_{f}, \\ & \frac{dI_{f}}{dt}=\beta_{f}S_{f}I_{s}-\mu_{f}I_{f}, \end{aligned} \right.$$

**Preventive chemotherapy** The recovery number of infected population through community chemotherapy against *C. sinensis* infection for each group of humans is expressed as $\gamma_{2,i}I_{h,i} (i=1,2,3,4)$, where $\gamma_{2,i}$ is the recovery rate of infected humans through preventive chemotherapy per unit of time, described as $\gamma_{2,i}=-\frac{\log\left( 1-C_{m,i}h \right)}{T_{i}}$ [1]. $C_{m, i}$ is the coverage of chemotherapy, $T_{i}$is the time interval of treatment in days on the $i$^th^ group of humans, and $h$ is the drug efficacy.

**IEC** Through IEC, people’s healthy behaviors could be improved, which is further categorized into two kinds: (1) improvement of hygiene habits, such as washing hands before eating, using clean tableware for food and avoiding eating unclean food; and (2) changing the behavior of raw-fish-consumption, such as stopping or reducing the frequency of eating raw fish. We assumed that the first improvement will decrease the basic transmission rate (i.e., the transmission rate from an infected fish to a susceptible human who seldom eat raw fish), while the second will reduce the transmission rate of fish to at-risk population due to raw fish consumption. In such case, the basic transmission rate is expressed as $\left( {1-C}_{e1,i} \right)\beta_{h,1}(i=1,2,3,4)$ due to improvement of hygiene habits, where $C_{e1,i}$ indicates the improvement rate of hygiene habits by IEC in the $i$^th^ group of population. The transmission rate due to raw fish consumption can be written as $\left( {1-C}_{e2,i} \right)\beta_{h,i}$ $(i=2,3,4)$, where $C_{e2,i}$ indicates the rate of stopping raw-fish-eating behavior (i.e., proportions of people who stop eating raw fish among those with raw-fish-eating behavior) in the $i$^th^ group of population by IEC. These improvements were assumed continuous, that is, once it happens, it will last through time in the same population.

**Environmental modification** Let $C_{d}$ denote as the coverage of sanitation toilets, which represents the proportion of people who stop defecating to fish ponds due to reconstruction of toilets adjacent. Therefore, the transmission rate from humans to snails is reduced to $\left( {1-C}_{d} \right)\beta_{s}$, which is also assumed continuous through time.

References

1. Bürli C, Harbrecht H, Odermatt P, Sayasone S, Chitnis N. Analysis of interventions against the liver fluke, opisthorchis viverrini. Math Biosci. 2018;(303):115-25.
