# Supplementary material for: Assessment of control strategies against Clonorchis sinensis infection based on a multi-group dynamic transmission model
Source: PLoS Negl Trop Dis. 2020 Mar 27;14(3):e0008152. doi: 10.1371/journal.pntd.0008152 (PMC7156112; doi:10.1371/journal.pntd.0008152)
Supplement: S3 Text — (DOCX) [file pntd.0008152.s015.docx]

**S3 Text. The basic reproduction number** $\boldsymbol{R}_{\boldsymbol{0}}$

The basic reproduction number can be derived by the next generation matrix K. To derive matrix K, we first decomposed the matrix into two matrices, T and $\Sigma$. T is the transmission part which describes the production of new infections, and $\Sigma$ is the transition part which describes the changes in state. K is denoted as -T$\Sigma$, and $R_{0}$ is the spectral radius of K [1,2].

The transmission matrix T is

$$T=\left[ \begin{matrix} 0 & 0 & 0 & 0 & 0 & \frac{{{c_{1}\beta}_{h,1}\lambda}_{h,1}}{\mu_{h}} \\ 0 & 0 & 0 & 0 & 0 & \frac{{c_{2}\beta}_{h,1}\lambda_{h,2}}{\mu_{h}} \\ 0 & 0 & 0 & 0 & 0 & \frac{{c_{3}\beta}_{h,1}\lambda_{h,3}}{\mu_{h}} \\ 0 & 0 & 0 & 0 & 0 & \frac{{{c_{4}\beta}_{h,1}\lambda}_{h,4}}{\mu_{h}} \\ \frac{{\beta_{s}\lambda}_{s}}{\mu_{s}} & \frac{{\beta_{s}\lambda}_{s}}{\mu_{s}} & \frac{\beta_{s}\lambda_{s}}{\mu_{s}} & \frac{\beta_{s}\lambda_{s}}{\mu_{s}} & 0 & 0 \\ 0 & 0 & 0 & 0 & \frac{\beta_{f}\lambda_{f}}{\mu_{f}} & 0 \end{matrix} \right]$$

and the transition matrix $\Sigma$ is

$$\Sigma=\left[ \begin{matrix} -\left( \mu_{h}+\gamma_{1} \right) & 0 & 0 & 0 & 0 & 0 \\ 0 & -\left( \mu_{h}+\gamma_{1} \right) & 0 & 0 & 0 & 0 \\ 0 & 0 & -\left( \mu_{h}+\gamma_{1} \right) & 0 & 0 & 0 \\ 0 & 0 & 0 & -\left( \mu_{h}+\gamma_{1} \right) & 0 & 0 \\ 0 & 0 & 0 & 0 & -\mu_{s} & 0 \\ 0 & 0 & 0 & 0 & 0 & -\mu_{f} \end{matrix} \right]$$

Then, the next generation matrix K of the model is

$$K=-T\Sigma^{-1}=\left[ \begin{matrix} 0 & 0 & 0 & 0 & 0 & \frac{{{c_{1}\beta}_{h,1}\lambda}_{h,1}}{\mu_{h}\mu_{f}} \\ 0 & 0 & 0 & 0 & 0 & \frac{{{c_{2}\beta}_{h,1}\lambda}_{h,2}}{\mu_{h}\mu_{f}} \\ 0 & 0 & 0 & 0 & 0 & \frac{{{c_{3}\beta}_{h,1}\lambda}_{h,3}}{\mu_{h}\mu_{f}} \\ 0 & 0 & 0 & 0 & 0 & \frac{{{c_{4}\beta}_{h,1}\lambda}_{h,4}}{\mu_{h}\mu_{f}} \\ \frac{\beta_{s}\lambda_{s}}{\mu_{s}\left( \mu_{h}+\gamma_{1} \right)} & \frac{{\beta_{s}\lambda}_{s}}{\mu_{s}\left( \mu_{h}+\gamma_{1} \right)} & \frac{{\beta_{s}\lambda}_{s}}{\mu_{s}\left( \mu_{h}+\gamma_{1} \right)} & \frac{{\beta_{s}\lambda}_{s}}{\mu_{s}\left( \mu_{h}+\gamma_{1} \right)} & 0 & 0 \\ 0 & 0 & 0 & 0 & \frac{{\beta_{f}\lambda}_{f}}{\mu_{f}\mu_{s}} & 0 \end{matrix} \right]$$

Therefore, we obtained the formula of the basic reproduction number:

$$R_{0}=\rho\left( -T\Sigma^{-1} \right)=\sqrt[3]{\frac{\beta_{h,1}\beta_{s}\beta_{f}\lambda_{s}\lambda_{f}({c_{1}\lambda}_{h,1}+c_{2}\lambda_{h,2}+c_{3}\lambda_{h,3}+c_{4}\lambda_{h,4})}{\mu_{h}\left( \mu_{h}+\gamma_{1} \right)\mu_{s}^{2}\mu_{f}^{2}}}$$

Using the Corollary 3.2 of Zhao to discuss case of the disease persistence or elimination [3], we conclude the following result.

**Theorem**: If $R_{0}<1$, the disease-free equilibrium of the model is globally asymptotically stable. Otherwise, if $R_{0}>1$, the endemic equilibrium of the model is globally asymptotically stable.

**References**

1. Diekmann O, Heesterbeek JA, Roberts MG. The construction of next-generation matrices for compartmental epidemic models. J R Soc Interface. 2010;7(47):873-885.
2. Dreessche P, Watmough J. Reproduction Numbers and Sub-threshold Endemic Equilibria for Compartmental Models of Disease Transmission. Math Biosci. 2002;180(1-2):29-48.
3. Zhao XQ, Jing ZJ. Global asymptotic behavior in some cooperative systems of functional differential equations. Can Appl Math Q. 1996;4(4):421-44.
