# Supplementary material for: Assessment of control strategies against Clonorchis sinensis infection based on a multi-group dynamic transmission model
Source: PLoS Negl Trop Dis. 2020 Mar 27;14(3):e0008152. doi: 10.1371/journal.pntd.0008152 (PMC7156112; doi:10.1371/journal.pntd.0008152)
Supplement: S4 Text — (DOCX) [file pntd.0008152.s016.docx]

**S4 Text. The prior information, the likelihood function and posterior distributions of parameter estimation**

**Prior** Informative priors based on different sources were set for unknown parameters using triangular distributions, the modes and ranges of which are shown in S2 Table. We set the prior for $\mu_{f}$ according to the culture period of fish [1]. We obtained information of $N_{f}$ based on local fish density and total pond area [2,3], which was further used to set the prior for $\lambda_{f}$ under previous assumption that $\lambda_{j}=N_{j}\mu_{j}$ ($j=h_{i}, s,f$ and $i=1, 2, 3, 4$). The prior for the proportion of population in the $i$^th^ group of humans ($p_{i}$, $i=1,2,3,4$) were set according to the observed data, which was further adjusted to $p_{i}^{'}$so that the sum of $p_{i}^{'}$ for all groups equals to one. $\lambda_{h,i}$ was calculated based on the known $\mu_{h}$, $N_{h}$ and the estimated $p_{i}$ [4]. On the endemic equilibrium point, the derivatives in the system of basic model equal to zero, thus we have $c_{i}=\frac{{I_{h,i}}/{S_{h,i}}}{{I_{h,1}}/{S_{h,1}}}(i=2,3,4)$, that is $c_{i}$ equals to odds ratio of the $i$^th^ and the first group of human. With information of the observed prevalence, we set the prior for $c_{i}$. Given the modes of prior distributions for the above parameters, we calculated the prior modes for transmission rates $\beta_{i} (i=h_{1},s,f)$ by solving equations of the basic model on the endemic equilibrium point. The prior set for the basic recovery rate of infected humans $\gamma_{1}$ was according to the individual treatment rate of clonorchiasis from a survey in Shunde District [5], an endemic district near Zhongshan in Guangdong Province.

**Likelihood** The likelihood function was defined as $L=L_{h,1}L_{h,2}L_{h,3}L_{h,4}L_{s}L_{f}L_{p}$, which refers to the probability given the observed data. The likelihoods of humans ($L_{h,i}$, $i=1, 2, 3, 4$) and fish $L_{f}$ were assumed follow binomial distribution such that $L_{j}=C_{M_{j}}^{Y_{j}}P_{j}^{Y_{j}}(1-P_{j})^{M_{j}-Y_{j}}$ ($j=h_{i}, f$ and $i=1, 2, 3, 4$), where $M_{j}$, $Y_{j}$ and $P_{j}$ indicates total number of examined, the number of positive in observed data, and the endemic equilibrium prevalence from the solutions of differential equations in the $j$ group of population. Without observed data for snail, we assumed the corresponding likelihood $L_{s}$ follows a beta distribution with mean $P_{s}$ (the endemic equilibrium prevalence) and 95% interval lying between 0.1% and 3.7%. This interval was defined according to snail surveys in different endemic areas of Guangdong province [6,7]. The likelihood for proportions of humans in groups of different raw-fish-eating frequencies ($L_{p}$) was assumed to follow a multinomial distribution, that is $L_{p}=M_{p}!\prod_{i=1}^{4} p_{i}^{'m_{i}}/m_{i}!$, where $M_{p}$, $m_{i}$ and $p_{i}^{'}$ indicate the total number of observed, the number categorized as the $i^{th}$ group of humans and the corresponding adjusted proportion sampling from prior distribution.

**Posterior** The posterior distributions of the estimated parameters were obtained through the sampling-importance-resampling procedure [8]. We randomly sampled with replacement from the prior distributions 100,000 parameter vectors, filtering out those corresponding to $R_{0}<1$ with consideration that $R_{0}$ should be greater than one in the current endemic area. Then we obtained the corresponding model outputs (i.e., the endemic equilibrium prevalence $P_{j}$ and the adjusted proportion of different human group $p_{i}^{'}$ for $j=h_{i}, s, f$ and $i=1, 2, 3, 4$) by solving the differential equations of the basic model on endemic equilibrium and calculated the corresponding likelihood values. Finally, we resampled 500 parameter vectors with replacement from the ones draw in the first step, with probabilities proportional to the posterior values (i.e., product of probabilities of the parameters according to their prior distributions and the relative likelihood values), based on which we obtained the estimated posterior prevalence of hosts. In addition, we identified the best set of estimated parameters as the one with the highest posterior values among the 500 draws [9]. All the numerical computations were performed in MATLAB R2019b.

**References**

1. Liu JJ, Zhang JY, Yuan H. [Investigation and analysis of cost-benefit of freshwater fish culture]. Chinese Fisheries Economics. 2017;35(1):18-27. Chinese.

2. Xie XQ [Internet]. [The rent of fish pond in Zhongshan, Guangdong Province is up to 4750 yuan per mu, which breaking the record ] (author’s tranl). Nan Fang Nong Cun Bao. c2019 [cited 2019 Oct 20]. Available from: http://www.bbwfish.com/article.asp?artid=86551. Chinese.

3. [How many fish can be raised in one mu of fishpond?] (author’s tranl) [Internet]. c2019 [cited 2019 Oct 20]. Available from: <https://zhidao.baidu.com/question/1430988985257871219.html>. Chinese.

4. Zhongshan Statistical Bureau [Internet]. [Zhongshan Statistical Yearbook 2013]. c2019 [cited 2019 Oct 20]. Available from: http://stats.zs.gov.cn/tjzl/tjnj/2013nj/index.htm. Chinese.

5. Ma JQ, Chen GQ, Tang X, Zuo ZH, Cao FP, Gong F, et al. [Survey of Life Habits and Health Knowledge of the People in Epidemic Region of Clonorchiasis]. Re Dai Yi Xue Za Zhi. 2008;8(8):858-60,865. Chinese.

6. Zhang XC, Pei FQ, Zhang QM, Lin RX, Huang SY, Wang JL, et al. [Current status of environmental sanitation and clonorchis sinensis intermediate host infection of freshwater aquaculture in partial areas of Guangdong Province]. South China J Prev Med. 2010;36(3):9-13. Chinese.

7. LI FL, Lin RX, Huang BM, Zhou YL, Ou BW, Luo CH, et al. [Epidemiological investigation on clonorchiasis and exploration of treatment measures in urban district of jiangmen city]. Zhongguo Ji Sheng Chong Bing Fang Zhi Za Zhi. 2005;18(3):214-6. Chinese.

8. Rubin DS, Rubin DB. Using the SIR algorithm to simulate posterior distributions. Bayesian statistics 3. 1988:395–402.

9. Alkema L, Raftery AE, Brown T. Bayesian melding for estimating uncertainty in national HIV prevalence estimates. Sex Transm Infect. 2008;(84 Suppl 1):i11-i6.
