# Supplementary material for: Assessment of control strategies against Clonorchis sinensis infection based on a multi-group dynamic transmission model
Source: PLoS Negl Trop Dis. 2020 Mar 27;14(3):e0008152. doi: 10.1371/journal.pntd.0008152 (PMC7156112; doi:10.1371/journal.pntd.0008152)
Supplement: Attachment — Submitted filename: point-to-point response.docx [file pntd.0008152.s017.docx]

Guangzhou, 15 Januray 2020

Dear Dr. Chai,

Dear Dr. Sripa,

We refer to our manuscript titled “**Assessment of control strategies against *Clonorchis sinensis* infection based on a multi-group dynamic transmission model**” (your reference No. **PNTD-D-19-01903**). Many thanks to you and the three reviewers for the excellent comments and suggestions. We have addressed all points put forward by reviewers in the revised manuscript and provide our point-by-point response below.

***********************

This manuscript describes about control strategies of clonorchiasis in China. It is interesting but needs some revisions before finally accepted. Please read carefully the reviewers' comments and properly address the points. Perhaps something like a flow diagram of the control measure(s) can be added for better understanding of the study. Please consider reducing unnecessary statements throughout the manuscript. The figure quality needs to be improved.
**Reply:**

We thank the editors’ comments. The reviewers’ points are addressed below. We added a flow diagram to better clarify the study and the control measures (**Fig 1 in the revised manuscript**). A paragraph about method overview was added for readers to better understand the overall methodology we used (**lines 165-179 in the revised manuscript**). In addition, we deleted unnecessary sentences, phrases and words, and moved some details in the methodology to the supporting information, due to the over-length of the manuscript. **Please see the last part of this letter for the detailed content we changed**. The number of words in the main text is reduced to 6646 compared to 7472 in the original manuscript. Furthermore, we improved the resolution of the figures according to the journal’s requirements.

Reviewer's Responses to Questions
Key Review Criteria Required for Acceptance?
As you describe the new analyses required for acceptance, please consider the following:
Methods
-Are the objectives of the study clearly articulated with a clear testable hypothesis stated?
-Is the study design appropriate to address the stated objectives?
-Is the population clearly described and appropriate for the hypothesis being tested?
-Is the sample size sufficient to ensure adequate power to address the hypothesis being tested?
-Were correct statistical analysis used to support conclusions?
-Are there concerns about ethical or regulatory requirements being met?

Reviewer #1: The objectives are clear, and the methods of statistical analysis seems to be good enough.
**Reply:**

We thank Reviewer #1 for the positive comment on the methods we applied.

Reviewer #2: The manuscript described deterministic compartment control model for clonorchiasis by aiming for a long term outcome. Overall the objective is clear and by simulation of the model, the long term out comes can be assessed. The field data from an endemic area was used to validate the model prediction but there is no statistical support for that?.

**Reply:**

We thank Reviewer #2 for arising this point. Indeed, we validated the model by comparing the posterior estimated prevalence and the observed prevalence (**lines 248-250 in the revised manuscript**). We found that all observed prevalence fell within the 95% credible intervals of the estimated prevalence (**S2** **Fig and lines 321-322 in the revised manuscript**), suggesting a good fitting capacity of the model. To show a better statistical support, we further calculated the modeling efficiency statistic (EF) for the goodness-of-fit and did the Chi-square test to see whether there is significant difference between observed and estimated prevalence. The EF score was approximately equal to 1 (*EF*=0.9998) and the Chi-square value was 20, with *P*-value equal to 0.22 (larger than the significant level 0.05), both indicating good fitting capacity of our model (**lines 248-253 for methodology and lines 322-324 for results in the revised manuscript**).

Reviewer #3: (No Response)
--------------------

Results
-Does the analysis presented match the analysis plan?
-Are the results clearly and completely presented?
-Are the figures (Tables, Images) of sufficient quality for clarity?

Reviewer #1: The result was described clearly.
**Reply:**

We thank Reviewer #1 for the positive comment on the results.

Reviewer #2: I think for general reader who are not statistician, the results presented should be improved in a way that readers can easily follow the results. Perhaps something like a flow diagram of the control measure(s) can be added. Also for example the data in Figure 2 for human 1 ,2 and 3 in X-axis needed more clarification whether it means 3 human cases or not. I am not sure the quality of Figure 1 is adequate.
**Reply:**

We added a flow diagram to better clarify the study and the control measures we planned to assess (**Fig 1 in the revised manuscript**). In the part of results, we added subheadings for the control measures that we assessed: (1) subheading “The three recommended strategies” before the simulated results for the three recommended strategies; (2) subheading “Measures when applied singly” before the results of assessing the three common measures when applied singly, with different durations, frequencies and coverages for chemotherapy and different improvement rates for IEC and environmental modification; and (3) subheading “Measures when applied combinedly” before results of assessing strategies combined with two or three measures (**lines 334, 367 and 397 in the revised manuscript**). In addition, to better clarify the outputs we would like to discuss, we added subheadings to the corresponding contents in the discussion part, including: (1) subheading “Advantages of the methodology” before the discussion on the methods we used; (2) subheading “Three recommended strategies” before the discussion on the three recommended strategies; (3) subheading “Measures when applied singly or combinedly” before the discussion on the strategies derived from the three common measures; (4) subheading “Insights from sensitivity analysis” before the discussion on the results of sensitivity analysis; (5) subheading “Limitations” before the discussion of limitations in this study and (6) subheading “Conclusions” for the final conclusions (**lines 446, 468, 490, 517, 541 and 594 in the revised manuscript**).

Human1, Human2, Human3 and Human4 in the X-axis of Figure 2 in the original manuscript (**S2 Fig in the revised manuscript**) indicate human groups who seldom, moderately, often and very often eat raw or uncooked fish, respectively. We clarified this in the figure notes (**lines 764-766 in the revised manuscript**). Besides, we improved he resolution of Figure 1 (**Fig 2 in the revised manuscript)**.

Reviewer #3: (No Response)

--------------------

Conclusions
-Are the conclusions supported by the data presented?
-Are the limitations of analysis clearly described?
-Do the authors discuss how these data can be helpful to advance our understanding of the topic under study?
-Is public health relevance addressed?

Reviewer #1: The conclusions are definite and fine. However, it is a prediction, so the true figures will be know far later. Anyway, it is an interesting data and conclusion.

**Reply:**

We thank Reviewer #1 for the positive comment on the conclusions. Indeed, we applied the mathematical transmission model to assess the long-term effects of possible control strategies on *C. sinensis* infection. However, the true figures of the corresponding strategies are not available, thus we could not validate the intervention model. This limitation is stated in **lines 571-573 of the revised manuscript**.

Reviewer #2: The current conclusion is wake and a more explicit conclusion is needed such that the actual outcomes from different intervention measures can be appreciated and think about utility and their effectiveness.

**Reply:**

We added “In summary, under the typical transmission setting we applied, our simulated results suggested that combinations of measures were much beneficial; higher coverages of measures had better effects; and strategies targeted on whole population performed better than that on at-risk population with raw-fish-consumption behaviors. The strategy of Guangdong Province performed best among the three recommended ones. Sustainable strategies, such as combined measures of preventive chemotherapy (once per year for five years) with 60% coverage on whole population, IEC and environmental modification with improvement rates of 70%, or combined measures of the above preventive chemotherapy with 80% coverage on at-risk population, IEC and environmental modification with improvement rates of 85%, could achieve infection control within 10 years.” to the final part of the manuscript for more applicable conclusions (**lines 595-605 in the revised manuscript**). The detailed simulation outcomes indicating the effectiveness of studied strategies are listed in Tables 5, 6 and S3-S7 Tables for more information.

Reviewer #3: (No Response)

--------------------

Reviewer #1: This is very well designed study and also well written, and a kind of innovative way of controlling clonorchiasis in China for a long term. It may be applied to other neighboring countries having clonorchiasis/ opisthorchiasis endemic area. I suppose this mathematical model is useful to implement the control measures, although in nature it may not be achieved easily. There is human food habit, which is not easily changeable by education or knowledge.

**Reply:**

We thank the reviewer for highlighting the importance of our work. Indeed, the improvements of people’s behavior may be hard to achieve in practice. We had this comment in **lines 487-488 of the revised manuscript**.

Few questions:
1) At the end of abstract: Several sustainable strategies were provided, which could lead to infection control within 10 years. -> By any strategies, clonorchiasis can be controlled down to infection control level within 10 years? Looks too simple or some additional explanations are needed.

**Reply:**

Indeed, due to the word limit in the abstract, we cannot extend the detailed strategies we found. The potential sustainable strategies, which could lead to infection control within 10 years under our study setting, are listed in Table 6. We explained these strategies in the result part (**lines 400-416 in the revised manuscript**). Besides, we added two of these strategies, which have comparable coverage/improvement rates of the three measures, into the conclusion of the final part of the manuscript (**lines 600-605 in the revised manuscript**).

2) Too long introduction with well know facts, and too many references are there. Hope to reduce the length and numbers of references.

**Reply:**

We deleted unnecessary sentences, phrases and words in the introduction, as well as in other parts of the manuscript. **Detailed changes are provided in the final part of this letter**. In addition, the number of references is reduced to 58 compared to 73 in the original manuscript.

3) Maybe a map to show Fusha Town’s location is recommended. There is not significant moving in/out people from the Town. The Town seems to be close to the big city, not sure though. Is it not an important variable for this kind of analysis to predict the future?
Thank you!

**Reply:**

We added a map indicating locations of Fusha Town and Zhongshan City, which the town belongs to (**S1 Fig**). As our studied area is relatively independent from surroundings and the population are stable (**S1 Table**), we assumed the transmission of the disease was influenced very little by nearby areas and did not considered the interactions of disease transmission between areas. However, the distance between Fusha Town and Zhongshan City is quite close (around 29 km) and migration of population may happen, which could have an impact on the transmission. Due to unavailability of the data on how such migration may influence the transmission, we didn’t include such variable in the model to avoid large uncertainty, as the performance of models with increased complexity is critically conditional on an increase of available data for parameterization. **This limitation is put in lines 549-554 and 562-565 in the revised manuscript**.

Reviewer #2: The manuscript provided insightful modelling for design and choosing the right control strategy but overall the flow of the manuscript is difficult to follow for general reader. To increase clarity regarding the control measures and their effectiveness will be helpful.
**Reply:**

We thank the reviewer for the positive appraisal of our work. We added a flow diagram (**Fig 1 in the revised manuscript**) to depict the overall methodology of the manuscript. The categories of strategies we assessed are shown under the box of “different control strategies” in the figure, while methods to assess their effectiveness are illustrated by the sub-plot and the indicators listed in the sub-table under the box of “effectiveness of different control strategies”. Furthermore, we added a paragraph of the method overview and the subheadings in the parts of results and discussion for readers to better understand the flow of the study and to more easily find the corresponding results (**lines 165-179, 334, 367, 397, 446, 468, 490, 517, 541 and 594 in the revised manuscript**). Details of subheadings are described in the previous part of this letter.

Reviewer #3: It is recommended to reduce the length by deleting unnecessary sentences.

**Reply:**

We deleted unnecessary sentences, phrases and words, and moved some details in the methodology to the supporting information to shorten the length of the manuscript. **Detailed changes are provided in the final part of this letter**.

Line 91, 96; It is not directly infected by *C. sinenesis*, but by metacercariae of *C. sinenesis*.

**Reply:**

We thank Reviewer #3 for reading our manuscript carefully and pointing out this error. We corrected the error and other similar ones as following:

(1) We replaced the “*C. sinensis*” to “metacercariae of *C. sinensis*” in line 91 in original manuscript (**line 82 in the revised manuscript**).

(2) We replaced the “*C. sinensis*” to “metacercariae of *C. sinensis*” in line 96 in original manuscript (**line 86 in the revised manuscript**).

(3) We replaced the “*C. sinensis*” to “cercariae of *C. sinensis*” in line 237 in original manuscript (**line 233 in the revised manuscript**).

(4) We replaced the “*C. sinensis*” to “metacercariae of *C. sinensis*” in line 627 in original manuscript (**line 560 in the revised manuscript**).

Lines 237-240; Even though a part of the results is based on the studies reported findings, the survey period in references should be described.

**Reply:**

The survey period for the series of studies reported the prevalence of infected snail in different endemic areas of Guangdong province is between 2004 and 2009 (**line 236 in the revised manuscript**). We used this information as prior information for the parameter estimation.

Finally, I thought it would be better to publish it in local journals.
**Reply:**

If we understand correctly, are the local journals that Reviewer #3 referred to the ones published in China or in Guangdong Province?

Clonorchiasis is endemic not only in China, but also in other countries of East Asia (**lines 73-75 of the revised manuscript)**. Thus, the disease receives attentions not only in China, but also in other endemic countries. Even though we assessed different intervention strategies under conditions on Fusha Town, as a typical setting of *C. sinensis* transmission, the results of which can be generalized to other endemic areas with similar epidemic and under similar socioeconomic and environmental conditions. Of course, for areas far away the generalization should be taken very cautiously. Nevertheless, the multi-group model can be facilitated with epidemiological data from those areas and produce assessment results fit to their local dynamics (**lines 583-590 of the revised manuscript)**. Both the model we proposed and the results we obtained provide useful tool/information that supports the selection of control strategies on clonorchiasis by researchers and decision makers (**lines 607-609 of the revised manuscript)**. Thus, we would like to publish this work in an international journal, which is more beneficial for researchers who are interested in the disease worldwide, instead in a local journal. We would be delighted if PLOS Neglected Tropical Diseases would consider our manuscript.

--------------------

In addition, we would like to modify a few places in the manuscript as following:

1. During updating the related literatures, we found a new publication on mathematical modeling of *C. sinensis*. We revised the statements on current transmission models of clonorchiasis in the introduction part (lines 141-157 in the original manuscript) as “Four papers have been published on mathematical models depicting the transmission dynamics of *C. sinensis* infection. Dai and colleagues proposed a deterministic SI compartmental model describing the transmission of *C. sinensis* infection between human, snail, and fish. The authors also verified the globally asymptotically stable of the model in both disease-free and endemic equilibria [20]. Yuan and colleagues applied a deterministic SEIR and a SIR model to describe the spread of clonorchiasis in Foshan and Guangzhou, China, respectively [21,22]. Compared to Dai’s model, the models developed by Yuan included recovered population as well as different status (e.g., eggs and cercariae) of the parasite. Gao and colleagues proposed a SIRS model to evaluate the effects of snail control and health education on clonorchiasis control in Foshan, China [23]. These papers analyzed relationships between model parameters and the basic production number (R0), giving insights to possible directions of intervention strategies. However, no further indicators (e.g., time to achieve transmission control or extinction, percentages of prevalence decreased through time), which are more applicable for selection of specific strategies, were used for assessment. Furthermore, the models assumed homogeneity of human population, yet people with different behaviors of raw-fish consumption show different transmission patterns, which should be considered when the transmission dynamics is depicted. And control strategies focus on population with raw fish consumption instead of whole population cannot be assessed under these models.” (**lines 129-148 in the revised manuscript**). In addition, to avoid controversy, we revised the phrase “is the first effort” in lines 46 and 665 of the original manuscript to “makes the effort” (lines 46 and 605 in the revised manuscript).

2. Due to typing errors, we (1) replaced “MAD” to “MDA” in line 159 of the original manuscript (**line 150 in the revised manuscript**); (2) deleted “perturbance” in line 513 of the original manuscript (**line 450 in the revised manuscript**); (3) replaced “Chemotherapy” in **line 475 of the original manuscript** to “chemotherapy” (**line 423 of the revised manuscript**); (4) replaced “fewer people prepare” in **line 627 of the original manuscript** to “fewer people would prepare” (**lines 560-561 of the revised manuscript**); and (5) replaced “$C_{d}$, $C_{e}$ $C_{m}$” in **line 473 of the original manuscript** to “$C_{d}$, $C_{e}$ and $C_{m}$” (**line 421 of the revised manuscript**).

3. We drew new icons for human, snail and fish and replaced the ones in Figure 2 and 4 to avoid copyright issues.

4. We added figure notes to S2 Fig and S3 Fig in the original manuscript (now S4 Fig and S5 Fig in the revised manuscript, respectively) to better describe the information provided by the figures.

5. Fig 1, Fig 2 and S1 Fig in the original manuscript were renamed as Fig 2, S2 Fig and S3 Fig, respectively. S1-S6 Tables in the original manuscript were renamed as S2-S7 Tables, respectively.

6. We revised the header of Table 6 to better show the columns for strategy and the ones for effectiveness.

7. To make statements more clearly, we replaced “at the end of the interventions” in **line 387 of the original manuscript** to “at the end of the 5-year interventions” (**lines 338-339 of the revised manuscript**); and we added “the duration of chemotherapy was 5 years;” in line 473 of the original manuscript (now **line 421 of the revised manuscript**).

**Furthermore, places deleted or changed for shortening the length of the manuscript are listed as following:**

(1) We **replaced** “predominantly located in east Asia (i.e., China, South Korea, northern Vietnam, and parts of Russia). Particularly, over 85% of infected cases happened in China” in **lines 74-76 of the original manuscript** to “predominantly located in East Asia and over 85% of infected cases happened in China” (**lines 74-75 of the revised manuscript**).

(2) We **deleted** “by the International Agency for Research on Cancer (IARC)” in line 78 of the original manuscript.

(3) We **deleted** “Snails ingest the eggs of *C. sinensis* in the water, which subsequently develop to cercariae [7]. The free-swimming cercariae leave the snails and adhere to the second intermediate host, freshwater fish or shrimp, where they develop into mature metacercariae [8]. Humans and other piscivorous mammals, such as cats and dogs, get infected through consumption of raw or insufficiently cooked fish/shrimp carrying metacercariae. Inside the definitive hosts, metacercariae develop into adult flukes and live in the bile ducts [9]. The eggs are produced by adult flukes and emit with the feces into water, where a new cycle starts [10].” in lines 83-91 of the original manuscript.

(4) We **deleted** “especially in areas where raw-fish-eating behavior is common or a cultural practice, which is” in lines 94-95 of the original manuscript.

(5) We **replaced** “Among them, chemotherapy” in **line 103 of the original manuscript** to “Chemotherapy” (**line 93 of the revised manuscript**).

(6) We **deleted** “and to maintain a low prevalence in the remaining areas with prevalence less than 10%” in lines 120-121 of the original manuscript.

(7) We **replaced** “Based on the goal of the Chinese government, the government” in **lines 124-125 of the original manuscript** to “The government” (**line 113 of the revised manuscript**).

(8) We **deleted** “of the corresponding strategies” in lines 134-135 of the original manuscript.

(9) We **deleted** “starting with initial disease status” in line 140 of the original manuscript.

(10) We **deleted** “different” in line 165 of the original manuscript.

(11) We **replaced** “Let $S_{h,i}$, $I_{h,i}$, $S_{s}$, $I_{s}$, $S_{f}$ and $I_{f}$ denote the numbers of susceptible human, infected human, susceptible snail, infected snail, susceptible fish and infected fish, respectively. Here $i=1, 2, 3, 4$ indicating the human groups who seldom, moderately, often and very often eating of raw fish, respectively. Humans transfer from susceptible to infected state with the number $c_{i}\beta_{h,1}S_{h,i}I_{f}$ $(i=1, 2, 3, 4$ and $c_{1}=1$), while transfer back after recovery from individual treatment with the number $\gamma_{1}I_{h,i}$. Regards to snail and fish, they transfer from susceptible to infected state with the numbers $\beta_{s}S_{s}\left( I_{h,1}+I_{h,2}+I_{h,3}+I_{h,4} \right)$ and $\beta_{f}S_{f}I_{s}$, respectively, but do not transfer back as no treatment measures taken for intermediate hosts. Hosts enter into the system as susceptible individuals with recruitment number $\lambda$, and leave it with death rate $\mu$. We assumed same numbers of entering and leaving of the system, thus $\lambda_{j}=N_{j}\mu_{j}$ ($j=h_{i}, s,f$ and $i=1, 2, 3, 4$). Here $h_{i}, s,f$ and $N_{j}$ indicate human, snail, fish and the total number of host population, respectively, where $N_{j}=S_{j}+I_{j}$. Under conditions $S_{h,i}\geq0$, $I_{h,i}\geq0$, $S_{s}\geq0$, $I_{s}\geq0$, $S_{f}\geq0$ and $I_{f}\geq0$ and parameters nonnegative, the system has two equilibrium points: (1) the disease free equilibrium point that no disease in the population exists and (2) the endemic equilibrium point that the disease persists in the population with no change of numbers in each state.” in **lines 200-216 of the original manuscript** to “More detailed description of the model is shown in S1 Text.” (**lines 199-200 of the revised manuscript**), and moved the corresponding information to S1 Text. Therefore, we replaced the title “Ordinary differential equations of the basic transmission model.” of S1 Text in **line 882 of the original manuscript** to “The basic transmission model.” (**line 820 of the revised manuscript**).

(12) We **replaced** “The prior information and the likelihood function were described in the S4 Text. The posterior distributions of the estimated parameters were obtained through the sampling-importance-resampling procedure [48]. We randomly sampled with replacement from the prior distributions 100,000 parameter vectors, filtering out those corresponding to $R_{0}<1$ with consideration that $R_{0}$ should be greater than one in the current endemic area. Then we obtained the corresponding model outputs (i.e., the endemic equilibrium prevalence $P_{j}$ and the adjusted proportion of different human group $p_{i}^{'}$ for $j=h_{i}, s, f$ and $i=1, 2, 3, 4$) by solving the differential equations of the basic model on endemic equilibrium and calculated the corresponding likelihood values. Finally, we resampled 500 parameter vectors with replacement from the ones draw in the first step, with probabilities proportional to the posterior values (i.e., product of probabilities of the parameters according to their prior distributions and the relative likelihood values), based on which we obtained the estimated posterior prevalence of hosts. In addition, we identified the best set of estimated parameters as the one with the highest posterior values among the 500 draws [49]. All the numerical computations were performed in MATLAB R2019b.” in **lines 250-266 of the original manuscript** to “More details were described in the S4 Text.” (**lines 246-247 of the revised manuscript**), and moved the corresponding information to S4 Text. Therefore, we replaced the title “The prior information and the likelihood function of parameter estimation.” of S4 Text in **lines 888-889 of the original manuscript** to “The prior information, the likelihood function and posterior distributions of parameter estimation.” (**lines 826-827 of the revised manuscript**).

(13) We **replaced** “Local and global sensitivity were carried out to assess the influence of different parameters on the basic reproduction number $R_{0}$. Local sensitivity index was calculated according to the formula $\Upsilon_{X_{k}}^{R_{0}}=\frac{dR_{0}}{dX_{k}}\times\frac{X_{k}}{R_{0}}$, where $X_{k}$ represents parameter $k$ in the basic model [33], while partial rank correlation coeﬃcients (PRCC) were used to analyze the global sensitivity [50].” in **lines 268-272 of the original manuscript** to “Local sensitivity index [25] and partial rank correlation coeﬃcients (PRCC) [39] were calculated to assess the influence of different parameters on $R_{0}$, locally and globally, respectively.” (**lines 253-255 of the revised manuscript**).

(14) We **replaced** “A full model is show in Fig 1B, which describes transmission dynamics of *C. sinensis* infection under different control interventions. The corresponding ordinary differential equations are listed in the S2 Text. **Preventive chemotherapy** The recovery number of infected population through community chemotherapy against *C. sinensis* infection for each group of humans is expressed as $\gamma_{2,i}I_{h,i} (i=1,2,3,4)$, where $\gamma_{2,i}$ is the recovery rate of infected humans through preventive chemotherapy per unit of time, described as $\gamma_{2,i}=-\frac{\log\left( 1-C_{m,i}h \right)}{T_{i}}$ [32]. $C_{m, i}$ is the coverage of chemotherapy, $T_{i}$is the time interval of treatment in days on the $i$^th^ group of humans, and $h$ is the drug efficacy. **IEC** Through IEC, people’s healthy behaviors could be improved, which is further categorized into two kinds: (1) improvement of hygiene habits, such as washing hands before eating, using clean tableware for food and avoiding eating unclean food; and (2) changing the behavior of raw-fish-consumption, such as stopping or reducing the frequency of eating raw fish. We assumed that the first improvement will decrease the basic transmission rate (i.e., the transmission rate from an infected fish to a susceptible human who seldom eat raw fish), while the second will reduce the transmission rate of fish to at-risk population due to raw fish consumption. In such case, the basic transmission rate is expressed as $\left( {1-C}_{e1,i} \right)\beta_{h,1}(i=1,2,3,4)$ due to improvement of hygiene habits, where $C_{e1,i}$ indicates the improvement rate of hygiene habits by IEC in the $i$^th^ group of population. The transmission rate due to raw fish consumption can be written as $\left( {1-C}_{e2,i} \right)\beta_{h,i}$ $(i=2,3,4)$, where $C_{e2,i}$ indicates the rate of stopping raw-fish-eating behavior (i.e., proportions of people who stop eating raw fish among those with raw-fish-eating behavior) in the $i$^th^ group of population by IEC. These improvements were assumed continuous, that is, once it happens, it will last through time in the same population. **Environmental modification** Let $C_{d}$ denote as the coverage of sanitation toilets, which represents the proportion of people who stop defecating to fish ponds due to reconstruction of toilets adjacent. Therefore, the transmission rate from humans to snails is reduced to $\left( {1-C}_{d} \right)\beta_{s}$, which is also assumed continuous through time.” in **lines 277-305 of the original manuscript** to “In particular, two kinds of improvement through IEC were considered: (1) improvement of hygiene habits and (2) changing the behavior of raw-fish-consumption. The improvements through IEC and environmental modification were assumed continuous, that is, once it happens, it will last through time in the same population. Intervention parameters were set as following: $\gamma_{2,i}$ the recovery rate of infected humans through preventive chemotherapy, $C_{m, i}$ the coverage of chemotherapy, $T_{i}$ the time interval of treatment, $C_{e1,i}$ the improvement rate of hygiene habits, $C_{e2,i}$ the rate of stopping raw-fish-eating behavior and $C_{d}$ the coverage of sanitation toilets. $i=1,2,3,4$ represent human groups who seldom, moderately, often and very often consume raw fish, respectively. A full model is show in Fig 2B, which describes transmission dynamics of *C. sinensis* infection under different control interventions. More details are shown in S2 Text.” (**lines 260-271 of the revised manuscript**), and moved the corresponding information to S2 Text. Therefore, we replaced the title “Ordinary differential equations of the full model with interventions.” of S2 Text in **line 884 of the original manuscript** to “The full model with interventions.” (**line 822 of the revised manuscript**).

(15) We **deleted** “(i.e., improvement of hygiene habits and changing the behavior of raw-fish-consumption)” in lines 320-321 of the original manuscript.

(16) We **replaced** “The basic reproduction number $R_{0}$ was estimated to be 2.46 (95% CI: 2.31-2.56) (S1 Fig). Results for local sensitivity analysis of $R_{0}$ are show in Fig 3A, where the parameters were arranged from top to bottom according to their strength of influence on $R_{0}$. The death rates ($\mu_{h}$, $\mu_{s}$ and $\mu_{f}$) and the basic recovery rate $\gamma_{1}$ have a negative and strong influence on $R_{0}$, while the transmission rates ($\beta_{h,1}$, $\beta_{s}$ and $\beta_{f}$), the recruitments ($\lambda_{s}$, $\lambda_{f}$ and $\lambda_{h,i}$, $i=1,2,3,4$) and the ratios of transmission rates from an infected fish to a human who consumes raw fish to who seldom ($c_{2}$, $c_{3}$ and $c_{4}$) have a positive influence. Particularly, $\lambda_{h,4}$ and $c_{4}$ are much larger than other $\lambda_{h,i}$ ($i=1,2,3$) and $c_{i}$ ($i=2,3$), respectively, indicating parameters of the human group who very often consume raw fish have much stronger influence on $R_{0}$ compared to that of other human groups. The global sensitivity analysis shows similar results as the local sensitivity analysis (Fig 3B).” in **lines 364-375 of the original manuscript** to “The basic reproduction number $R_{0}$ was estimated to be 2.46 (95% CI: 2.31-2.56) (S3 Fig). Results for local and global sensitivity analysis of $R_{0}$ are show in Fig 3.” (**lines 324-326 of the revised manuscript**).

(17) We **deleted** “$R_{c}$ indicates the control reproduction number;” in line 403 of the original manuscript.

(18) We **deleted** “The black dotted lines indicate the observed baseline prevalence from survey data. The black dash dot line indicates the transmission control prevalence (i.e., 1%). The solid lines indicate results based on the best set of parameter estimates (red: WHO; green: the Chinese government; blue: the government of Guangdong Province), and the areas with lighter colors represent the corresponding 95% confidence intervals.” in lines 410-415 of the original manuscript.

(19) We **deleted** “particularly,” in **line 477 of the original manuscript**.

(20) We **replaced** “Clonorchiasis, one of the most important food-borne trematodiases around the world, is still endemic in many areas [16]. The WHO, the Chinese government and the government of Guangdong Province have set their strategies on morbidity control of the disease [22-24]. However, there haven’t been any study on evaluation of the long-term (>10 years) effects of these strategies [57,58]. And control programmes on clonorchiasis were mostly implemented less than 5 years, which is difficult to assess the long-term benefits and sustainability [17,18,25,26]. Mathematical models, depicting the transmission dynamics of diseases, provide a cost-effective way to quantitatively evaluate the long-term effects of control interventions. To our knowledge, the existing transmission models for *C. sinensis* infection ignored the different behaviors of raw-fish-consumption among human population, which are critical in the life cycle of the parasite, thus may not fully represent the transmission process. To address the above limitations” in **lines 498-510 of the original manuscript** to “In this study” (**line 447 of the revised manuscript**).

(21) We **deleted** “different groups of” in lines 511 of the original manuscript.

(22) We **replaced** “Based on the above model, we proposed a comprehensive model with interventions perturbance and simulated the long-term dynamics under different control strategies by setting different values of intervention parameters. Furthermore, with separated intervention parameters assigned for human groups with different raw-fish-consumption behaviors and for the two kinds of behavior improvements (i.e., improvement of hygiene habits and changing the behavior of raw-fish-consumption) by IEC, the intervention model allows us to investigate the control effectiveness among different human population and with different improvements of IEC.” in **lines 512-520 of the original manuscript** to “Based on the above model, we proposed a comprehensive model with interventions and simulated the long-term dynamics under various control strategies. The intervention model also allows us to investigate the control effectiveness among different raw-fish-consumption human population and with different improvements of IEC.” (**lines 449-453 of the revised manuscript**).

(23) We **moved** “To our knowledge, it is the first time that a multi-group model was proposed for transmission dynamics of *C. sinensis* infection. Values of many parameter could not be obtained from existing studies. Data-driven approaches, integrating survey data into models to estimate parameters that capture the local transmission dynamics [46], are most suitable approaches of parameter estimation for our study setting. Bayesian melding approach is one of the most common data-driven approaches [36,47,48]. It has important advantages compared to the maximum likelihood approach, including the ability to produce probability distributions of the estimated parameters and model outputs which can be used for inference and projection, and the good computational efficiency when a large number of parameters are estimated simultaneously [49]. The performance of our model estimation seems good, as the outputs were able to capture the 100% of the observed data within 95% CI” in **lines 597-609 of the original manuscript** under the “Advantages of the methodology” part in the discussion (**lines 454-465 of the revised manuscript**).

(24) We **replaced** “combination of all the three common measures,” in **lines 536-537 of the original manuscript** to “even though” (**line 484 of the revised manuscript**).

(25) We **deleted** “, which could reduce the prevalence under infection control and transmission control within 7.84 years (95% CI: 5.78-12.16) and 21.89 (95% CI: 17.80-33.81) years from the beginning of intervention, respectively. However, this strategy” in lines 537-540 of the original manuscript.

(26) We **replaced** “assess the simulated results from the corresponding scenarios.” in **line 547 of the original manuscript** to “assessed the corresponding simulated results.” (**line 492 of the revised manuscript**).

(27) We **deleted** “of simulation” in line 550 of the original manuscript.

(28) We **replaced** “results of epidemiological” in **lines 556-557 of the original manuscript** to “previous” (**line 501 of the revised manuscript**).

(29) We **deleted** “not only decrease the numbers of current infected cases but also prevent new ones,” in lines 565-566 of the original manuscript.

(30) We **replaced** “which could lead” in **line 567 of the original manuscript** to “leading” (**line 511 of the revised manuscript**).

(31) We **deleted** “(e.g., the basic reproduction number $R_{0}$)” in line 575 of the original manuscript.

(32) We **deleted** “effects than that focusing on all at-risk groups or whole population” in line 591 of the original manuscript.

(33) We **replaced** “the effects of the above interventions may change along with social and economic development in actual practice” in **lines 612-614 of the original manuscript** to “the effects may change along with social and economic development” (**lines 544-545 of the revised manuscript**).

(34) We **replaced** “Due to unavailability of data on temporal change of IEC and environmental modification, change of infection along with different seasons, infection status in nearby areas, compliance of chemotherapy in different groups of people, and effect of changing of people’s raw-fish-eating behavior on raw-fish-dish preparation, we didn’t address these potential heterogeneities in our proposed model to avoid large uncertainties” in **lines 628-633 of the original manuscript** to “Due to unavailability of the corresponding data, we didn’t address the potential heterogeneities mentioned above to avoid large uncertainties” (**lines 562-563 of the revised manuscript**).

(35) We **deleted** “on transmission of the disease” in line 637 of the original manuscript.

(36) We **deleted** “instead of other definitive hosts” in line 639 of the original manuscript.

(37) We **deleted** “under a typical transmission setting, with application of a multi-group dynamic transmission model” in lines 666-667 of the original manuscript.

(38) We **deleted** the reference [2,3], [11-13], [18-19] and [67-70] in line 76, 93, 106 and 603 of the original manuscript.

(39) Fig 2 in the original manuscript was moved to the supporting information and renamed as S2 Fig.
